# Autophagy Related 5 Promotes Mitochondrial Fission and Inflammation via HSP90‐HIF‐1*α*‐Mediated Glycolysis in Kidney Fibrosis

**DOI:** 10.1002/advs.202414673

**Published:** 2025-03-06

**Authors:** Yan Hu, Jinqing Li, Hui Chen, Yingfeng Shi, Xiaoyan Ma, Yi Wang, Xialin Li, Qin Zhong, Yishu Wang, Daofang Jiang, Shougang Zhuang, Na Liu

**Affiliations:** ^1^ Department of Nephrology Shanghai East Hospital Tongji University School of Medicine Shanghai 200120 China; ^2^ Department of Medicine Rhode Island Hospital and Alpert Medical School Brown University Providence RI 02902 USA

**Keywords:** autophagy related 5, glycolysis, heat shock protein 90, hypoxia‐inducible factor 1alpha, kidney fibrosis

## Abstract

Although significant progress in identifying molecular mediators of fibrosis is made, there is still controversy regarding the role and mechanism of autophagy in kidney fibrosis. Here, this study finds that autophagy related 5 (ATG5) is obviously increased in uric acid (UA), aristolochic acid (AA) and transforming growth factor‐*β*1 (TGF‐*β*1)‐induced HK‐2 cells, as well as in kidneys from patients with chronic kidney disease (CKD) and mice with hyperuricemic nephropathy (HN), aristolochic acid nephropathy (AAN) and unilateral renal ischemia‐reperfusion injury (uIRI). Conditional deletion of ATG5 in HN, AAN and uIRI murine models significantly alleviated aberrant glycolysis, attenuated pathological lesions, and improved kidney function. Mechanistically, ATG5 mediates the binding between heat shock protein 90 (HSP90) and hypoxia‐inducible factor 1alpha (HIF‐1*α*), thereby enhancing the stability of HIF‐1*α* and further promoting the overactivation of glycolysis. Subsequently, the aberrant glycolysis facilitated the occurrence of mitochondrial fission and inflammatory response, thus leading to kidney fibrosis. Taken together, the study provides solid evidence supporting that persistent activation of ATG5 in kidney tubules promotes kidney fibrosis. The profibrotic function of ATG5 is related to the regulation on HSP90‐HIF‐1*α*‐mediated glycolysis, resulting in mitochondrial fission and renal inflammation. Thus, ATG5 may be a novel therapeutic target for kidney fibrosis.

## Graphical abstract

When the normal kidney is subjected to injurious stimuli, it can lead to persistent activation of ATG5. The activation of ATG5 promotes HSP90‐HIF‐1*α* interaction, resulting in stabilization of HIF‐1*α* and subsequent activation of glycolysis. Consequently, this process contributes to mitochondrial fission and inflammation, ultimately culminating in kidney fibrosis.

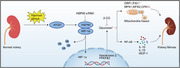

## 1 Introduction

Chronic kidney disease (CKD) has been identified as a major public health concern globally, with an estimated worldwide prevalence of 13.4%.^[^ ^1^ ^]^ Kidney fibrosis is a slow irreversible pathological process observed in multiple kidney diseases.^[^ ^2^ ^]^ It has been demonstrated that kidney fibrosis is the final common fate of patients with CKD. The characteristic of kidney fibrosis includes injury of renal tubular epithelial cells and excessive deposition of extracellular matrix (ECM).^[^ ^3^ ^]^ No effective treatment can halt the progression of CKD till now. Therefore, unveiling the exact mechanisms of kidney fibrosis will help to provide evidence to develop new therapies for treating CKD.

Autophagy is a highly conserved and precisely regulated self‐degradation process wherein forms an autophagosome, which can enclose part of the cytoplasm and damaged organelles, abnormal aggregated proteins, infected pathogens and combine with a lysosome to form an autolysosome, eventually degrades the autophagosome contents.^[^ ^4^ ^]^ The collaboration and interaction among multiple molecules encoded by autophagy related genes are essential for this process.^[^ ^5^ ^]^ In the early stages of autophagosome formation, autophagy related 5 (ATG5) occupies a pivotal position.^[^ ^6^ ^]^ Intriguingly, autophagy exhibits a dual nature, being either profibrotic or antifibrotic in kidney fibrosis. On the one hand, Li et al. reported that ATG5‐elicit protective role in kidney fibrosis by ameliorating G2/M arrest in proximal epithelial cells and participating in the degradation of collagen I.^[^ ^7^ ^]^ ATG5‐deficient cells exhibited accumulation of malformed mitochondria, increased production of reactive oxygen species (ROS), DNA damage, apoptosis in proximal tubular cells, and a decline in renal function.^[^ ^8^ ^]^ On the other hand, Dong and his colleagues demonstrated that autophagy promotes kidney fibrosis by inducing pro‐fibrotic factors such as fibroblast growth factor 2 (FGF2).^[^ ^9^ ^]^ In addition, Baisantry et al. showed that deletion of ATG5 in renal tubular can decrease kidney damage following ischemic acute kidney injury (AKI), decrease inflammation, and mitigate interstitial fibrosis.^[^ ^10^ ^]^ It can be seen the regulation of autophagy in kidney fibrosis appears to be complex and tailored to specific cell types. Therefore, it is imperative to investigate autophagy‐deficient mice in specific tissues to delineate the role of ATG5 in the progression of kidney fibrosis in the current study.

Recent literatures have emphasized the importance of metabolic reprogramming, particularly the aberration of glycolysis, which emerged as a promoter to kidney fibrosis.^[^ ^11^ ^]^ In normal physiological conditions, the tubular epithelial cells mainly depend on mitochondrial fatty acid oxidation (FAO) as their main source of energy production. However, following injurious stimuli is often accompanied by a metabolic shift to glycolysis and believed to play a crucial role in the progression of CKD.^[^ ^12^ ^]^ In the metabolic cascade of glycolysis, three enzymes serve as critical rate‐limiting factors, occurring in the sequential order of hexokinase (HK), phosphofructokinase‐1 (PFK‐1), and pyruvate kinase (PK), with each playing a pivotal role in regulating the metabolic process.^[^ ^13^ ^]^ Hypoxia‐inducible factor 1alpha (HIF‐1*α*) emerges as a pivotal regulator in the metabolic reprogramming from mitochondrial FAO to glycolysis.^[^ ^14^ ^]^ The stabilization of HIF‐1*α* participates in orchestrating the expression of various glycolytic‐related genes.^[^ ^14^, ^15^ ^]^ Previous study demonstrated that autophagy promotes glycolysis in hepatocellular carcinoma cells.^[^ ^16^ ^]^ Additionally, Yan et al. have documented that autophagy stimulates glycolysis through the enhancement of the p62/ histone deacetylase 6 (HDAC6)/HSP90 pathway.^[^ ^17^ ^]^ However, it remains unclear whether ATG5 regulates kidney fibrosis by modulating glycolysis.

Here, we explored the role of ATG5 in kidney fibrosis using both in vivo and in vitro experiments. We observed a significant upregulation of ATG5 in human tubular epithelial cells (HK‐2) stimulated with uric acid (UA), aristolochic acid (AA) and transforming growth factor‐*β*1 (TGF‐*β*1), as well as in kidney tissue samples from patients with CKD and in mice models of hyperuricemic nephropathy (HN), aristolochic acid nephropathy (AAN) and unilateral renal ischemia‐reperfusion injury (uIRI), three independent models of kidney fibrosis. This finding implicates a profibrotic role of ATG5. Tubule‐specific deletion of ATG5 alleviated kidney fibrosis via HSP90‐HIF‐1*α*‐mediated glycolysis, which further improved mitochondrial fission and inflammation. Our findings further confirmed the profibrotic role of ATG5 in kidney fibrosis.

## 2 Results

### 2.1 *ATG5* is One of the Highly Upregulated Genes in the Transcriptomic Data of UA‐Stimulated HK‐2 Cells

**Figure 1:**
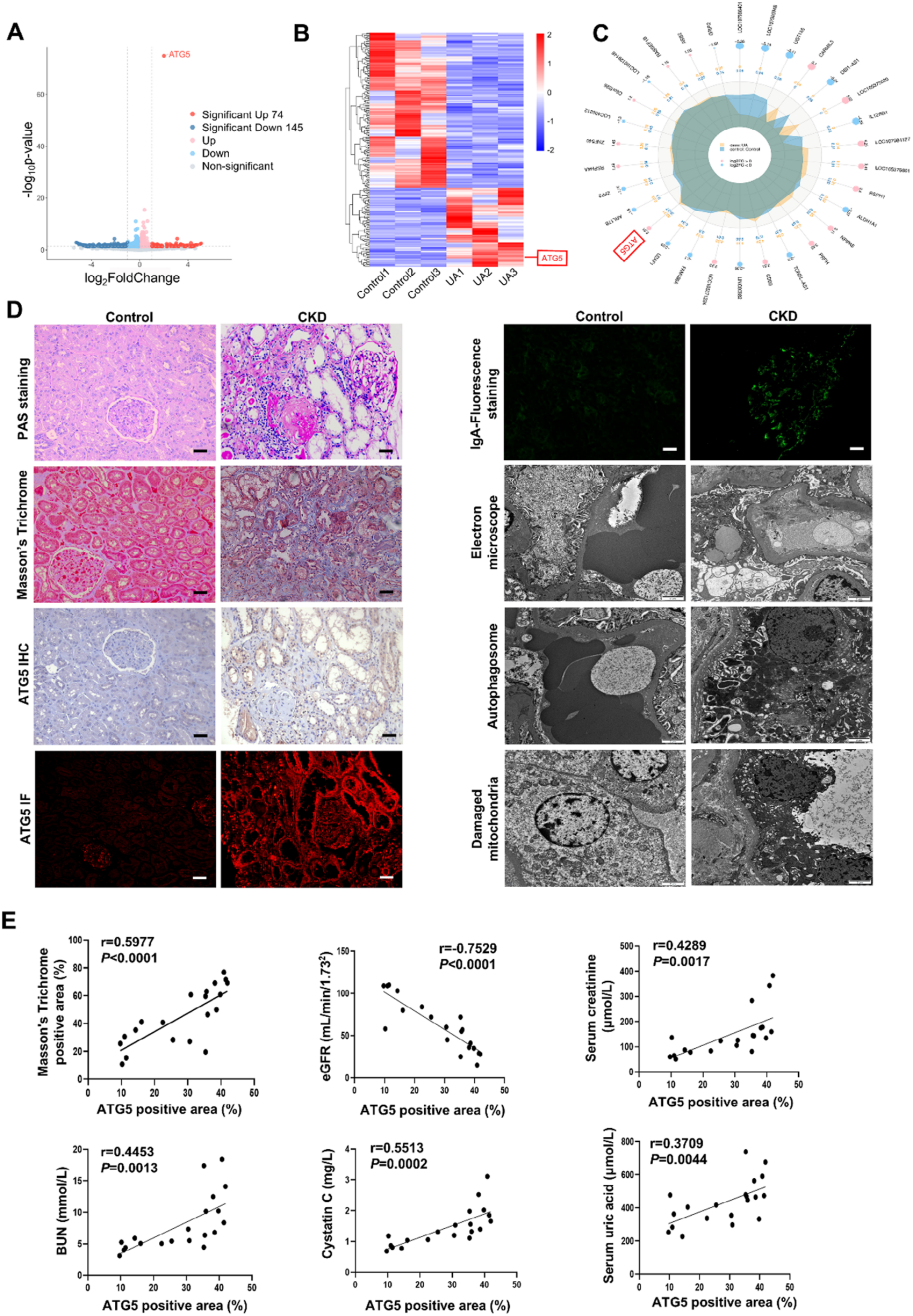
ATG5 is highly upregulated in the transcriptomic data of UA‐stimulated HK‐2 cells and human fibrotic kidneys and is positively correlated with Masson's trichrome positive area and negatively correlated with eGFR in CKD patients. A) Volcano plot showing ATG5 expression in the HK‐2 cells induced by UA (*n* = 3 for control group and UA group respectively). ATG5 was marked in the plot. B) Heat map showing all differentially expressed genes between control group and UA group. ATG5 was marked in the plot. C) radar plot showing the top 30 DEGs between control group and UA group. D) Photomicrographs of PAS staining (Scale bar = 50 µm), Masson's trichrome (Scale bar = 50 µm), ATG5 immunohistochemical staining (Scale bar = 50 µm), ATG5 immunofluorescence staining (Scale bar = 50 µm), IgA‐Fluorescence staining (Scale bar = 50 µm), and transmission electron microscopy (Scale bar = 2 µm) between control kidney tissues and renal biopsies from CKD patients (*n* = 11 for control kidney tissues, *n* = 20 for renal biopsies from CKD patients). E) Correlation between ATG5 positive area and Masson's trichrome positive area, eGFR, serum creatinine, BUN, Cystatin C, and serum uric acid in all CKD patients (*n* = 20). Data are expressed as mean ± SEM.

In order to mimic a model of HN in vitro, we induced HK‐2 cells with UA.^[^ ^18^ ^]^ Exposure of HK‐2 cells to UA (0, 200, 400, 800 µM) substantially increased expression of ATG5 and alpha‐smooth muscle actin (*α*‐SMA) in a dose‐dependent manner, with a maximum effect at 800 µM (Figure S1A‐C, Supporting Information). We also detected the expression of ATG5 and *α*‐SMA in HK‐2 cells stimulated with 800 µM UA over different periods of time (0, 12, 24, 36 h). Immunoblotting analysis showed that ATG5 and *α*‐SMA were barely detected in HK‐2 cells at 0 h, but their expression increased at 24 h and was further elevated at 36 h after UA exposure (Figure S1D‐F, Supporting Information). Therefore, we performed RNA transcriptome sequencing (RNA‐seq) to evaluate the transcriptomic profiling of HK‐2 cells stimulated with 800 µM UA for 36 h. The volcano plot showed that there were 219 Differentially Expressed Genes (DEGs) in the UA group compared to the control group, including 74 upregulated genes and 145 downregulated genes (*P*‐value<0.05 & |log_2_ FoldChange>1|) (**Figure** **1**A). It is noteworthy that *ATG5* is one of the highly upregulated genes in the UA group (Figure 1A). Consistent with this, Differential expression clustering analysis further revealed that *ATG5* was upregulated upon stimulation with UA (Figure 1B,C; Data S1, Supporting Information). Based on the fact that the *P*‐value of *ATG5* is the most significant, we further explore the role and mechanism of ATG5 in vivo and in vitro in the current study.

### 2.2 ATG5 is Highly Expressed in Human Fibrotic Kidneys and is Increased in HN Mouse Model in a Time‐Dependent Manner

Furtherly, we examined the expression of ATG5 in kidney biopsies obtained from CKD patients diagnosed with IgA nephropathy (Table S1, Supporting Information). Kidney tissue samples from patients who underwent nephrectomy due to renal carcinoma but without any other kidney diseases were used as the control group. Periodic acid‐Schiff (PAS) and Masson's trichrome staining showed that kidney tissues from patients with CKD demonstrated an obvious interstitial fibrosis. Compared with the control group, CKD samples showed a significant increased expression of ATG5 measured by immunohistochemistry staining and immunofluorescence staining. Importantly, compared with kidney tissues from control group, more autophagosomes as well as damaged mitochondria were observed in kidney biopsies from CKD patients (Figure 1D). In particular, linear regression analysis revealed that the expression of ATG5 was positively correlated with Masson's trichrome positive area, serum creatinine, blood urea nitrogen (BUN), Cystatin C, serum uric acid (SUA), and negatively correlated with estimated glomerular filtration rate (eGFR) (Figure 1E). To further evaluate the role of ATG5 in kidney fibrosis in a time‐course manner, we established an HN mouse model for 0, 7, 14, and 21 days, respectively. Masson's trichrome staining demonstrated that tubular was slightly dilated on day 7, but became more severe on days 14 and 21, with evident interstitial fibrosis and collagen deposition (Figure S2A,B, Supporting Information). Immunohistochemistry staining revealed that the expression of ATG5 was increased in a time‐dependent manner (Figure S2A,C, Supporting Information). Consistent with this observation, immunoblotting analysis also demonstrated that the expression of ATG5 and *α*‐SMA was upregulated in time‐dependent manner (Figure S2D‐F, Supporting Information). Collectively, these data suggest that ATG5 could play a profibrotic role in the process of kidney fibrosis.

### 2.3 Tubule‐Specific ATG5 Ablation Inhibits Kidney Fibrosis and Improves Tubular Function in HN Mice Model

**Figure 2:**
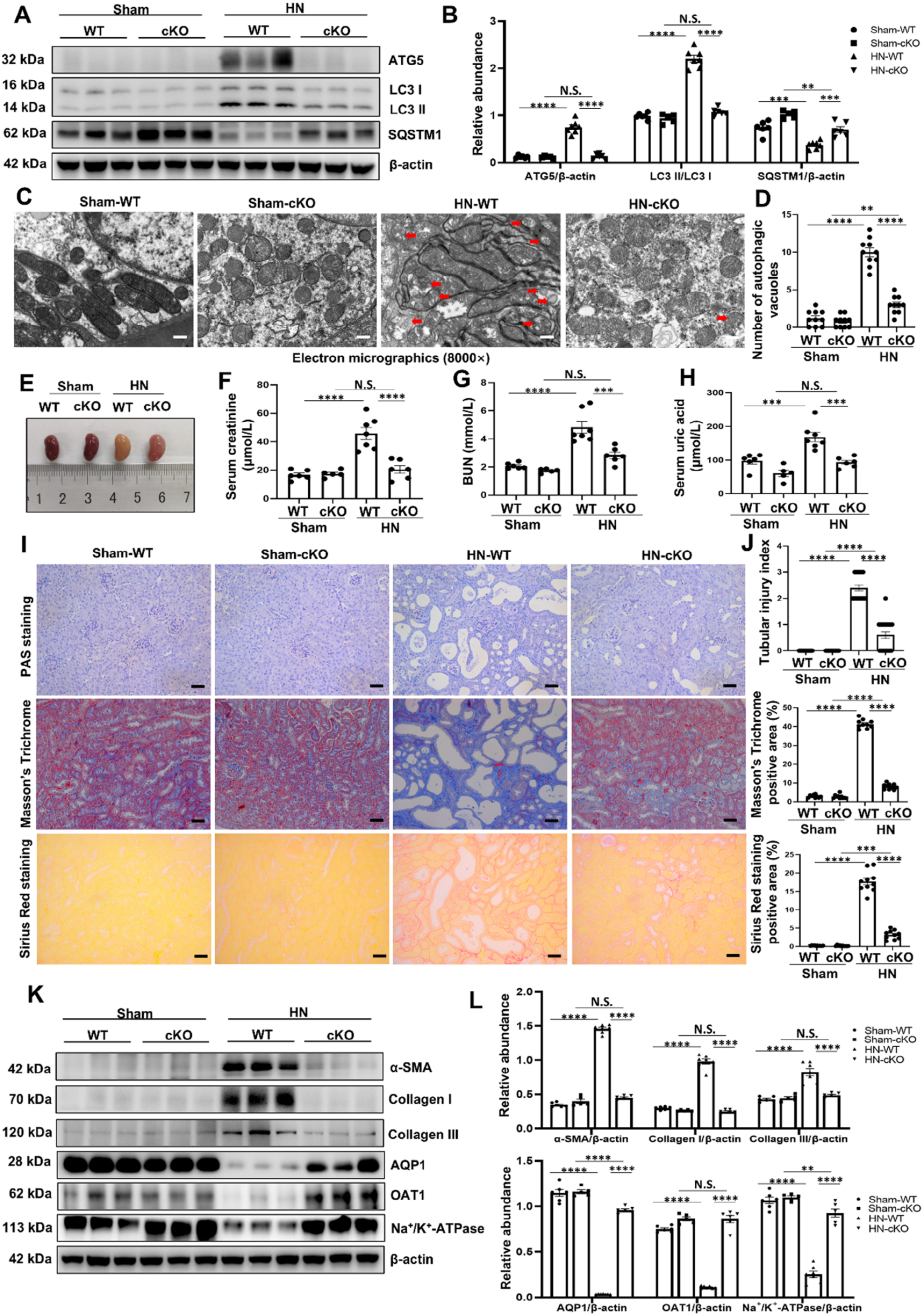
Tubule‐specific ATG5 ablation inhibits kidney fibrosis and improves tubular function in HN mice model. A) Western blot for ATG5, LC3 and SQSTM1 in kidneys from different groups of mice. B) Quantitative analyses of ATG5, LC3 II/I and SQSTM1 in kidneys from different groups of mice. C) Photomicrographs of transmission electron microscopy showing the ultrastructural feature of autophagic vacuoles (Red arrows) in kidneys from different groups of mice. Scale bar = 500 nm. D) Quantitative analyses showing the number of autophagic vacuoles per field. E) Photomicrographs showing the size, color and texture in kidneys from different groups of mice. F‐H) Serum creatinine, BUN, and serum uric acid from the mice in different groups. I,J) Representative images of PAS staining, Masson's trichrome and Sirius red staining in kidneys from different groups of mice. Tubular injury index, Masson's trichrome positive area and Sirius red staining positive area among groups as indicated. Scale bar = 50 µm. K) Western blot for *α*‐SMA, collagen I, collagen III, AQP1, OAT1, and Na^+^/K^+^‐ATPase in kidneys from different groups of mice. L) Quantitative analyses of *α*‐SMA, collagen I, collagen III, AQP1, OAT1, and Na^+^/K^+^‐ATPase standardized to *β*‐actin in kidneys from different groups of mice. *n* = 5‐7 per group. Data are expressed as mean ± SEM. **p <0.01, ***p <0.001, ****p <0.0001, and N.S. denote statistically not significant.

To explore the function of ATG5 in the development of kidney fibrosis, we generated tubule‐specific ATG5 ablation mice using a Cre‐LoxP recombination system. Briefly, Cdh16‐Cre mice were mating with ATG5^fl/fl^ mice then obtain ATG5 conditional knockout (cKO) mice. All mice utilized in the current study were genotyped by Polymerase Chain Reaction (PCR) of tail DNA (Figure S3, Supporting Information). The Cdh16‐Cre^+^: ATG5^fl/fl^ mice were phenotypically normal and have no obvious structural changes in other organs (Figure S4, Supporting Information). Next, we established a HN mice model by feeding with adenine and potassium oxonate for 21 days in the cKO mice and littermates wild‐type (WT) mice (Cdh16‐Cre^−^: ATG5^fl/fl^). Immunoblot analysis showed that the expression of ATG5 and LC3 II/I was significantly increased in HN‐WT mice. The degradation of sequestosome 1 (SQSTM1), a selective autophagy receptor and substrate, was occurred in HN mice. Tubule‐specific ATG5 ablation reduced the expression of ATG5 and LC3 II/I, and increased the expression of SQSTM1 (**Figure** **2**A,B). The transmission electron microscopy also revealed that tubule‐specific deletion of ATG5 suppressed the activation of autophagy (Figure 2C,D). Moreover, the kidney of HN‐WT mice showed a morphological change which appeared with a granular surface texture (Figure 2E). In parallel, the serum creatinine, BUN, and SUA were remarkably increased in HN‐WT mice, while tubule‐specific ATG5 ablation significantly improved the renal function (Figure 2F‐H). In addition, the histopathological examination in PAS staining, Masson's trichrome staining and Sirius red staining showed obvious tubular injury and tubulointerstitial fibrosis in HN‐WT mice, validating the successful establishment of the kidney fibrosis animal model. Tubule‐specific ATG5 ablation attenuated the pathologic damage (Figure 2I,J). Consistent with these results, immunoblot analysis demonstrated that HN‐WT mice exhibited significantly upregulation of markers related to fibrosis, such as *α*‐SMA, collagen I, collagen III, and downregulation of markers related to tubular function, such as aquaporin‐1 (AQP1), organic anion transporter 1 (OAT1), and Na^+^/K^+^‐ATPase. Tubule‐specific ATG5 ablation partially reversed these changes (Figure 2K,L). Furthermore, immunofluorescence staining indicated that ATG5 was mainly located in the cytoplasm of tubule (Figure S5A, Supporting Information). Altogether, these findings indicate that tubule‐specific ATG5 ablation alleviates kidney fibrosis and improves tubular function in HN mice model.

### 2.4 Tubule‐Specific ATG5 Ablation Alleviates Kidney Fibrosis and Improves Tubular Function in AAN and uIRI Mice Models

**Figure 3:**
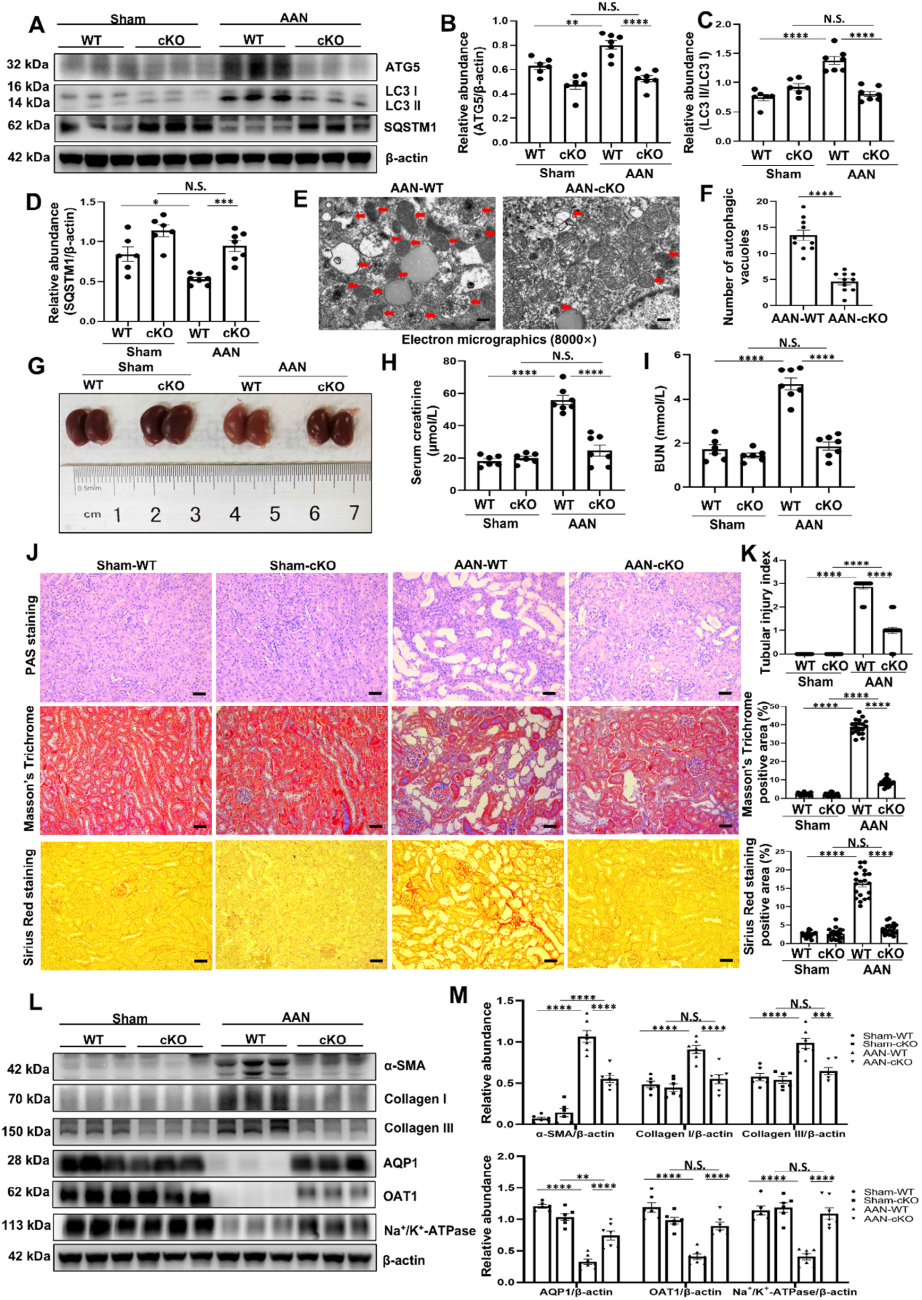
Tubule‐specific ATG5 ablation alleviates kidney fibrosis and improves tubular function in AAN mice model. A) Western blot for ATG5, LC3 and SQSTM1 in kidneys from different groups of mice. B) Quantitative analyses of ATG5 standardized to *β*‐actin in kidneys from different groups of mice. C) Quantitative analyses showing the ratio of LC3 II to LC3 I in kidneys from different groups of mice. D) Quantitative analyses of SQSTM1 standardized to *β*‐actin in kidneys from different groups of mice. E) Photomicrographs of transmission electron microscopy showing the ultrastructural feature of autophagic vacuoles (Red arrows) in kidneys from AAN‐WT and AAN‐cKO groups. Scale bar = 500 nm. F) Quantitative analyses showing the number of autophagic vacuoles per field. G) Photomicrographs showing the size, color and texture in kidneys from different groups of mice. H,I) Serum creatinine and BUN from the mice in different groups. J,K) Representative images of PAS staining, Masson's trichrome and Sirius red staining in kidneys from different groups of mice. Tubular injury index, Masson's trichrome positive area and Sirius red staining positive area among groups as indicated. Scale bar = 50 µm. L) Western blot for *α*‐SMA, collagen I, collagen III, AQP1, OAT1, and Na^+^/K^+^‐ATPase in kidneys from different groups of mice. M) Quantitative analyses of *α*‐SMA, collagen I, collagen III, AQP1, OAT1, and Na^+^/K^+^‐ATPase standardized to *β*‐actin in kidneys from different groups of mice. *n* = 6‐7 per group. Data are expressed as mean ± SEM. *p <0.05, **p <0.01, ***p <0.001, ****p <0.0001, and N.S. denote statistically not significant.

AAN and uIRI are also considered as typical models of kidney fibrosis.^[^ ^19^ ^]^ To further confirm the role of ATG5 in kidney fibrosis, we established an AAN mice model and a uIRI mice model in WT mice and cKO mice as well. As expected, the expression of ATG5 was significantly increased in AAN‐WT and uIRI‐WT mice, ablation of ATG5 in tubule reduced the expression of ATG5 and LC3 II/I, and reversed the degradation of SQSTM1 (**Figure** **3**A‐D; Figures S5B and S6A,B, Supporting Information). Similarly, ATG5 deficiency in tubule obviously decreased the autophagic vacuoles measured by transmission electron microscopy (Figure 3E,F). Moreover, tubule‐specific ATG5 ablation in AAN mice and uIRI mice attenuated the morphological changes of the kidney and improved the renal function, including serum creatinine and BUN (Figure 3G‐I; Figure S6C, Supporting Information). PAS staining, Masson's trichrome staining and Sirius red staining demonstrated that compared with sham group, the AAN‐WT mice and uIRI‐WT mice exhibited obvious interstitial fibrosis and tubular atrophy. Tubule‐specific ATG5 ablation largely alleviated the pathological injurious (Figure 3J,K; Figure S6F,G, Supporting Information). The protective effect of tubule‐specific ATG5 ablation in AAN mice and uIRI mice also manifested in the reduction of *α*‐SMA, collagen I, collagen III, and upregulation of AQP1, OAT1, and Na^+^/K^+^‐ATPase (Figure 3L,M; Figure S6D,E, Supporting Information). Thus, Tubule‐specific ATG5 ablation also alleviates kidney fibrosis and improves tubular function in AAN and uIRI mice models.

### 2.5 Gene Silence of ATG5 Inhibits Fibrosis‐Related Proteins Accumulation and Attenuates Glycolysis in HK‐2 Cells Induced by UA, AA and TGF‐*β*1

**Figure 4:**
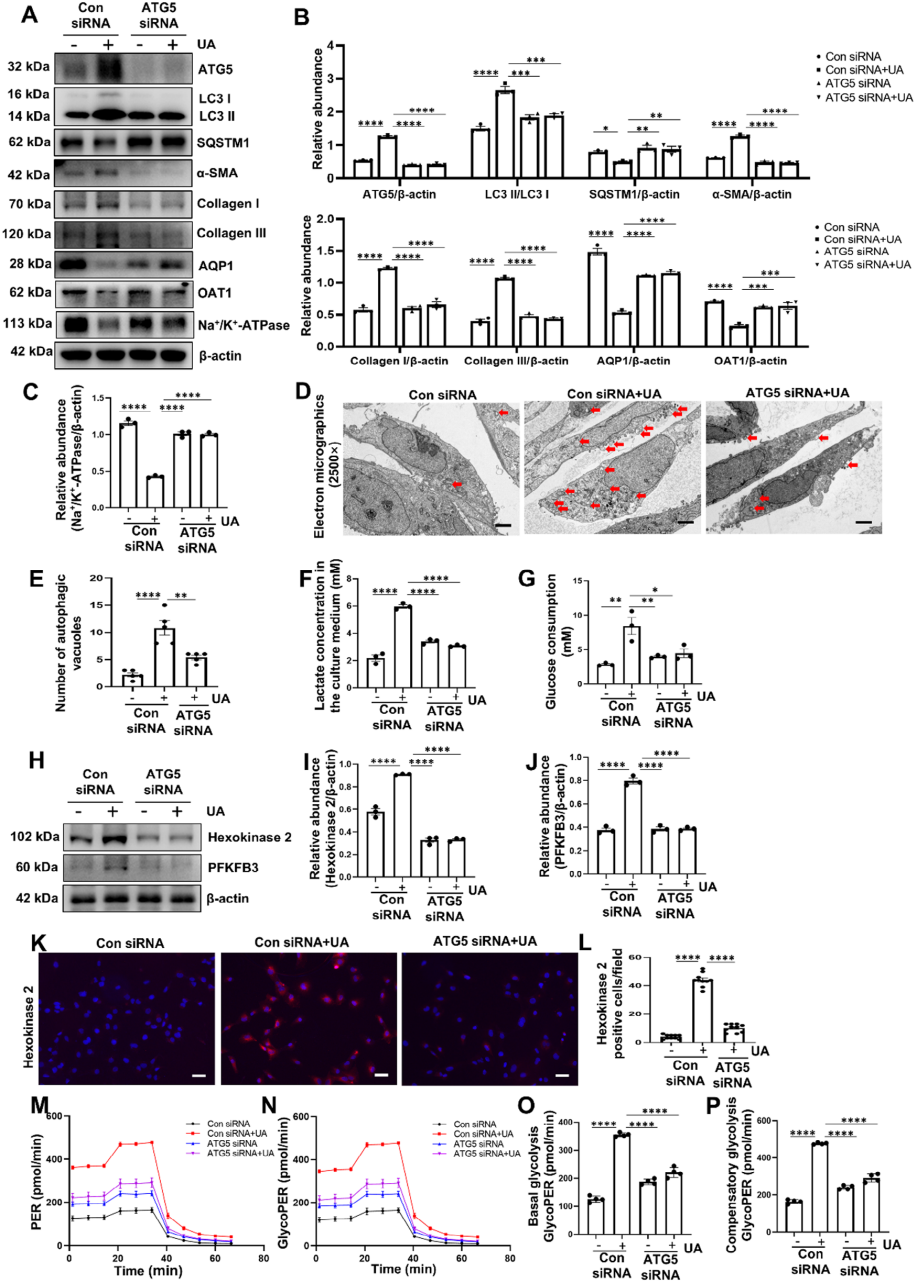
Gene silence of ATG5 inhibits fibrosis‐related proteins accumulation and attenuates glycolysis in HK‐2 cells induced by UA. A) HK‐2 cells were transfected with ATG5 siRNA or control siRNA and then incubated with or without UA (800 µM) for an additional 36 h. Representative western blot images showing the relative protein levels of ATG5, LC3, SQSTM1, *α*‐SMA, collagen I, collagen III, AQP1, OAT1, and Na^+^/K^+^‐ATPase. B,C) Quantitative analyses of ATG5, LC3 II/I, SQSTM1, *α*‐SMA, collagen I, collagen III, AQP1, OAT1, and Na^+^/K^+^‐ATPase. D) Photomicrographs of transmission electron microscopy showing the ultrastructural feature of autophagic vacuoles (Red arrows) in HK‐2 cells treated as in (A). Scale bar = 2.5 µm. E) Quantitative analyses showing the number of autophagic vacuoles per field. F) Amount of lactate in culture medium of HK‐2 cells treated as in (A). G) Glucose consumption by HK‐2 cells treated as in (A). H) Representative western blot images showing the relative protein levels of hexokinase 2 and PFKFB3 in HK‐2 cells treated as in (A). I,J) Quantitative analyses of hexokinase 2 and PFKFB3 standardized to *β*‐actin. K,L) Representative photomicrographs and quantifications showing hexokinase 2 expression in HK‐2 cells treated as in (A). Scale bar = 50 µm. M‐P) PER, and glycoPER from basal glycolysis and compensatory glycolysis were measured by Seahorse Bioscience XF96 analyzer in HK‐2 cells treated as in (A). *n* = 3‐4 per group. Data are expressed as mean ± SEM. *p <0.05, **p <0.01, ***p <0.001, ****p <0.0001.

The metabolic reprogramming which is considered as the pivotal mechanism in CKD^[^ ^12^ ^]^ inspired us to explore whether ATG5 drives the progression of kidney fibrosis via mediating glycolysis. First, we knocked down ATG5 by transfection with ATG5 small interfering RNA (siRNA), and examined the role of ATG5 in UA‐stimulated HK‐2 cells. Exposure of HK‐2 cells to UA significantly increased the expression of ATG5 and LC3 II/I and the degradation of SQSTM1. Transfection with ATG5 siRNA obviously decreased the expression of ATG5 and LC3 II/I in HK‐2 cells and increased the expression of SQSTM1, as well as reduced the autophagic vacuoles induced by UA (**Figure** **4**A‐E). UA induced an increase in the expression of *α*‐SMA, collagen I, collagen III and a decrease in the expression of AQP1, OAT1, and Na^+^/K^+^‐ATPase. Gene silence of ATG5 remarkably reduced the fibrosis‐related proteins accumulation (Figure 4A‐C). Second, we found that gene silence of ATG5 remarkably abrogated the increase of lactate concentration and glucose consumption induced by UA (Figure 4F,G), indicating that ATG5 plays an important role in glycolysis. Consistently, UA induced an increase in the expression of hexokinase 2 and 6‐phosphofructo‐2‐kinase/fructose‐2,6‐bisphosphatase 3 (PFKFB3), two enzymes associated with glycolysis, transfection with ATG5 siRNA suppressed the upregulation of hexokinase 2 and PFKFB3 (Figure 4H‐L). Furtherly, we examined the glycolytic proton efflux rate (GlycoPER) in HK‐2 cells using a glycolytic rate assay kit. As expected, we found that UA induced exacerbated metabolism as evidence by higher levels of basal and compensatory glycolysis, gene silence of ATG5 significantly inhibits the aberrant glycolysis (Figure 4M‐P). To confirm the important role of ATG5 in glycolysis, we also established in vitro models induced by AA and TGF‐*β*1. Gene silence of ATG5 successfully decreased the protein levels of ATG5 and LC3 II/I and increased the expression of SQSTM1 (Figures S7A‐D and S8A,B, Supporting Information). Transfection with ATG5 siRNA also decreased AA and TGF‐*β*1 stimulated expression of *α*‐SMA, collagen I, collagen III and reciprocally increased expression of AQP1, OAT1, and Na^+^/K^+^‐ATPase (Figures S7A,E‐J and S8C,D, Supporting Information). As expected, overexpression of ATG5 promoted the upregulation of ATG5, LC3 II/I, *α*‐SMA, collagen I, and collagen III, and decreased the expression of SQSTM1 in HK‐2 cells (Figure S9, Supporting Information). In addition, increased glycolysis was also evident in HK‐2 cells induced with AA and TGF‐*β*1, or transfected with ATG5 pcDNA3.0. This was manifested by increased levels of lactate concentration and glucose consumption rates, and upregulation of hexokinase 2 and PFKFB3 (Figure S10, Supporting Information). Gene silence of ATG5 abolished the aberrant glycolysis in HK‐2 cells (Figure S10A‐H, Supporting Information). Therefore, these results indicate that ATG5 plays an important role in glycolysis.

### 2.6 ATG5 Enhances HSP90‐HIF‐1*α* Interaction to Promote HIF‐1*α* Stabilization Resulting in Activation of Glycolysis

Based on the significant role of ATG5 in regulating glycolysis as revealed by our aforementioned findings, we further investigated the underlying mechanism of how ATG5 impacts glycolysis in HK‐2 cells. Given that HIF‐1*α* controls the expression of genes associated with glycolysis,^[^ ^14^ ^]^ we employed KC7F2, a specific inhibitor of HIF‐1*α*, to verify the role of HIF‐1*α* on glycolysis in HK‐2 cells incubated with UA. We found that treatment with KC7F2 effectively suppressed the protein expression of HIF‐1*α* (Figure S11A‐D, Supporting Information), but had no significant impact on the expression of ATG5, LC3 II/I and SQSTM1 (Figure S11E,F, Supporting Information). This might potentially be attributed to the hypothesis that HIF‐1*α* acts as a downstream effector of ATG5. In addition, both immunoblot analysis and immunofluorescence staining confirmed that KC7F2 treatment resulted in a remarkable decrease in the expression of *α*‐SMA, collagen I, collagen III, fibronectin and increase the protein levels of AQP1, OAT1, and Na^+^/K^+^‐ATPase (Figure S11G‐M, Supporting Information). As expected, both pharmacological inhibition of HIF‐1*α* and transfected with HIF‐1*α* siRNA obviously inactivated the glycolysis, as shown by decreases in lactate concentration and glucose consumption in the culture medium, a lowered protein expression of hexokinase 2 and PFKFB3, as well as prominently decreased the level of basal and compensatory glycolysis (Figures S12 and S13, Supporting Information). These results indicate that the glycolysis induced by UA stimulation is mediated by HIF‐1*α*, but it remains unclear whether ATG5 mediates glycolysis through HIF‐1*α*.

**Figure 5:**
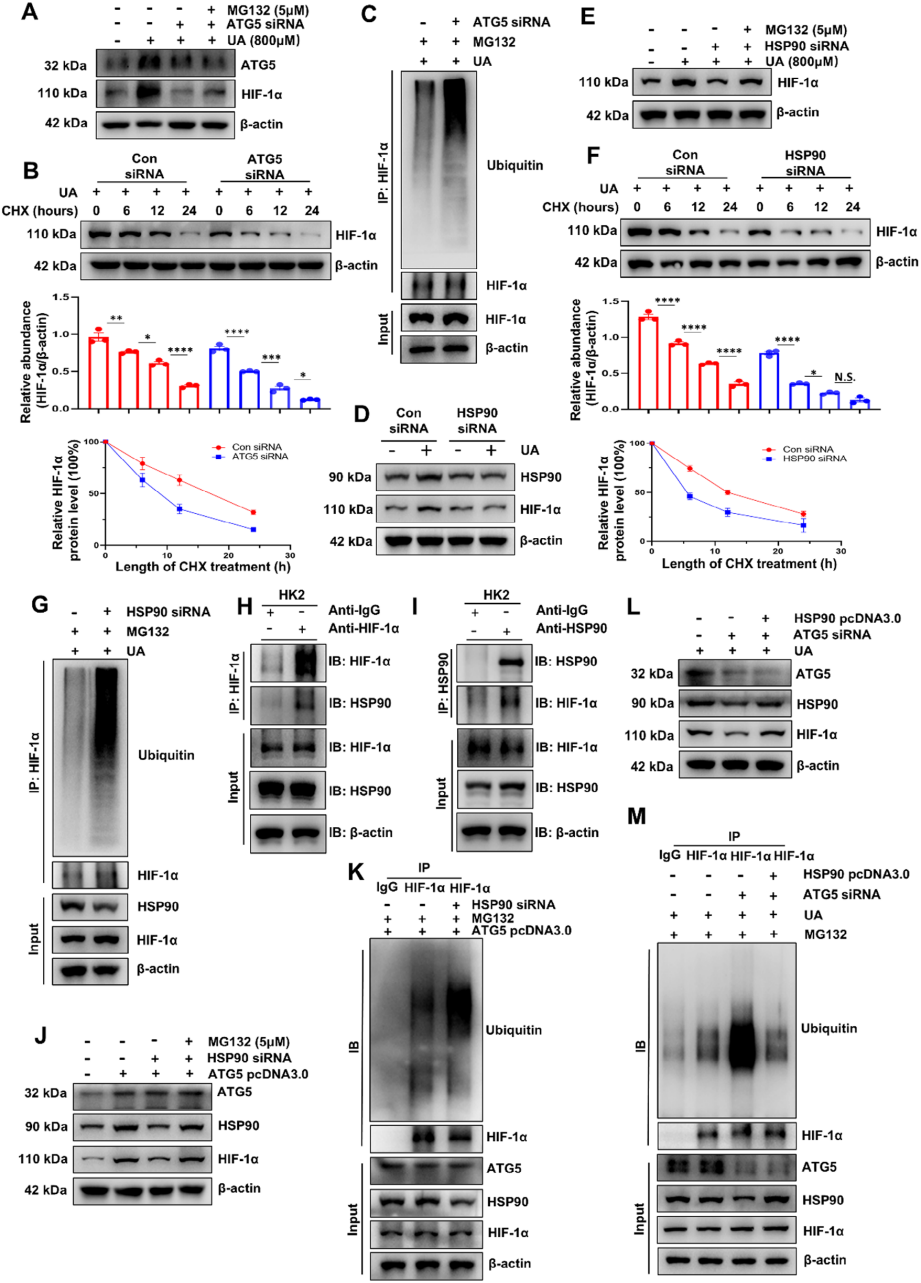
ATG5 enhances HSP90‐HIF‐1*α* interaction to promote HIF‐1*α* stabilization. A) HK‐2 cells were transfected with ATG5 siRNA or control siRNA and then incubated with or without 800 µM UA for 36 h, followed by treatment with 5 µM MG132 for 4 h before being harvested. Western blot analyses showing the expression of ATG5 and HIF‐1*α*. B) HK‐2 cells, after being transfected with ATG5 siRNA or control siRNA, were administered with CHX (10 µg mL^−1^) and harvested at the indicated times, followed by immunoblotting and quantitative analysis. C) 800 µM UA‐treated HK‐2 cells, after being transfected with ATG5 siRNA or control siRNA, were subjected to immunoprecipitation with HIF‐1*α* antibody, followed by ubiquitin immunoblotting. D) HK‐2 cells were transfected with HSP90 siRNA or control siRNA and then incubated with or without UA (800 µM) for an additional 36 h. Western blot analyses showing the expression of HSP90 and HIF‐1*α*. E) HK‐2 cells were transfected with HSP90 siRNA or control siRNA and then incubated with or without 800 µM UA for 36 h, followed by treatment with 5 µM MG132 for 4 h before being harvested. Western blot analyses showing the expression of HIF‐1*α*. F) HK‐2 cells, after being transfected with HSP90 siRNA or control siRNA, were administered with CHX (10 µg mL^−1^) and harvested at the indicated times, followed by immunoblotting and quantitative analysis. G) 800 µM UA‐treated HK‐2 cells, after being transfected with HSP90 siRNA or control siRNA, were subjected to immunoprecipitation with HIF‐1*α* antibody, followed by ubiquitin immunoblotting. H,I) 800 µM UA‐treated HK‐2 cell lysates were subjected to immunoprecipitation with IgG or HSP90, HIF‐1*α* antibodies followed by HSP90 and HIF‐1*α* immunoblotting. J) HK‐2 cells were transfected with HSP90 siRNA or control siRNA for 36 h, and then transfected with ATG5 pcDNA3.0 for an additional 36 h, followed by treatment with 5 µM MG132 for 4 h before being harvested. Western blot analyses showing the expression of ATG5, HSP90, and HIF‐1*α*. K) HK‐2 cells, after being transfected with HSP90 siRNA and ATG5 pcDNA3.0, were subjected to immunoprecipitation with HIF‐1*α* antibody, followed by ubiquitin immunoblotting. L) HK‐2 cells were transfected with ATG5 siRNA or control siRNA for 36 h, and then transfected with HSP90 pcDNA3.0 for an additional 36 h. Western blot analyses showing the expression of ATG5, HSP90, and HIF‐1*α*. M) HK‐2 cells, after being transfected with ATG5 siRNA and HSP90 pcDNA3.0, were subjected to immunoprecipitation with HIF‐1*α* antibody, followed by ubiquitin immunoblotting. *n* = 3 per group. Data are expressed as mean ± SEM. *p <0.05, **p <0.01, ***p <0.001, ****p <0.0001, and N.S. denote statistically not significant.

To investigate the potential mechanism through which ATG5 might affect glycolysis, we further examined whether ATG5 interacts with HIF‐1*α*. We found that the mRNA expression of HIF‐1*α* was not increased by UA stimulation, but the protein expression of HIF‐1*α* was increased. Gene silencing of ATG5 significantly inhibited the protein level of HIF‐1*α* (Figure S14A‐C, Supporting Information). We speculate that the difference between the mRNA and protein expression of HIF‐1*α* in UA‐stimulated HK‐2 cells might be attributed to post‐translational modifications. Given that HIF‐1*α* can be modified by ubiquitination,^[^ ^20^ ^]^ we treated the HK‐2 cells with the proteasome inhibitor MG132. We found that MG132 could reverse the downregulation of HIF‐1*α* (**Figure** **5**A; Figure S14D,E, Supporting Information). Moreover, the administration of cycloheximide (CHX), a protein synthesis inhibitor, resulted in the rapid degradation of HIF‐1*α* protein (Figure 5B). The ubiquitylation assay outcomes indicated that HIF‐1*α* underwent ubiquitylation in HK‐2 cells (Figure 5C), suggesting that the HIF‐1*α* protein is modulated by the ubiquitin‐proteasome system. Given that the chaperone HSP90 plays a critical role in modulating both the stabilization and degradation processes of HIF‐1*α*,^[^ ^21^ ^]^ we set out to explore whether HIF‐1*α* is regulated by HSP90 in the current study. Transfection with HSP90 siRNA significantly reduced the protein expression of HIF‐1*α* but had no impact on the mRNA level of HIF‐1*α* (Figure 5D; Figure S14F‐H, Supporting Information). However, the MG132 could reverse the downregulation of HIF‐1*α* (Figure 5E; Figure S14I, Supporting Information). Additionally, CHX chase assays demonstrated that transfected with HSP90 siRNA accelerated the degradation of the HIF‐1*α* protein (Figure 5F). As expected, transfection with HSP90 siRNA remarkably increased ubiquitination of HIF‐1*α* (Figure 5G), indicating that HSP90 stabilizes HIF‐1*α* by blocking its degradation through the proteasome pathway. Moreover, we performed co‐IP experiments with anti‐HSP90 and anti‐HIF‐1*α* antibodies respectively. Both HIF‐1*α* and HSP90 were detected in their individual immune pellets, but not in the negative control IgG immune pellets (Figure 5H,I). Furthermore, we set out to explore whether the regulation of HIF‐1*α* by ATG5 is associated with HSP90. We found that the mRNA and protein expression of HSP90 was obviously increased in HK‐2 cells induced by UA, gene silence of ATG5 inhibited the HSP90 upregulation (Figure S14J‐L, Supporting Information). Similarly, overexpression of ATG5 also increased the mRNA level of HSP90 in HK‐2 cells (Figure S14M, Supporting Information). In order to identify ATG5 mediated HIF‐1*α* via HSP90, we transfected with ATG5 pcDNA3.0 in HK‐2 cells. As shown in Figure 5J, overexpression of ATG5 significantly increased the expression of HSP90 and HIF‐1*α*. Transfection with HSP90 siRNA reduced the expression of HIF‐1*α*, while treatment with MG132 reversed the downregulation of HIF‐1*α*. The ubiquitylation assay also indicated that overexpression of ATG5 dramatically increased HIF‐1*α* protein stability through HSP90 (Figure 5K). In contrast, overexpression of HSP90 in ATG5 knockdown cells rescues HIF‐1*α* stability (Figure 5L,M). Importantly, the increased expression of hexokinase 2 and PFKFB3 induced by ATG5 overexpression can be reversed by HSP90 knockdown, while overexpression of HSP90 in ATG5 knockdown cells rescues the level of hexokinase 2 and PFKFB3 (Figure S15A‐F, Supporting Information). To determine whether ATG5 regulates glycolysis in an autophagy‐dependent or autophagy‐independent manner, we transfected HK‐2 cells with a plasmid expressing a mutant form of ATG5 (ATG5‐K130R), which is unable to conjugate with ATG12 and thus loses its autophagic activity.^[^ ^22^ ^]^ We found that both ATG5‐K130R and autophagy inhibitor 3‐methyladenine (3‐MA) can reduce the expression of hexokinase 2 and PFKFB3 induced by UA, indicating that glycolysis mediated by ATG5 is dependent on its function in autophagy (Figure S15G, Supporting Information). Consistently, in vivo study also found that HSP90 and HIF‐1*α* were significantly increased in the kidney of HN and AAN mice, tubule‐specific ablation abolished the upregulation of HSP90 and HIF‐1*α* (Figure S16, Supporting Information). Collectively, these results suggest that ATG5 enhances HSP90‐HIF‐1*α* interaction to promote HIF‐1*α* stabilization resulting in activation of glycolysis.

### 2.7 ATG5‐Mediated Glycolysis Promotes Mitochondrial Fission and Inflammation in HK‐2 Cells Induced by UA and AA

**Figure 6:**
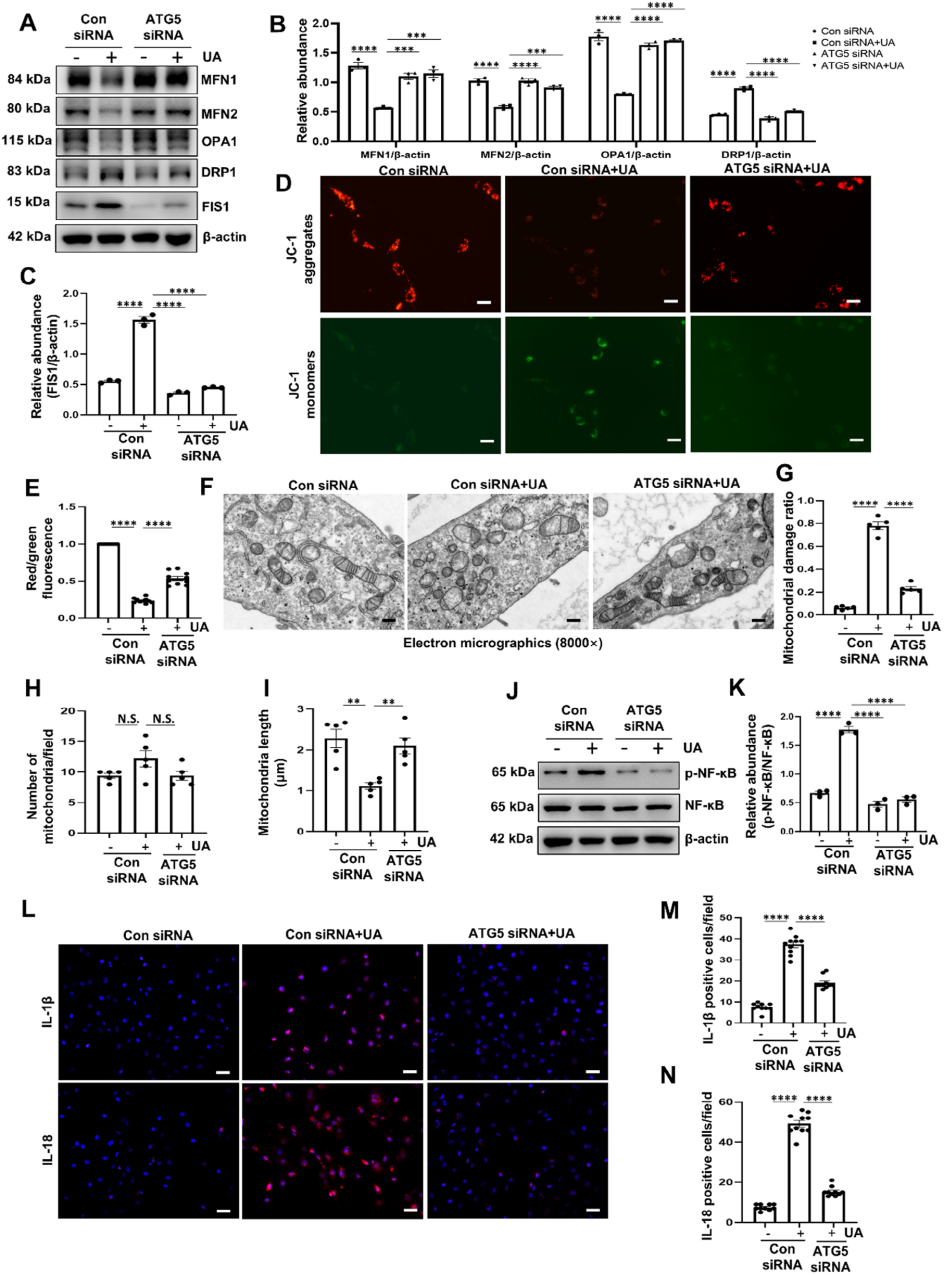
ATG5‐mediated glycolysis promotes mitochondrial fission and inflammation in HK‐2 cells induced by UA. A) HK‐2 cells were transfected with ATG5 siRNA or control siRNA and then incubated with or without UA (800 µM) for an additional 36 h. Representative western blot images showing the relative protein levels of MFN1, MFN2, OPA1, DRP1 and FIS1. B,C) Quantitative analyses of MFN1, MFN2, OPA1, DRP1 and FIS1 standardized to *β*‐actin. D) JC‐1 staining was performed to examine the mitochondrial membrane potential in HK‐2 cells treated as in (A). Scale bar = 50 µm. E) Ratio of red/green fluorescence intensity was calculated. F) Representative transmission electron microscopy images showing the ultrastructural feature of mitochondria in HK‐2 cells treated as in (A). Scale bar = 500 nm. G) Ratio of damaged mitochondria according to the transmission electron microscopy images. H) Quantitative analysis of the number of mitochondria per field. I) Quantitative analysis showing mitochondrial length in each group. J) Western blot analyses for p‐NF‐κB and NF‐κB in HK‐2 cells treated as in (A). K) Quantitative analyses showing the ratio of p‐NF‐κB to NF‐κB. L‐N) Representative photomicrographs and quantifications showing IL‐1*β* and IL‐18 expression in HK‐2 cells treated as in (A). Scale bar = 50 µm. *n* = 3 per group. Data are expressed as mean ± SEM. **p <0.01, ***p <0.001, ****p <0.0001, and N.S. denote statistically not significant.

It is reported that the hexokinase 2 glycolytic overload could drive mitochondrial dysfunction,^[^ ^23^ ^]^ and inhibition of glycolysis with 2‐deoxy‐D‐glucose (2‐DG), a glucose analogue, can reduce mitochondrial fission and increase fusion proteins.^[^ ^24^ ^]^ Therefore, we embarked on an investigation to determine whether ATG5‐mediated glycolysis exacerbates mitochondrial fission in HK‐2 cells incubated with UA and AA. Immunoblotting demonstrated that both UA and AA increased the expression of mitochondrial fission proteins, such as dynamin‐related protein 1 (DRP1) and mitochondrial fission protein 1 (FIS1), but decreased the fusion proteins, such as mitofusin 1 (MFN1), mitofusin 2 (MFN2) and optic atrophy 1 (OPA1). Gene silence of ATG5 significantly increased protein levels of mitochondrial fusion proteins and reduced fission proteins (**Figure** **6**A‐C; Figure S17A‐F, Supporting Information). Notably, the JC‐1 probe, used in detecting mitochondrial membrane depolarization, indicated that UA induced the mitochondrial depolarization, while gene silence ATG5 largely reversed the mitochondrial membrane depolarization (Figure 6D,E). Meanwhile, transmission electron microscopy revealed that UA stimulation had increased numbers of fragmented mitochondria and smaller mitochondria. ATG5 deficiency significantly attenuated the mitochondrial injury induced by UA (Figure 6F‐I). In an effort to confirm that the contribution of ATG5 to mitochondrial fission is indeed linked to HIF‐1*α*‐regulated glycolysis, we treated HK‐2 cells, which had been induced by UA, with KC7F2 or 2‐DG individually. The results showed that KC7F2 significantly alleviated mitochondrial fission, as evidenced by the increased protein levels of the fusion proteins but decreased the fission proteins, and moderated mitochondrial depolarization (Figure S18, Supporting Information). Treatment with 2‐DG effectively inhibited the upregulation of lactate concentration and glucose consumption in the culture medium, as well as ameliorated the protein levels of hexokinase 2 and PFKFB3 induced by UA (Figure S19A‐G, Supporting Information). In parallel, administration with 2‐DG could inhibit the UA‐induced the increased expression of fibrosis associated proteins and the decreased of AQP1, OAT1, and Na^+^/K^+^‐ATPase (Figure S19H‐N, Supporting Information). More importantly, treatment with 2‐DG significantly reduced the mitochondrial fission and ameliorated the mitochondrial membrane depolarization (Figure S20, Supporting Information).

Considering that glycolysis has been served as a crucial player in the inflammatory response,^[^ ^25^ ^]^ we also examined whether ATG5‐mediated glycolysis promotes inflammation in HK‐2 cells incubated with UA and AA. Western blot analysis indicated that transfection with ATG5 siRNA inhibits the activation of NF‐κB pathway in HK‐2 cells incubated with UA and AA (Figure 6J,K; Figure S17G‐I, Supporting Information). Meanwhile, gene silence of ATG5 blockades the increased expression levels of interleukin‐1 beta (IL‐1*β*) and interleukin‐18 (IL‐18) induced by UA (Figure 6L‐N). Similarly, treatment with either KC7F2 or 2‐DG have been shown to alleviate inflammation, as evidenced by inactivating the NF‐κB pathway, and decreasing the fluorescence intensity of IL‐1*β* and IL‐18 (Figures S21 and S22, Supporting Information). Collectively, these data show that ATG5‐mediated glycolysis promotes mitochondrial fission and inflammation in HK‐2 cells induced by UA and AA.

### 2.8 Tubule‐Specific ATG5 Ablation Ameliorates Glycolysis‐Mediated Mitochondrial Fission and Inflammation in HN Mice

**Figure 7:**
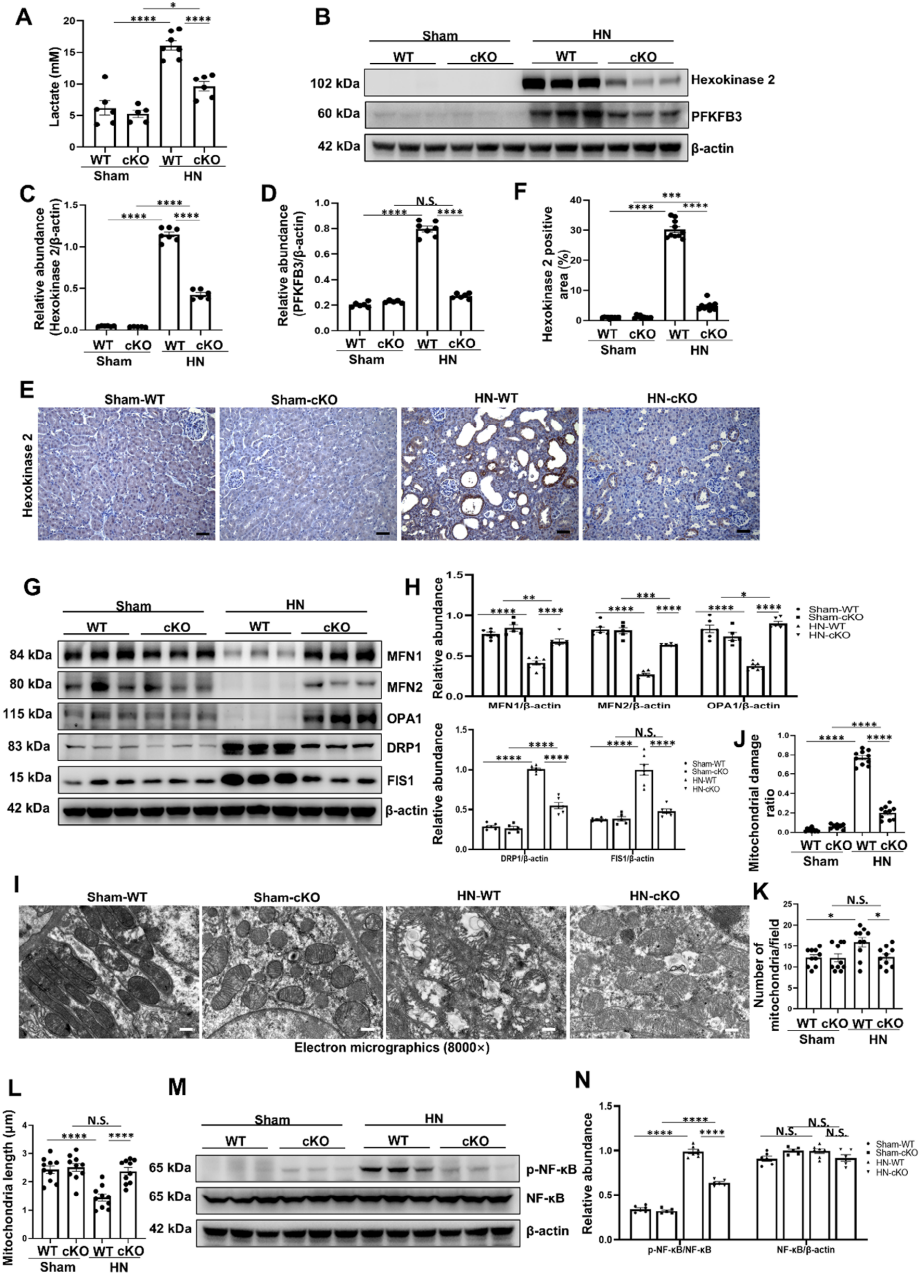
Tubule‐specific ATG5 ablation ameliorates glycolysis‐mediated mitochondrial fission and inflammation in HN mice. A) The level of serum lactate from different groups of mice. B) Western blot for hexokinase 2 and PFKFB3 in kidneys from different groups of mice. C,D) Quantitative analyses of hexokinase 2 and PFKFB3 standardized to *β*‐actin in kidneys from different groups of mice. E,F) Representative photomicrographs and quantifications showing hexokinase 2 expression in kidneys from different groups of mice. Scale bar = 50 µm. G) Western blot for MFN1, MFN2, OPA1, DRP1 and FIS1 in kidneys from different groups of mice. H) Quantitative analyses of MFN1, MFN2, OPA1, DRP1 and FIS1 standardized to *β*‐actin in kidneys from different groups of mice. I) Representative transmission electron microscopy images showing the ultrastructural feature of mitochondria in kidneys from different groups of mice. Scale bar = 500 nm. J) Ratio of damaged mitochondria according to the transmission electron microscopy images. K) Quantitative analysis of the number of mitochondria per field. L) Quantitative analysis showing mitochondrial length in each group. M) Western blot analyses for p‐NF‐κB and NF‐κB in kidneys from different groups of mice. N) Quantitative analyses showing the ratio of p‐NF‐κB to NF‐κB and NF‐κB standardized to *β*‐actin in kidneys from different groups of mice. *n* = 5–7 per group. Data are expressed as mean ± SEM. *p <0.05, **p <0.01, ***p <0.001, ****p <0.0001, and N.S. denote statistically not significant.

Based on the in vitro evidence for the regulatory role of ATG5 on glycolysis, we further investigated whether ATG5 can also exert a similar influence on glycolysis in vivo. The results showed that tubule‐specific ATG5 ablation significantly decreased the serum lactate in HN mice (**Figure** **7**A). Compared with the sham group, the protein levels of hexokinase 2 and PFKFB3 were strongly increased in HN mice, conditional knockout of ATG5 significantly inhibits glycolysis (Figure 7B‐F). Importantly, tubule‐specific ATG5 ablation alleviates the mitochondrial fragmentation, as evidenced by the upregulation of fusion proteins and reduction of mitochondrial fission proteins (Figure 7G,H). Simultaneously, transmission electron microscopy showed structural abnormalities including mitochondrial swelling and disturbance or loss of mitochondrial cristae in HN‐WT mice, while ATG5 deficiency significantly improved mitochondrial damage (Figure 7I‐L). On the other hand, we also found that tubule‐specific ATG5 ablation inhibits the NF‐κB pathway activation, as well as reduced the expression of inflammatory cytokines, including monocyte chemoattractant protein‐1 (MCP‐1), IL‐1*β* and IL‐18 (Figure 7M,N; Figure S23, Supporting Information).

To further confirm the role of ATG5 in promoting glycolysis induced mitochondrial fission and inflammation, we established a murine model of HN treated with the glycolysis inhibitor 2‐DG. Administration of 2‐DG dramatically decreased the protein level of hexokinase 2, and alleviated the upregulation of serum lactate, creatinine, BUN and uric acid (Figure S24A‐H, Supporting Information). Meanwhile, 2‐DG ameliorated kidney fibrosis in HN evidenced by the results of PAS, Masson's trichrome and Sirius red staining (Figure S24I,J, Supporting Information). Immunoblotting also indicated that 2‐DG suppressed the fibrosis‐related proteins and upregulated the expression of AQP1, OAT1, and Na^+^/K^+^‐ATPase (Figure S24K,L, Supporting Information). Predictably, treatment with 2‐DG significantly attenuated mitochondrial fission and inflammation in HN mice, as manifested by the upregulation of mitochondrial fusion proteins and downregulation of fission proteins, and decreased the ratio of damaged mitochondria, as well as inactivated the NF‐κB pathway (Figure S25, Supporting Information). These data indicate that tubule‐specific ATG5 ablation ameliorates glycolysis‐mediated mitochondrial fission and inflammation in HN mice.

### 2.9 Tubule‐Specific ATG5 Ablation Ameliorates Mitochondrial Fission and Inflammation in AAN Mice

**Figure 8:**
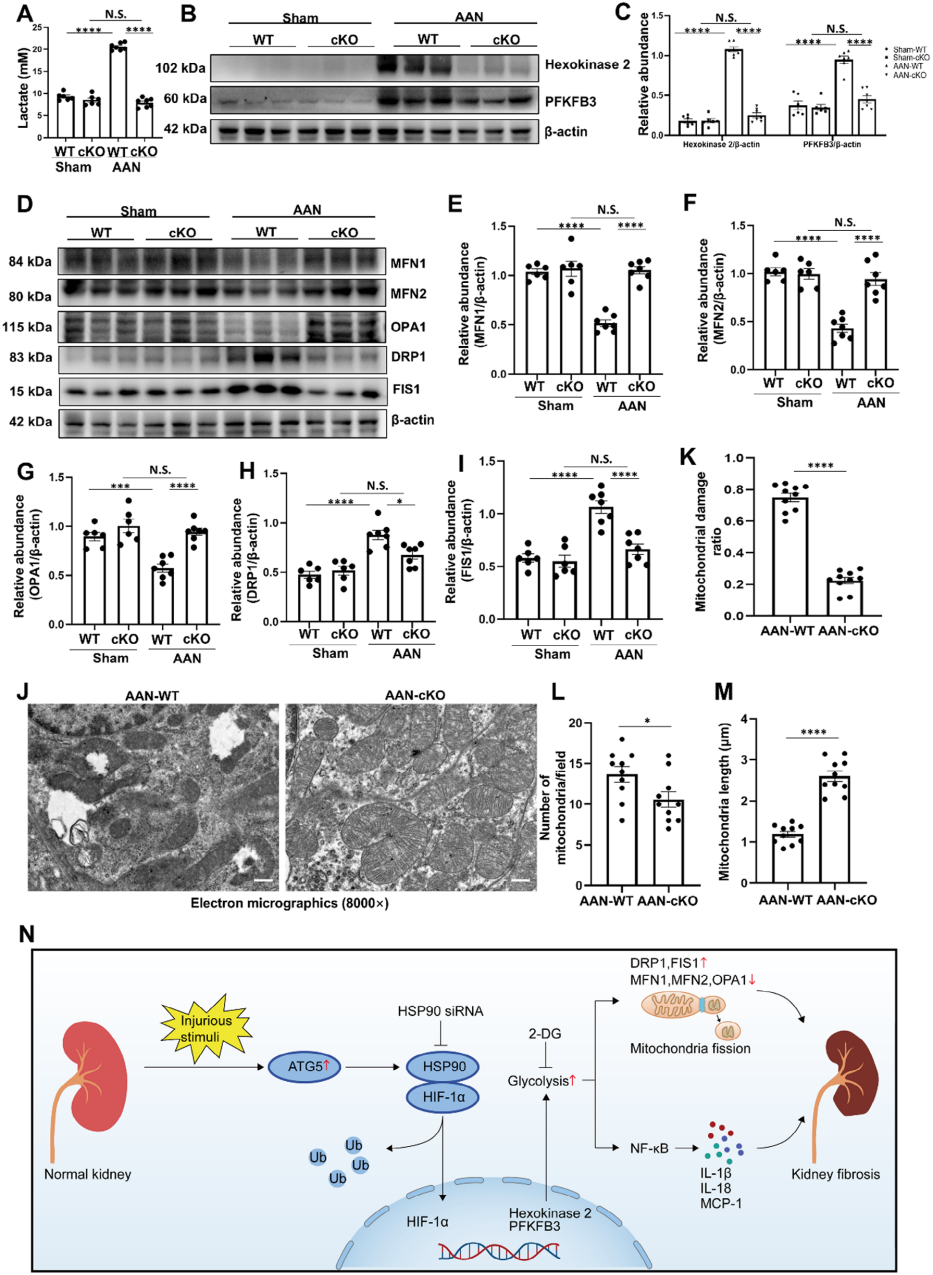
Tubule‐specific ATG5 ablation ameliorates mitochondrial fission in AAN mice. A) The level of serum lactate from different groups of mice. B) Western blot for hexokinase 2 and PFKFB3 in kidneys from different groups of mice. C) Quantitative analyses of hexokinase 2 and PFKFB3 standardized to *β*‐actin in kidneys from different groups of mice. D) Western blot for MFN1, MFN2, OPA1, DRP1 and FIS1 in kidneys from different groups of mice. E‐I) Quantitative analyses of MFN1, MFN2, OPA1, DRP1 and FIS1 standardized to *β*‐actin in kidneys from different groups of mice. J) Representative transmission electron microscopy images showing the ultrastructural feature of mitochondria in kidneys from AAN‐WT and AAN‐cKO groups. Scale bar = 500 nm. K) Ratio of damaged mitochondria according to the transmission electron microscopy images. L) Quantitative analysis of the number of mitochondria per field. M) Quantitative analysis showing mitochondrial length in each group. N) Schematic depicting persistent activation of ATG5 promotes mitochondrial fission and inflammation via HSP90‐HIF‐1*α*‐mediated glycolysis in kidney fibrosis. In pathological condition, the persistent activation of ATG5 promotes HSP90‐HIF‐1*α* interaction, resulting in stabilization of HIF‐1*α* and subsequent activation of glycolysis. Consequently, this process contributes to mitochondrial fission and inflammation, ultimately culminating in kidney fibrosis. *n* = 6–7 per group. Data are expressed as mean ± SEM. *p <0.05, ***p <0.001, ****p <0.0001, and N.S. denote statistically not significant.

We further verify the role of ATG5 on mitochondrial fission and inflammation in AAN mice. The serum lactate was significantly increased in AAN‐WT mice, conditional knockout of ATG5 obviously reduced the lactate concentration (**Figure** **8**A). Consistently, tubule‐specific ATG5 ablation attenuated the protein expression of hexokinase 2 and PFKFB3 (Figure 8B,C; Figure S6H‐J, Supporting Information). As expected, tubule‐specific ATG5 ablation reduced the protein expression of DRP1 and FIS1, and increased the levels of MFN1, MFN2 and OPA1, as well as improved mitochondrial damage (Figure 8D‐M). In addition, Immunoblotting and immunochemistry staining showed that ATG5 deficiency abolished the phosphorylation of NF‐κB, and reduced the expression of MCP‐1, IL‐1*β* and IL‐18 in AAN (Figure S26, Supporting Information). These data indicate that tubule‐specific ATG5 ablation ameliorates mitochondrial fission and inflammation in AAN mice model.

In short, when the normal kidney is subjected to UA, AA and TGF‐*β*1 stimulation, it can lead to persistent activation of ATG5. The activation of ATG5 promotes HSP90‐HIF‐1*α* interaction, resulting in stabilization of HIF‐1*α* and subsequent activation of glycolysis. Consequently, this process contributes to mitochondrial fission and inflammation, ultimately culminating in kidney fibrosis (Figure 8N).

## 3 Discussion

Autophagy, an evolutionarily conserved cellular process, has an extensive array of associations with cellular homeostasis and human disease.^[^ ^26^ ^]^ Our current results highlight that autophagy plays an important role during kidney fibrosis. We found that ATG5 expression was significantly increased in fibrotic kidneys from CKD patients and three mouse models of kidney fibrosis including HN, AAN and uIRI, as well as in HK‐2 cells induced by UA, AA and TGF‐*β*1. Mechanistically, our group demonstrate that ATG5 enhances HSP90‐HIF‐1*α* interaction to promote HIF‐1*α* stabilization resulting in activation of glycolysis, which further contributes to mitochondrial fission and inflammation. Conversely, tubule‐specific deletion of ATG5 obvious attenuates the kidney fibrosis. Moreover, we discovered that the deficiency of ATG5 mitigated cell cycle arrest and apoptosis in HN mice (Figure S27, Supporting Information). Our research indicated that ATG5 is a pathogenic factor exerting a significant impact in the development of kidney fibrosis.

In spite of extensive investigations during the past decade, the function of autophagy in renal tubular cells concerning its involvement in renal interstitial fibrosis is still controversial, with evidence supporting both pro‐fibrotic and anti‐fibrotic activities.^[^ ^27^ ^]^ Baisantry and colleagues demonstrated that the genetic deletion of ATG5 specifically in proximal tubular S3 segment resulted in improved recovery from ischemic acute kidney injury, characterized by reduced inflammation, diminished accumulation of senescent cells, and attenuated interstitial fibrosis. This finding suggests that persistent activation of tubular autophagy could potentially exacerbate kidney fibrosis during maladaptive kidney repair processes.^[^ ^10^ ^]^ Besides, in a recent investigation, Livingston et al. utilized a tubule‐specific autophagy related 7 (ATG7) knockout mouse model where tubular cell autophagy is specifically suppressed during kidney recovery, providing robust evidence that persistent tubular autophagy drives interstitial fibrosis development. Autophagy might orchestrate a series of additional stress responses in tubular cells, including dedifferentiation, cellular senescence, and G2/M phase arrest, to facilitate a transformation into a secretory phenotype, thereby contributing to the production of pro‐fibrotic FGF2.^[^ ^9b^ ^]^ Not long afterwards, their research team further revealed autophagy promotes kidney fibrosis by SQSTM1/p62‐mitogen‐activated protein kinases (MAPK)/extracellular signal‐regulated kinase (ERK)‐early growth response 1 (EGR1)‐FGF2 signaling cascade.^[^ ^9a^ ^]^ Consistent with these reports, our in vivo study demonstrated that tubule‐specific ATG5 ablation alleviated kidney fibrosis in HN, AAN and uIRI mice models. However, in murine model of unilateral ureteral obstruction (UUO), ATG5 deficiency in proximal tubular epithelial cells aggravated kidney fibrosis by regulating G2/M cell cycle arrest and NF‐κB signaling.^[^ ^7^, ^28^ ^]^ The discrepancy between our research and Mao et al.^[^ ^28^ ^]^ may be attributable to the differences of the models used. They demonstrated a 7‐day autophagy induction in proximal tubules during the UUO period, whereas in our HN, AAN and uIRI models, autophagy induction was persistent and sustained throughout the entire 21‐day duration. Therefore, persistent activation of ATG5 may promote the progression of kidney fibrosis. Despite the current study showing the upregulation of ATG5 during HN, AAN and uIRI, the upstream signaling that stimulates the activation of autophagy remains unclear. Our previous study indicated that UA may activate autophagy by regulating the p53 signaling pathway.^[^ ^29^ ^]^ Further efforts are needed to address this issue in our future studies.

A metabolic switch from oxidative phosphorylation to glycolysis that was primarily identified in neoplastic cells was termed the “Warburg effect”.^[^ ^30^ ^]^ This process facilitates in activating and proliferating of various cells, such as fibroblasts, macrophages, and T cells.^[^ ^31^ ^]^ Enhanced glycolysis was also observed in fibrotic kidneys,^[^ ^31^, ^32^ ^]^ which aligns with our current findings. We found that lactate concentration and the protein levels of hexokinase 2 and PFKFB3 were significantly increased in the fibrotic kidneys from HN, AAN and uIRI mice. UA stimulation exacerbated metabolism as evidence by higher levels of basal and compensatory glycolysis in HK‐2 cells. The accumulation of lactate has been shown to induce the expression of TGF‐*β* and promote vascular endothelial growth factor (VEGF) activity.^[^ ^33^ ^]^ A lactate‐enriched environment is known to trigger fibroblast activation and expedite the process of collagen accretion.^[^ ^34^ ^]^ Furthermore, we revealed that genetic deletion of ATG5 obviously attenuated glycolysis. It has been demonstrated that autophagy facilitates glucose uptake and utilization, and conversely, when autophagy is suppressed, there is an evident decline in glucose uptake.^[^ ^35^ ^]^ Autophagy serves a pivotal function in sustaining glycolytic processes within hematological malignancies, such as chronic myeloid leukemia.^[^ ^36^ ^]^ It involves the degradation of cellular constituents that are subsequently utilized as carbohydrate substrates for glycolysis.^[^ ^37^ ^]^

Meanwhile, Yao et al. showed that autophagy facilitated glycolysis under hypoxic conditions.^[^ ^38^ ^]^ Thus, we also explored the mechanism by which ATG5 promotes glycolysis in the current study. Considering that HIF‐1*α* controls the expression of glycolytic genes, we confirmed that genetic deletion of ATG5 inhibits HIF‐1*α*. Inhibition of HIF‐1*α* obviously decreased glycolysis enzyme expression and attenuated glycolytic proton efflux rate. These results imply that ATG5 promotes glycolysis associated with HIF‐1*α*. It is documented that a set of positive modulators, including coactivator molecules and molecular chaperones, contributes significantly to the transcriptional activity and stability of HIF‐1*α* under both hypoxic and normoxic environments.^[^ ^39^ ^]^ HSP90 is among the most prevalent molecular chaperone proteins that actively participate in the processes of protein folding and stabilization.^[^ ^40^ ^]^ HSP90 can interact with HIF‐1*α* in vivo and in vitro by binding to HIF‐1*α* Per‐ARNT‐Sim domain, and this interaction is essential for the stabilization of the HIF‐1*α* protein.^[^ ^41^ ^]^ The data from our present study demonstrate that transfection with HSP90 siRNA leads to a lessened interaction between HSP90 and HIF‐1*α* in HK‐2 cells, thus accounting for the decreased stability of the HIF‐1*α* protein. The observed effect is in accordance with the widely recognized principle that the molecular association between HSP90 and HIF‐1*α* plays a decisive role in regulating the abundance of HIF‐1*α* protein.^[^ ^42^ ^]^ ATG5 is a key component of the autophagy machinery, and its knockdown can disrupt the autophagic process. Autophagy has been shown to influence the expression of heat shock proteins through its role in cellular stress response and protein homeostasis.^[^ ^43^ ^]^ The expression of HSP90 is regulated by HSFs and other transcription factors that bind to specific elements in the HSP90 promoter region.^[^ ^44^ ^]^ Under stress conditions, such as UA stimulation, the activation of these transcription factors may lead to the upregulation of HSP90 mRNA. Therefore, our results may indicate that ATG5 enhances HSP90‐HIF‐1*α* interaction to promote HIF‐1*α* stabilization resulting in activation of glycolysis.

Notably, despite its initial role in sparing oxygen and maintaining ATP synthesis, glycolysis eventually exerts a detrimental impact on mitochondrial function over the long term.^[^ ^45^ ^]^ Increasing evidence indicates that alterations in cellular metabolism can have a significant impact on mitochondrial function.^[^ ^46^ ^]^ The metabolic regulation of renal fibrosis is believed to be intricately linked to the modulation of mitochondrial function.^[^ ^47^ ^]^ Mitochondria are highly dynamic organelles, through fusion and fission, mitochondrial morphology can change from elongated tubes to small, less connected mitochondria.^[^ ^48^ ^]^ We found that ATG5‐mediated glycolysis promotes mitochondrial fission, as evidenced by a significant upregulation of DRP1 and FIS1, concomitant with downregulation of MFN1, MFN2 and OPA1. Our results are in consistent with previous studies.^[^ ^24^, ^49^ ^]^ Particularly, enhancement of mitochondrial fission served a contribution role in the maintenance of cell proliferation and migration,^[^ ^50^ ^]^ which might promote the progression of kidney fibrosis.

In addition, glycolysis has been considered as a pivotal player in the inflammatory response.^[^ ^25^ ^]^ HIF‐1*α*‐induced glycolysis in mammary epithelial cells exacerbates inflammation. Conversely, inhibiting HIF1*α*‐mediated glycolysis with PFK15 or 2‐DG effectively reduces the production of proinflammatory cytokines.^[^ ^51^ ^]^ Accumulating evidence supports that glycolysis promotes inflammation. For instance, enhanced glycolysis contributes to the maturation of dendritic cells in response to toll‐like receptor ligands.^[^ ^52^ ^]^ Pretreatment with PFKFB3 inhibitor 3PO partly reduced the elevated expression of tumour necrosis factor alpha (TNF‐*α*), interleukin 6 (IL‐6) and IL‐1*β*.^[^ ^53^ ^]^ In vivo data showing that PFKFB3 suppression through 3PO normalized inflammation in mesenteric endothelium triggered by lipopolysaccharide (LPS), coupled with in vitro results indicating that either blocking or enhancing PFKFB3 expression can dampen or escalate inflammation in inflamed endothelial cells (ECs), collectively suggested that glycolysis drives inflammation.^[^ ^54^ ^]^ Mechanistically, previous research has shown that the inhibition of PFKFB3 ameliorates inflammation in ECs by impeding the nuclear translocation of p65 protein and thereby diminishing the transcriptional activity in the NF‐κB signaling pathway.^[^ ^54^, ^55^ ^]^ In line with these reports, our current study also demonstrated that ATG5‐mediated glycolysis promotes inflammation in HK‐2 cells induced by UA and AA, and in fibrotic kidneys from HN and AAN mice, as evidenced by regulating the phosphorylation of NF‐κB, and increased the expression of MCP‐1, IL‐1*β* and IL‐18. Therefore, target to the ATG5‐mediated glycolysis signaling axis may also a novel anti‐inflammatory mechanism in kidney fibrosis.

In summary, the findings of this study demonstrated that persistent activation of ATG5 promotes HSP90‐HIF‐1*α* interaction, resulting in stabilization of HIF‐1*α* and subsequent activation of glycolysis. Consequently, this process contributes to mitochondrial fission and inflammation, ultimately culminating in kidney fibrosis. Knowledge gained from this study may offer a novel rationale for developing therapeutic strategies for the treatment of kidney fibrosis.

## 4 Experimental Section

### Cell Culture and Treatments

HK‐2 cells were purchased from American Type Culture Collection (Manassas, VA, USA). The HK‐2 cells were cultured in Dulbecco's modified Eagle medium (DMEM)/F12 supplemented with 10% fetal bovine serum (FBS) and 1% penicillin‐streptomycin at 37 °C in a humidified atmosphere of 5% CO_2_. The siRNA for silencing the expression of ATG5, HIF‐1*α*, HSP90, as well as negative control siRNA, were produced by Genepharma Inc. (Shanghai, China). The sequences were recorded in Table S3 (Supporting Information). Briefly, the HK‐2 cells were seeded in 6‐well culture plates, when the cell confluence reached 60–80%, the siRNAs were transfected at 5 nM for 6 h using GP‐transfect‐Mate (GenePharma, Shanghai, China) in serum‐free OPTI‐MEM media. Thereafter, the medium on the cells was replaced with fresh medium alone or administrated with UA (800 µM, cat: 21604, Sigma–Aldrich, St Louis, MO), AA (5 µg mL^−1^, cat: F7876, Sigma–Aldrich) or TGF‐*β*1 (5 ng mL^−1^, cat:7754‐BH, R&D Systems) for an additional 36 h. On the other hand, to illustrate the mechanism of HSP90‐HIF‐1*α*‐mediated glycolysis, the HIF‐1*α* selective inhibitor KC7F2 (5 µM, cat: S7946, Selleckchem) and the glycolysis inhibitor 2‐DG (2 mM, cat: S4701, Selleckchem) were added 1 h before the stimulation with or without UA (800 µM) for 36 h. Then cells were harvested for subsequent RNA, protein and immunofluorescence studies, and the culture medium was collected and stored at −20 °C. To verify the proteasome degradation of HIF‐1*α*, HK‐2 cells were administrated with MG132 (5 µM, cat: S2619, Selleckchem) in the presence or absence of UA (800 µM), ATG5 siRNA or HSP90 siRNA for 4 h. To evaluate the half‐life of HIF‐1*α*, HK‐2 cells pretreatment with ATG5 siRNA or HSP90 siRNA were treated with CHX (10 µg mL^−1^, cat: HY‐12320, MedChemExpress, Shanghai, China) and harvested at the 0, 6, 12, 24 h followed by protein studies. To determine whether ATG5 mediates HIF‐1*α* via HSP90, it first transfected HK‐2 cells with HSP90 siRNA or ATG5 siRNA for 36 h, and then transfected the ATG5 pcDNA3.0 plasmid (The plasmid ATG5 pcDNA3.0 was generously provided by Dr. Mao's laboratory at Sun Yat‐Sen University, Guangzhou, China)^[^ ^7^ ^]^ or HSP90 pcDNA3.0 plasmid (MiaoLing Plasmid, Wuhan, China) for an additional 36 h using Lipofectamine LTX and Plus reagent (cat: 15338100, Invitrogen, USA) in Opti‐mem according to the manufacturer's instructions. To determine whether ATG5 regulates glycolysis in an autophagy‐dependent or autophagy‐independent manner, it transfected HK‐2 cells with a plasmid expressing a mutant form of ATG5 (ATG5‐K130R, which was generously provided by Dr. Mao's laboratory) using Lipofectamine LTX and Plus reagent or treatment with autophagy inhibitor 3‐MA (5mM, cat: S2767, Selleckchem) for 36 h. All of the in vitro experiments were repeated in triplicate.

### RNA‐Sequencing Analysis

Total RNA was isolated using the TRIzol reagent (Invitrogen, CA, USA), following the manufacturer's guidelines. RNA purity and measurement were executed using the NanoDrop 2000 Spectrophotometer (Thermo Scientific, USA). RNA integrity was examined using the Agilent 2100 Bioanalyzer (Agilent Technologies, Santa Clara, CA, USA). Subsequently, library construction ensued using the VAHTS Universal V6 RNA‐seq Library Prep Kit, adhering to the manufacturer's protocols. These libraries were subjected to sequencing on an Illumina NovaSeq 6000 platform, generating 150 bp paired‐end reads. After sequencing, alignment of clean reads to the reference genome was done with HISAT2, and calculation of FPKM values for each gene took place; raw count data were derived for individual genes with HTSeq‐count. Differential expression analysis was conducted with DESeq2, where a gene was deemed significantly differentially expressed (DEG) if it had a q value <0.05 and a fold change either greater than 2 or less than 0.5. To visualize distinct gene expression patterns, hierarchical clustering analysis was performed on the DEGs in R version 3.2.0. Additionally, a radar chart was plotted for the top 30 DEGs utilizing the R package “ggradar”, thus illustrating the upregulated and downregulated gene expressions. The transcriptome sequencing and analysis were conducted by OE Biotech Co., Ltd. (Shanghai, China).

### Human Renal Specimens

It collected the human renal specimens at the Shanghai East Hospital affiliated to Tongji University School of Medicine. Totally, it obtained 20 renal biopsy samples from CKD patients diagnosed with IgA nephropathy, and 11 kidney specimens from patients who underwent nephrectomy due to renal carcinoma but without any other concurrent kidney diseases as control. According to the electronic medical records from Shanghai East Hospital, the data on the characteristics of patients were collected retrospectively. The images about IgA‐fluorescence staining and electron microscope were also obtained from the pathology reporting system of Shanghai East Hospital. Written informed consent was obtained from all patients. This research protocol complied with the ethical guidelines of the Declaration of Helsinki Principles and was approved by Research Ethics Committee of Shanghai East Hospital (No.2023082).

### Histological Analysis

Renal tissues were fixed in 4% paraformaldehyde overnight, then, paraffin embedded and 4 µm tissue sections were used for histology examination. To evaluate the morphology and tubular injury, PAS staining was performed according to the manufacture's protocols. The degree of tubular injury was determined using a semiquantitative grade scale according to the following system: 0 = normal, 1 = injury<30%, 2 = injury, 30–60%, and 3 = injury>60%. To assess the kidney fibrosis, Masson's trichrome staining and Sirius red staining were performed according to manufactures instructions. Ten randomly selected fields per section were photographed with Leica microscope (Leica, DM3000) at 20× magnification. Photographs were analyzed blindly to ImageJ software (National Institutes of Health, Bethesda, MD, USA).

### Hematoxylin and Eosin Staining

Tissues from heart, liver, spleen, lung and intestine were transferred to 4% paraformaldehyde and fixed overnight, then, paraffin embedded and 4 µm tissue sections were prepared and subjected to Hematoxylin and eosin (H&E) staining by standard protocol. Briefly, the sections were first deparaffinized and rehydrated through a graded ethanol series, following incubation in hematoxylin solution for 15 min and subsequently in eosin solution for 1 min. Images were acquired using a Leica Microscope (Leica, DM3000) at 20× magnification.

### Immunohistochemical Staining

Formalin‐fixed, paraffin‐embedded renal tissue sections were subjected to immunohistochemical staining. The sections were deparaffinized, rehydrated, and heated in citrate buffer for antigen retrieval. Subsequently, the sections were incubated with 3% H_2_O_2_ to block endogenous peroxidase activity and then 10% normal goat serum to reduce nonspecific binding. Thereafter, the sections were incubated with primary antibodies at 4 °C overnight and rinsed in phosphate buffered saline (PBS) three times followed by incubation with a horseradish peroxidase (HRP) polymer horse‐anti‐rabbit secondary antibody at room temperature for 1 h. Antibodies used in immunohistochemistry are summarized in Table S2 (Supporting Information). Afterwards, the sections were washed and stained with DAB chromogen. Finally, the sections were dehydrated with alcohol gradient and sealed with neutral glue. Images were collected using Leica Microscope (Leica, DM3000) at 20× magnification. Ten randomly selected fields per section were photographed and the percentage of stained positive areas was quantified by ImageJ software.

### Transmission Electron Microscopy

Kidney tissues and HK‐2 cells were initially fixed using a 2.5% glutaraldehyde solution in a sodium bicarbonate buffer. They were subsequently embedded in a 2% agarose matrix and further fixed with a 1% buffered osmium tetroxide solution. The specimens were then counterstained with 2% uranyl acetate and ethanol. Following a systematic dehydration process, they were infiltrated and embedded in ‐812 resin. Thin sections were cut from the ultrasections and finally stained using a combination of 2% uranyl acetate and lead citrate solutions. Electron photomicrographs were taken of ultrastructures of autophagosome and mitochondria. A mitochondrion was classified as damage if it was 5–6 times the size of a normal mitochondrion or displayed signs of membrane rupture.^[^ ^56^ ^]^ The mitochondrial number and length were determined using ImageJ software from individual mitochondria.^[^ ^57^ ^]^

### Immunofluorescence Staining

Immunofluorescence staining of the kidney was performed on 4 µm paraformaldehyde‐fixed, paraffin‐embedded tissue sections. The kidney tissue sections were deparaffinized, rehydrated and exposed to primary antibodies staining at 4 °C overnight. After washing, the sections were incubated with corresponding Alexa Fluor (cat: 715‐545‐151, Jackson ImmunoResearch, PA, USA) or Cy3 (cat: 711‐165‐152, Jackson ImmunoResearch) secondary antibodies for 1 h at room temperature. Cell nuclei were incubated with 4′‐6‐Diamidino‐2‐phenylindole (DAPI) for 10 min. Subsequently, the sections were mounted with antifade reagent and observed under a fluorescence microscope (Leica, DM3000). Ten random fields (20×) were selected from each section and the positive staining areas were quantified by ImageJ software.

HK‐2 cells were fixed with 4% formaldehyde for 15 min and permeabilized using 0.25% Triton X‐100. Cells were blocked by 10% goat serum for 30 min at room temperature and incubated with the indicated primary antibodies at 4 °C overnight, then incubated with fluorescent secondary antibodies for 1 h at room temperature, and mounted with DAPI. The slides were examined by fluorescence (Leica, DM3000). Ten random fields (20×) were selected from per slides and the positive staining cells were quantified by ImageJ software. Antibodies used in immunofluorescence staining are summarized in Table S2 (Supporting Information).

### Generation of Tubule‐Specific ATG5 Knockout Mice

All experimental protocols for animal studies were reviewed and approved by the Institutional Animal Care and Use Committee at Tongji University (Document No. TJBB00623107). ATG5^fl^/^+^ mice (exon 3 flanked by loxP sites, cat: NM‐CKO‐00131) were ordered from Shanghai Model Organisms Center Inc. (Shanghai, China). Cadherin 16‐Cre mice (Tg(Cdh16‐Cre)91Igr/J, Stock No: 012237) were ordered from the Jackson Laboratory (Bar Harbor, ME, USA). To generated the tubule‐specific ATG5 knockout mice, ATG5^fl^/^+^ mice were crossed with Cdh16‐Cre mice to generate offsprings with tubular cell deletion of ATG5 (Cdh16‐Cre^+^:ATG5^fl/fl^, cKO). Littermate mice genotyping with Cdh16‐Cre^−^: ATG5^fl/fl^ were considered as wild‐type (WT) controls. All animals were maintained in the Specific Pathogen Free (SPF) conditions (12 h light/dark cycle, 24 °C and 40–60% humidity) with ad libitum access to water and standard laboratory chow diet.

### Animal Models

For HN, AAN and uIRI mice models, 8‐week‐old male ATG5‐WT mice and ATG5‐cKO mice weighing ≈23–25 g were used. The HN mice model was established by oral administration of a mixture of adenine (0.14 g kg^−1^, cat: 65000134, Sinopharm Chemical Reagent Co., Lit. Shanghai, China) and potassium oxonate (2.1 g kg^−1^, cat: 2207‐75‐2, Macklin, Shanghai, China) once a day for 21 consecutive days. Mice in the sham group were given an equivalent amount of saline by gavage. For the AAN model, mice were subjected to daily intraperitoneal injections of aristolochic acid (2 mg kg^−1^) for 21 days, while those in the sham group received equivalent volumes of saline injections.^[^ ^58^ ^]^ For uIRI model, the left renal pedicle of the mouse was clamped for 45 min and then released.^[^ ^59^ ^]^ The exact number of mice employed in each group was detailed in the figure legends respectively. All mice were anesthetized and sacrificed to collect tissues and blood after 21 days.

To investigate the role of glycolysis on mitochondrial fission and inflammation in kidney fibrosis, it also established a HN mice model administration with 2‐DG. Briefly, male C57BL/6 mice weighing ≈23–25 g were housed in the SPF conditions (12 h light/dark cycle, 24 °C and 40–60% humidity) with ad libitum access to water and standard laboratory chow diet. Mice were randomly divided into four groups, including sham, sham+2‐DG, HN, and HN+2‐DG groups. The HN model was induced by oral administration of a mixture of adenine (0.14 g kg^−1^) and potassium oxonate (2.1 g kg^−1^) once a day for 21 consecutive days. Meanwhile, mice from sham+2‐DG and HN+2‐DG groups were intraperitoneal injected with 2‐DG (80 mg kg^−1^) once a day for 21 days. All mice were anesthetized and euthanized to collect kidney tissue and blood after 21 days.

### Serum Creatinine, BUN, Serum Uric Acid, Lactate and Glucose Consumption Measurements

Blood samples were collected and subsequently subjected to centrifugation processes. The serum creatinine was determined using a creatinine detection kit (cat: C011‐2‐1, Nanjing Jiancheng Bioengineering Institute, Nanjing, China). The BUN was determined using a BUN detection kit (cat: C013‐2‐1, Nanjing Jiancheng Bioengineering Institute). The uric acid was determined using a uric acid detection kit (cat: C012‐2‐1, Nanjing Jiancheng Bioengineering Institute). The lactate concentration of serum and culture medium was determined using a lactate assay kit (cat: A019‑2‑1, Nanjing Jiancheng Bioengineering Institute). The glucose concentration of culture medium was determined using glucose assay kit (cat: A154‐1‐1, Nanjing Jiancheng Bioengineering Institute). All detections were strictly carried out in accordance with the manufacturers procedures.

### Immunoblotting and Immunoprecipitation

Kidney tissue and HK‐2 cells were lysed to obtain proteins using radioimmunoprecipitation assay (RIPA) buffer according to the manufacturer's protocols, and protein concentration was measured by bicinchoninic acid (BCA) kit. Then proteins were denatured at 98 °C for 20 min. Equivalent protein of each group was separated on 8–12% sodium dodecyl sulfate‐polyacrylamide gel electrophoresis (SDS‐PAGE) gels and transferred onto polyvinylidene fluoride (PVDF) membrane. After blocking with 5% bovine serum albumin (BSA), transferred membranes were incubated with specific primary antibodies at 4 °C overnight. After washing the membrane for three times, the membrane was incubated with corresponding secondary antibodies at room temperature for 1 h, and detected by a chemiluminescence reagent system using ECL kit. Densitometry analyses of immunoblots were conducted by ImageJ software.

For immunoprecipitation, HK‐2 cells grown in 10 cm dishes were washed with PBS, then lysed on ice for 30 min in 1 mL cold low‐stringency lysis buffer. Lysates were centrifuged at 12000 g for 10 min at 4 °C. The supernatant was collected and incubated with the targeted antibodies or IgG, followed by addition of 20 µL A/G PLUS‐Agarose beads (SC‐2003, Santa Cruz Biotechnology, Santa Cruz, CA, USA) with rotation at 4 °C overnight. Subsequently, the beads were washed five times with a 1 mL low‐stringency lysis buffer between each wash at 2500 g for 5 min at 4 °C. The wash buffer was discarded, and 50 µL of 1× SDS‐PAGE loading buffer was added to the immune complexes, which were then denatured at 100 °C for 10 min. Subsequently, the samples were processed for western blot analysis as described above. Antibodies used in immunoblotting and immunoprecipitation are summarized in Table S2 (Supporting Information).

### Quantitative Real‐Time Polymerase Chain Reaction (RT‐qPCR)

Total RNA was extracted from HK‐2 cells with Trizol reagent (Invitrogen, CA, USA) as recommended by the manufacturer. For cDNA synthesis, 1 µg of the total mRNA was reverse transcribed into cDNA in a 20 µL reaction volume by using ABScript III RT Master Mix (cat: RK20429, ABclonal, Wuhan, China). RT‐qPCR was performed using EvaGreen 2× qPCR MasterMix (cat: G891, Applied Biological Materials, Richmond, BC, Canada) on a 7500 Real‐Time PCR System (Applied Biosystems, Foster City, CA, United States). PCR primers sequences used for amplification of genes were recorded in Table S4 (Supporting Information). The relative gene expression was quantified based on the 2^−ΔΔCT^ method and normalized with *β*‐actin. Assays were repeated in triplicate.

### Seahorse Assays

Metabolic analysis was conducted by using a Seahorse XF Glycolytic Rate Assay Kit (cat: 103344‐100, Seahorse Bioscience, North Billerica, MA, USA). Briefly, after treatments as described above, HK‐2 cells were detached and seeded in XFe96 cell culture plates at a concentration of 10000 cells per well, then cultured in the incubator at 37 °C and 5% CO_2_ overnight. On the following day, the medium was replaced with the assay medium, Seahorse XF DMEM (pH 7.4), which was free from phenol red and supplemented with 1 mM sodium pyruvate, 10 mM glucose, and 2 mM glutamine and incubated for 1 h in a CO_2_‐free incubator. Cells were sequentially treated with the following inhibitors: Rotenone/antimycin A (Rot/AA), which were agents that block mitochondrial oxygen consumption and consequently reduce the release of protons resulting from CO_2_ breakdown. Subsequently, 2‐DG was administered, an inhibitor that competitively binds to glucose hexokinase, the first enzyme in the glycolysis pathway. All parameters were automatically calculated and recorded by the Seahorse XF Glycolytic Rate Assay Report Generator and Wave software (version 2.6.3).

### JC‐1 Staining

The mitochondrial membrane potential was measured by JC‐1 staining kit (cat: C2003, Beyotime Biotechnology, Shanghai, China) according to the manufacturer's instructions. First, JC‐1 working solution was prepared. The HK‐2 cells were washed with PBS and then stained with JC‐1 working solution at 37 °C for 20 min. Subsequently, the dye solution was removed and the cells were washed with JC‐1 staining buffer and observed with a fluorescence microscope (Vert A1, Zeiss). Changes in mitochondrial membrane potential were evaluated by the ratio of red fluorescence (aggregated JC‐1) to green fluorescence (monomeric JC‐1).

### Statistical Analysis

Statistical analysis was performed using GraphPad Prism (version 8.0.2) and IBM SPSS Statistics 20.0 software, and data plotting was conducted using GraphPad Prism (version 8.0.2). Data depicted in graphs were expressed as mean ± SEM. The statistical data presented were originated from no less than three biologically independent experimental replicates. The sample size (n) was indicated in the figure legends. Comparisons between two groups were conducted using the unpaired two‐tailed Student's t‐test, while one‐way analysis of variance (ANOVA) followed by Tukey's test was used for comparisons among multiple groups. Categorical variables were analyzed using the chi‐squared (χ^2^) test. Spearman correlation analysis was used to evaluate the coefficient (r) and *P* value. *P*<0.05 was considered statistically significant. In the figures, asterisks refer to significance levels as *p <0.05, **p <0.01, ***p <0.001, ****p <0.0001, and N.S. denote statistically not significant.

## Conflict of Interest

The authors declare no conflict of interest.

## Author Contributions

N.L. participated in research design. Y.H., J.L., H.C., Y.S., X.M., Y.W., X.L., Q.Z., Y.W., and D.J. conducted experiments. Y.H. contributed new reagents or analytic tools. Y.H. performed data analysis. Y.H., S.Z., and N.L. wrote or contributed to the writing of the manuscript. All authors have read and approved the final manuscript.

## Supporting information



Supporting Information

## Data Availability

The data that support the findings of this study are available from the corresponding author upon reasonable request.
