# Supplementary material for: Autophagy Related 5 Promotes Mitochondrial Fission and Inflammation via HSP90‐HIF‐1α‐Mediated Glycolysis in Kidney Fibrosis
Source: Adv Sci (Weinh). 2025 Mar 6;12(17):2414673. doi: 10.1002/advs.202414673 (PMC12061336; doi:10.1002/advs.202414673)
Supplement: Supplementary file 1 — Supporting Information [file ADVS-12-2414673-s001.docx]

**Supporting Information**

**Autophagy Related 5 Promotes Mitochondrial Fission and Inflammation via HSP90-HIF-1α-Mediated Glycolysis in Kidney Fibrosis**

*Yan Hu, Jinqing Li, Hui Chen, Yingfeng Shi, Xiaoyan Ma, Yi Wang, Xialin Li, Qin Zhong, Yishu Wang, Daofang Jiang, Shougang Zhuang, Na Liu**


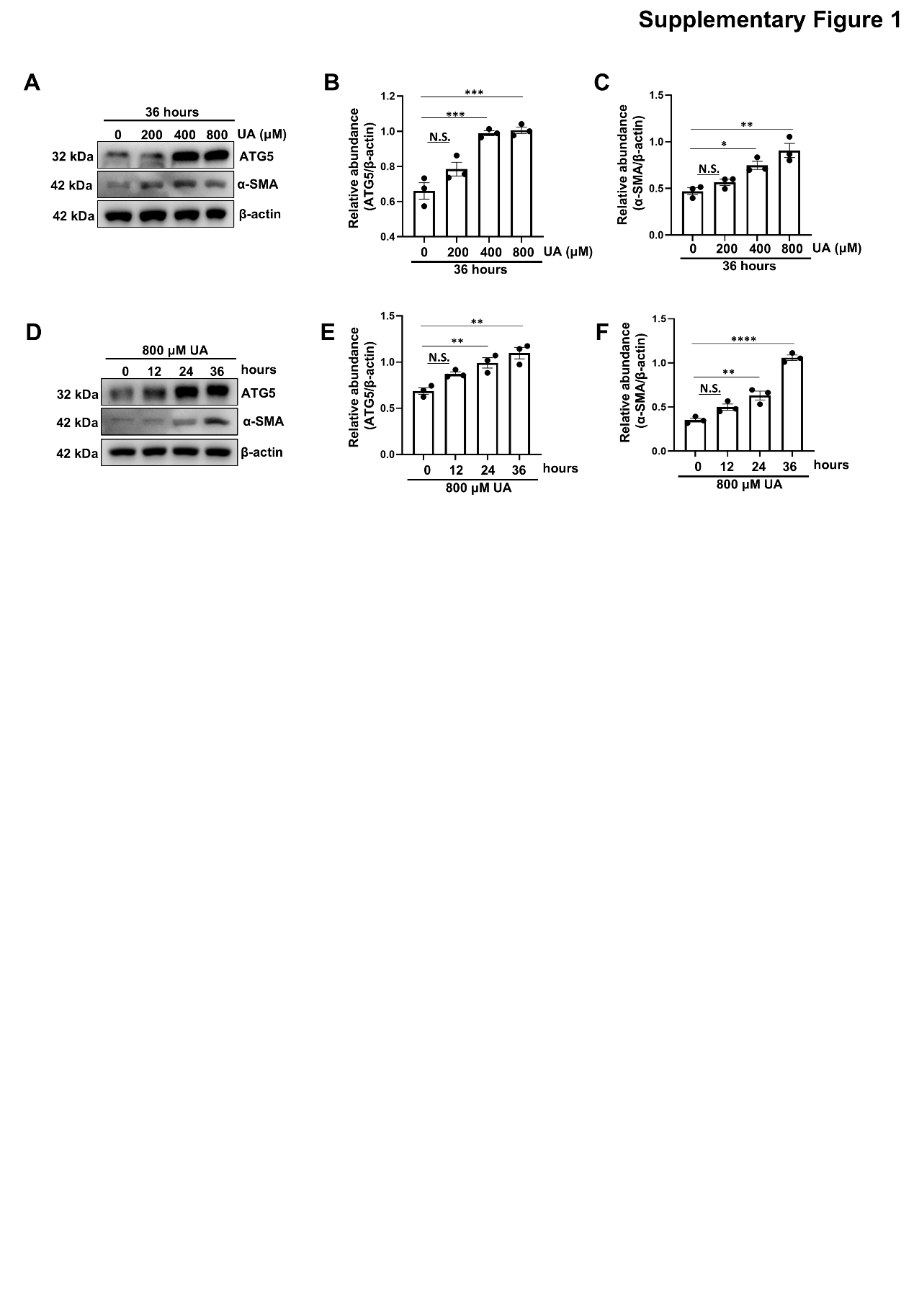


**Supplementary Figure 1. ATG5 is highly upregulated UA-stimulated HK-2 cells in a dose-dependent manner and time-dependent manner. A)** HK-2 cells were stimulated with different doses of UA (0 μM, 200 μM, 400 μM, 800 μM) for 36 hours. Representative western blot images show the relative protein levels of ATG5 and α-SMA. **B, C)** Quantitative analyses of ATG5 and α-SMA. **D)** HK-2 cells were stimulated for different periods of time (0 h, 12 h, 24 h, 36 h) at a concentration of 800 μM. Representative western blot images show the relative protein levels of ATG5 and α-SMA. **E, F)** Quantitative analyses of ATG5 and α-SMA. n=3 per group. Data are expressed as mean ± SEM. **P*<0.05, ***P*<0.01, ****P*<0.001, *****P*<0.0001, and N.S. denote statistically not significant.


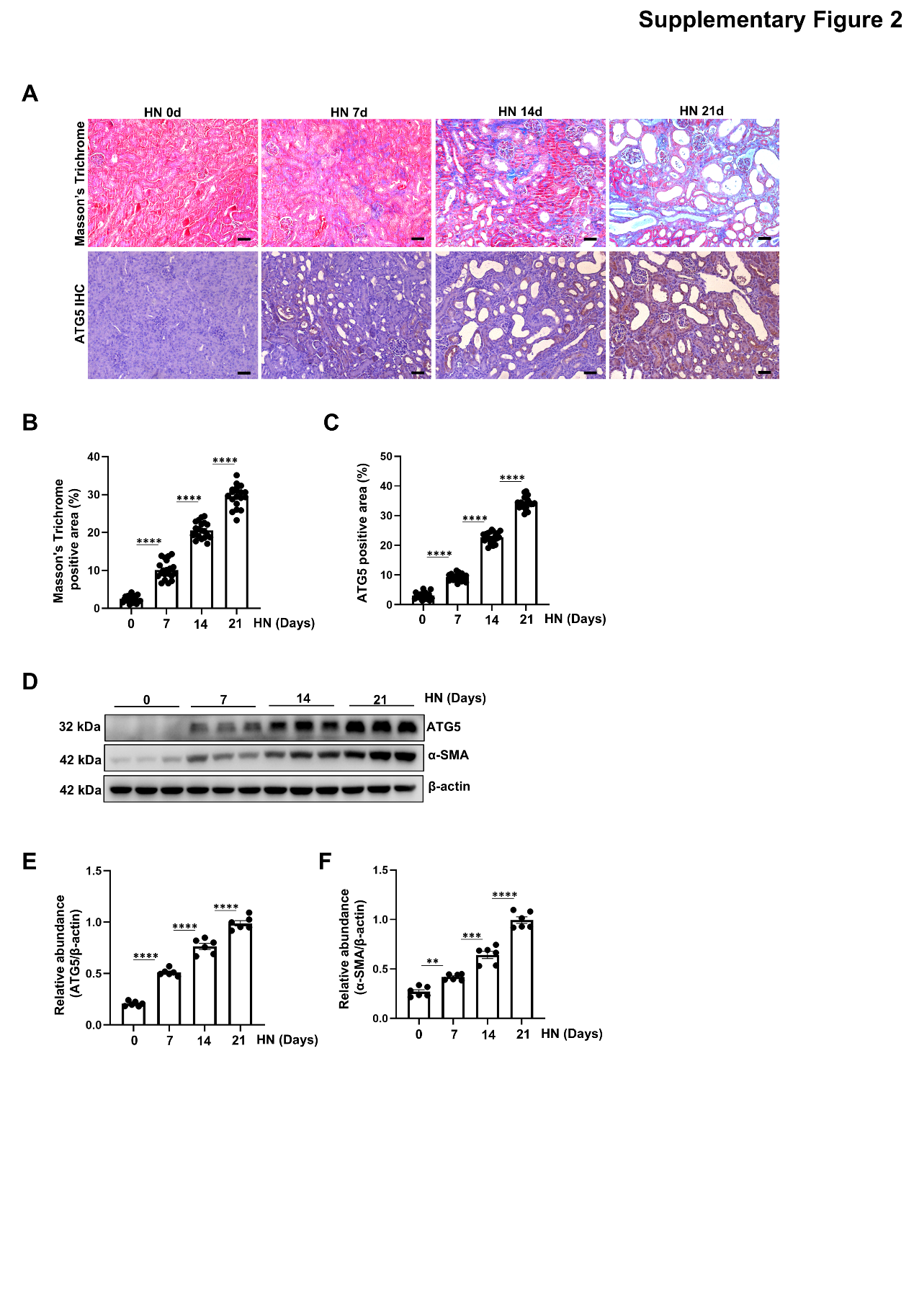


**Supplementary Figure 2. ATG5 is increased in HN mouse model in a time-dependent manner. A)** Representative images of Masson’s trichrome staining and ATG5 immunohistochemical staining in kidneys from different groups of mice. Scale bar = 50 µm. **B)** Quantitative analysis of Masson’s trichrome positive area. **C)** Quantitative analysis of ATG5 positive area. **D)** Western blot for ATG5 and α-SMA in kidneys from different groups of mice. **E, F)** Quantitative analyses of ATG5 and α-SMA in kidneys from different groups of mice. n=6 per group. Data are expressed as mean ± SEM. ***P*<0.01, ****P*<0.001, *****P*<0.0001.


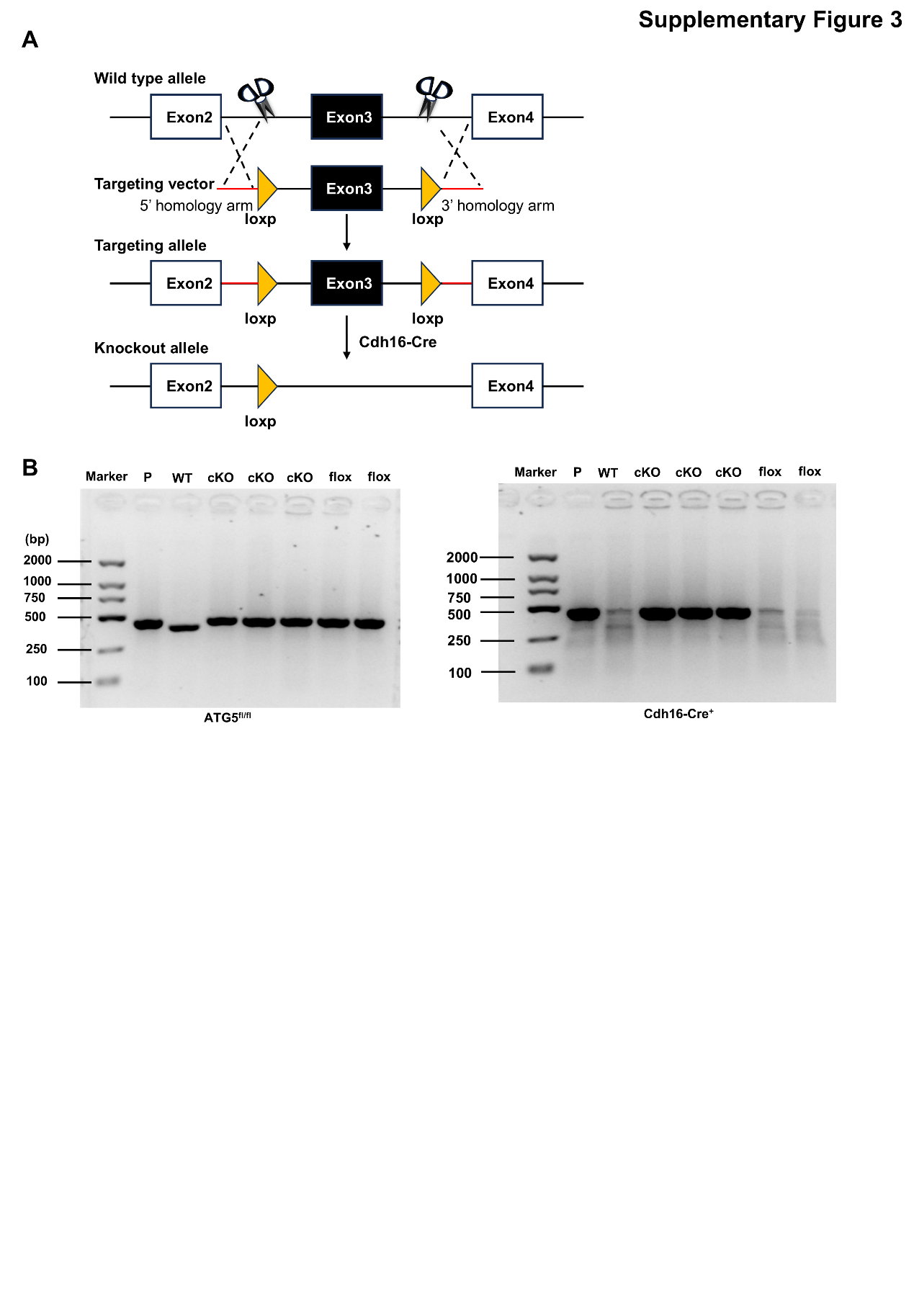


**Supplementary Figure 3. Establishment of tubule-specific ATG5 knockout (Cdh16-Cre^+^: ATG5^fl/fl^) mice. A)** Experimental scheme for generating of tubule-specific ATG5 knockout mice. Exon 3 is deleted upon Cdh16-Cre mediated recombination. **B)** Genotyping of tubule-specific ATG5 knockout mice was performed.


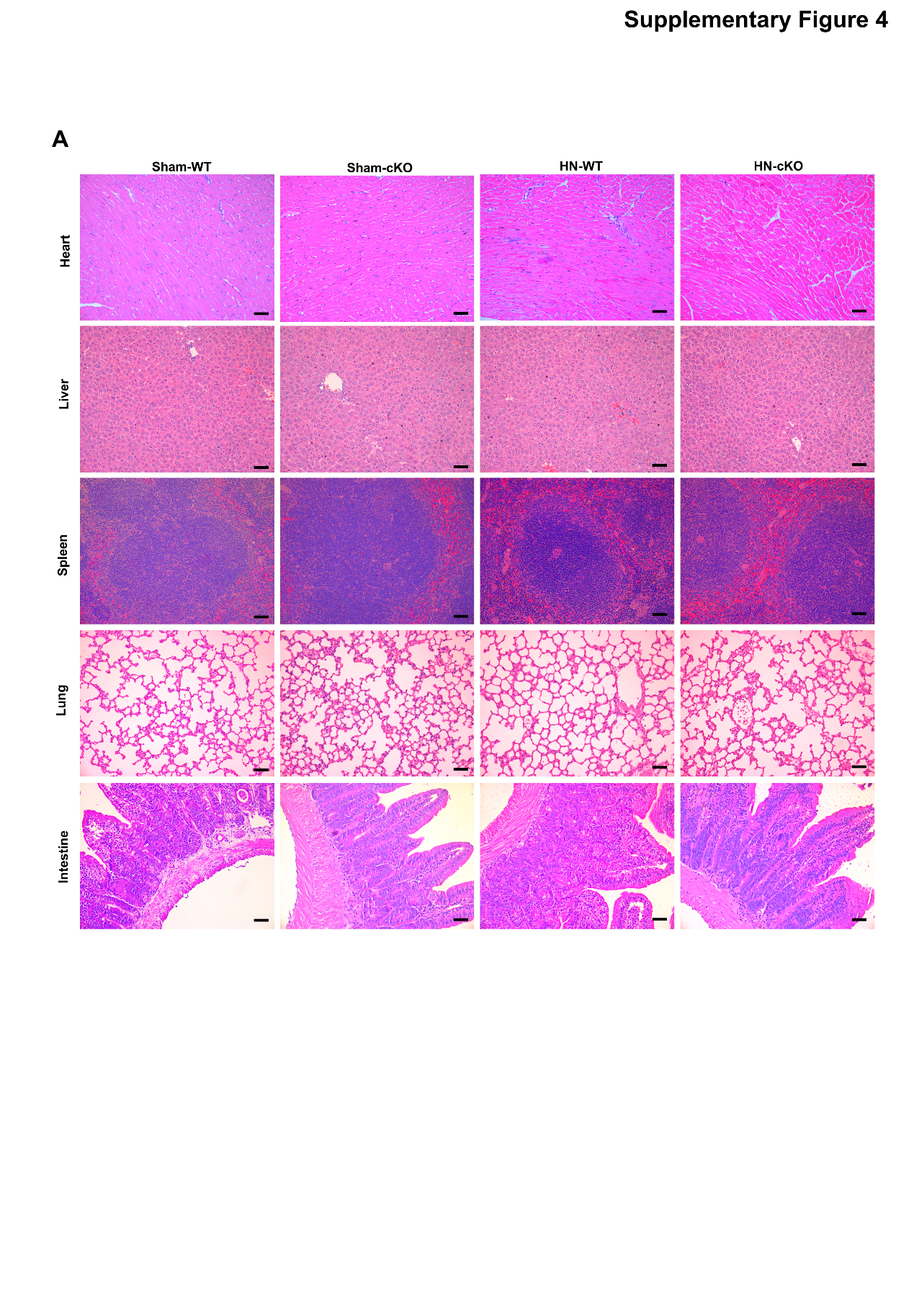


**Supplementary Figure 4. The tubule-specific ATG5 knockout mice have no obvious structural changes in other organs. A)** Representative photomicrographs of H&E staining in heart, liver, spleen, lung and intestine from different groups of mice. Scale bar = 50 µm.


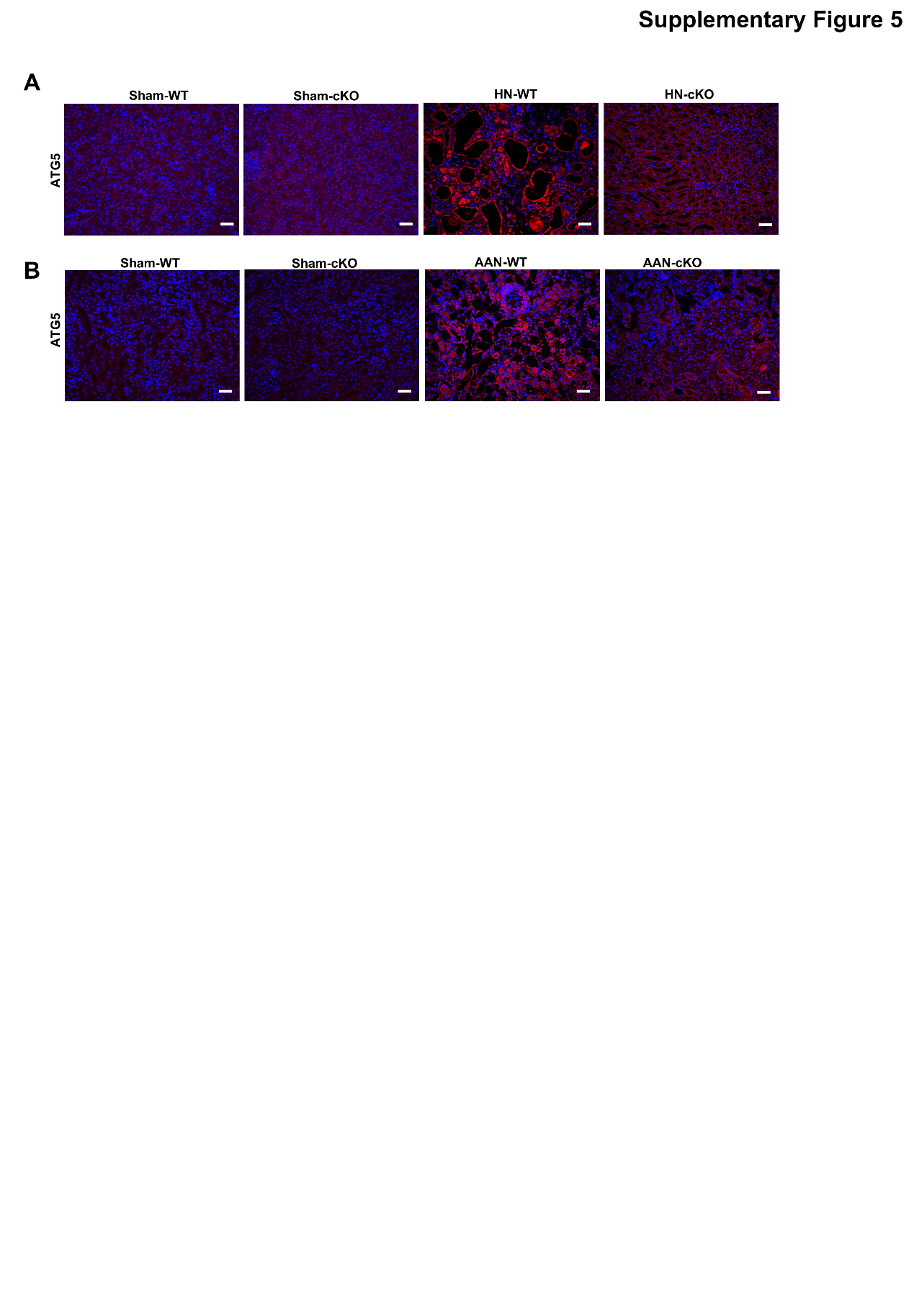


**Supplementary Figure 5. Tubule-specific ATG5 deletion reduced the expression of ATG5. A)** Representative photomicrographs of immunofluorescence staining with ATG5 in HN mice model. Scale bar = 50 µm. **B)** Representative photomicrographs of immunofluorescence staining with ATG5 in AAN mice model. Scale bar = 50 µm.


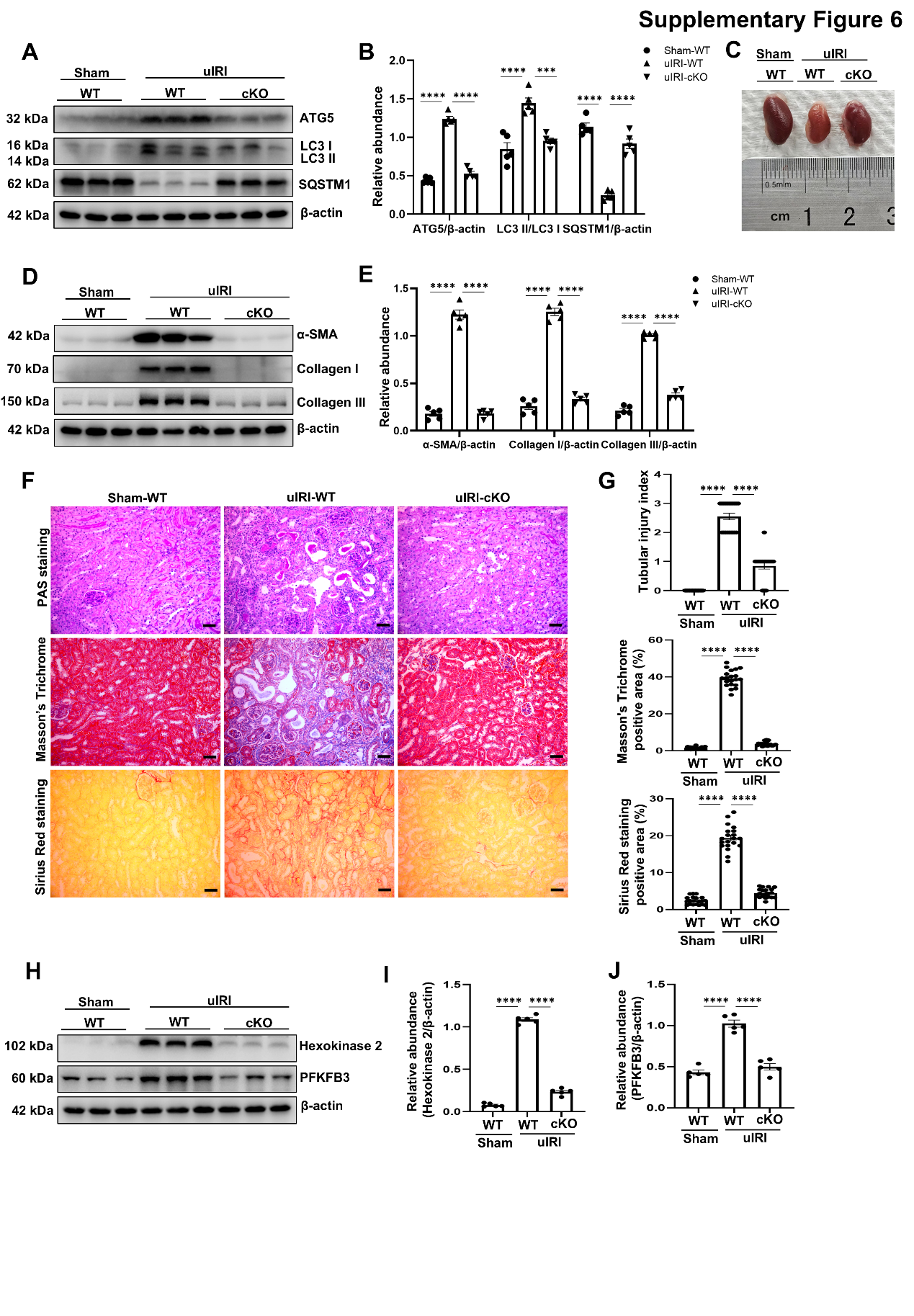


**Supplementary Figure 6. Tubule-specific ATG5 ablation inhibits kidney fibrosis and ameliorates glycolysis in uIRI mice model****. A)** Western blot for ATG5, LC3 and SQSTM1 in kidneys from different groups of mice. **B)** Quantitative analyses of ATG5, LC3 II/I and SQSTM1 in kidneys from different groups of mice. **C)** Photomicrographs showing the size, color and texture in kidneys from different groups of mice. **D)** Western blot for α-SMA, collagen I, and collagen III in kidneys from different groups of mice. **E)** Quantitative analyses of α-SMA, collagen I, and collagen III standardized to β-actin in kidneys from different groups of mice. **F, G)** Representative images of PAS staining, Masson’s trichrome and Sirius red staining in kidneys from different groups of mice. Tubular injury index, Masson’s trichrome positive area and Sirius red staining positive area among groups as indicated. Scale bar = 50 µm. **H)** Western blot for hexokinase 2 and PFKFB3 in kidneys from different groups of mice. **I, J)** Quantitative analyses of hexokinase 2 and PFKFB3 standardized to β-actin in kidneys from different groups of mice. n=5 per group. Data are expressed as mean ± SEM. ****P*<0.001, *****P*<0.0001.


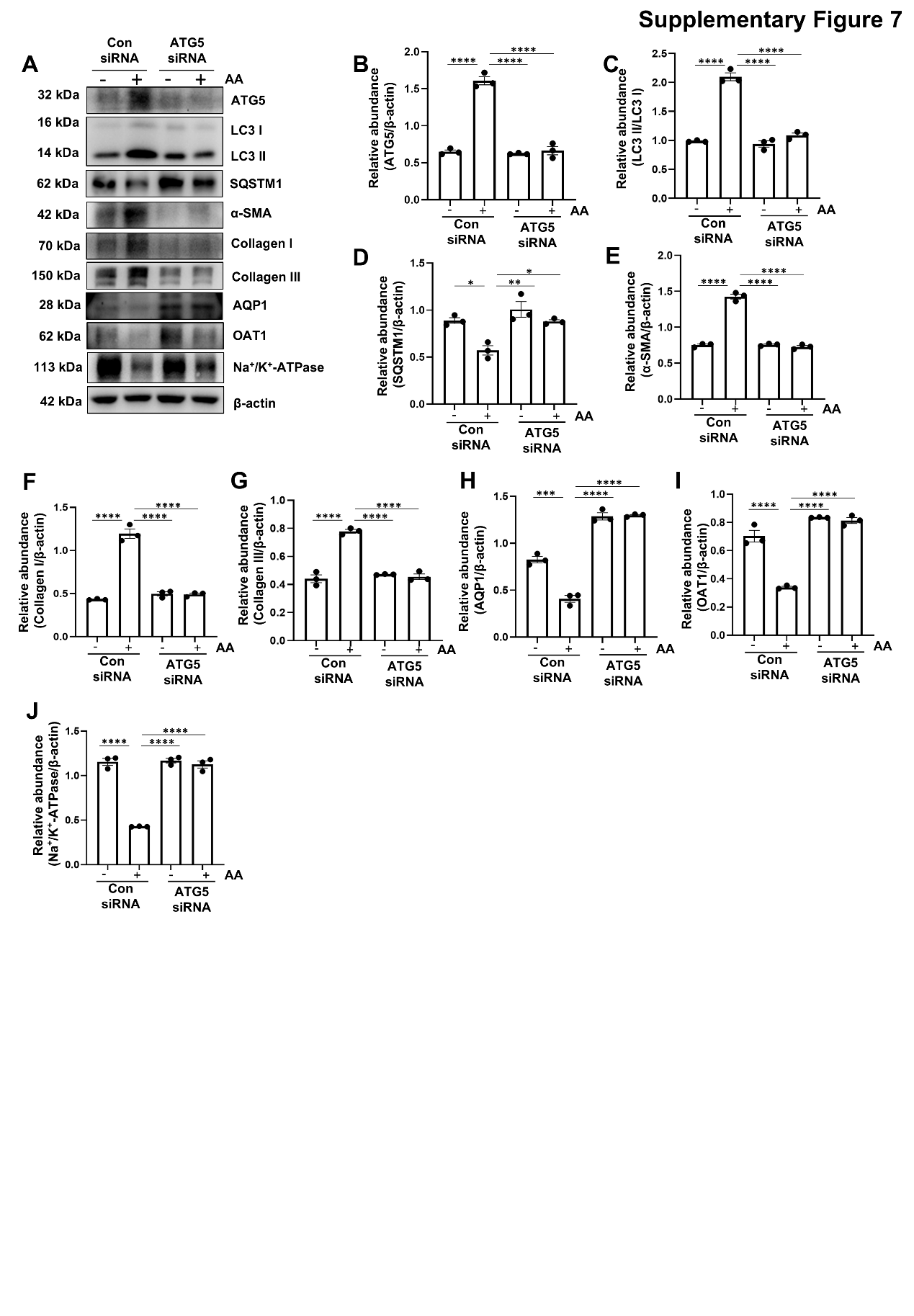


**Supplementary Figure 7. Gene silence of ATG5 inhibits fibrosis-related proteins accumulation in HK-2 cells induced by AA**. **A)** HK-2 cells were transfected with ATG5 siRNA or control siRNA and then incubated with or without AA (5 μg/mL) for an additional 36 hours. Representative western blot images showing the relative protein levels of ATG5, LC3, SQSTM1, α-SMA, collagen I, collagen III, AQP1, OAT1, and Na^+^/K^+^-ATPase. **B-J)** Quantitative analyses of ATG5, LC3 II/I, SQSTM1, α-SMA, collagen I, collagen III, AQP1, OAT1, and Na^+^/K^+^-ATPase. n=3 per group. Data are expressed as mean ± SEM. **P*<0.05, ***P*<0.01, ****P*<0.001, *****P*<0.0001.


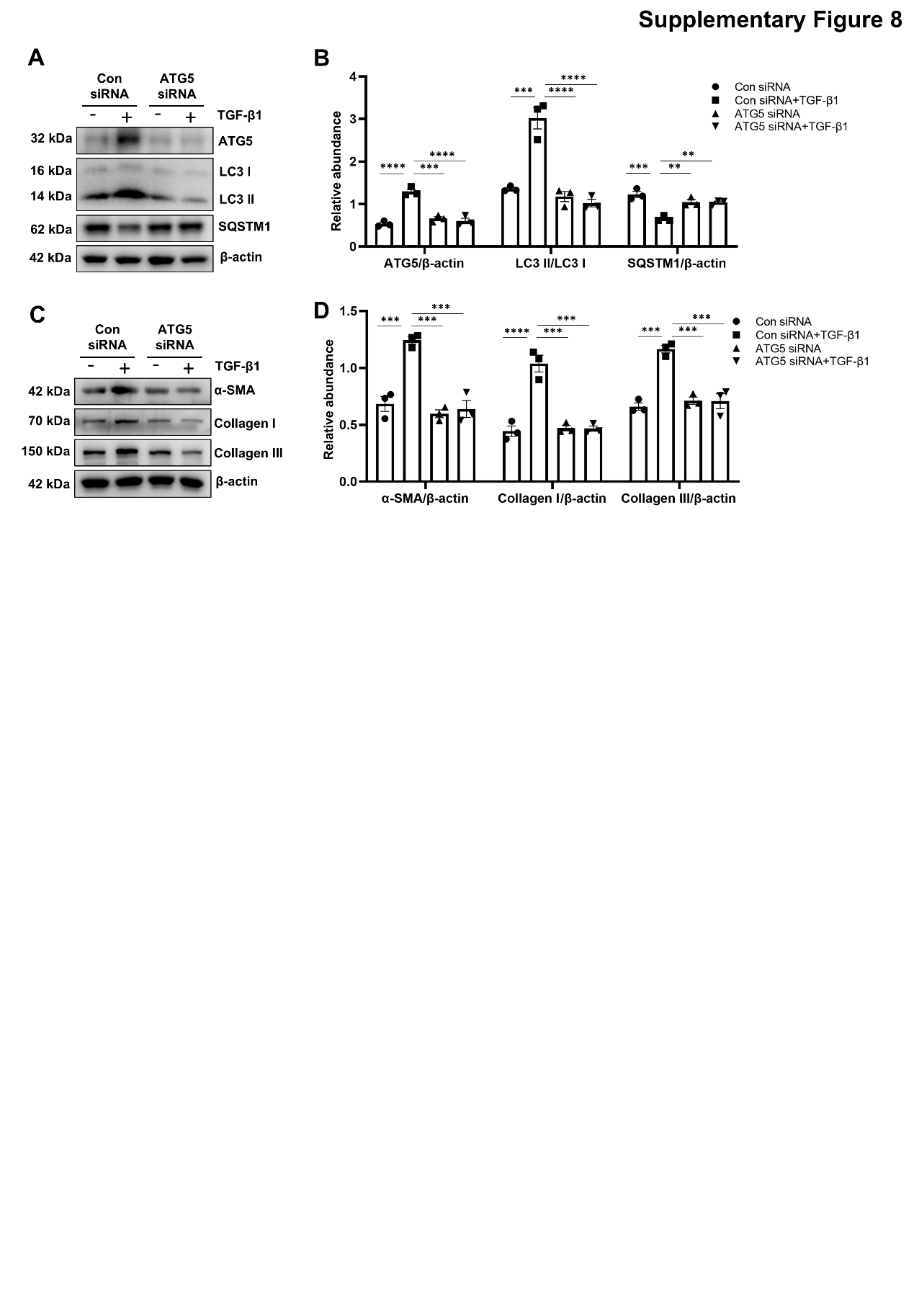


**Supplementary Figure 8. Gene silence of ATG5 inhibits fibrosis-related proteins accumulation in HK-2 cells induced by** **TGF-β1**. **A)** HK-2 cells were transfected with ATG5 siRNA or control siRNA and then incubated with or without TGF-β1 (5 ng/mL) for an additional 36 hours. Representative western blot images showing the relative protein levels of ATG5, LC3, and SQSTM1. **B)** Quantitative analyses of ATG5, LC3 II/I, and SQSTM1. **C)** Representative western blot images showing the relative protein levels of α-SMA, collagen I, and collagen III in HK-2 cells treated as in (**A**). **D)** Quantitative analyses of α-SMA, collagen I, and collagen III. n=3 per group. Data are expressed as mean ± SEM. ***P*<0.01, ****P*<0.001, *****P*<0.0001.


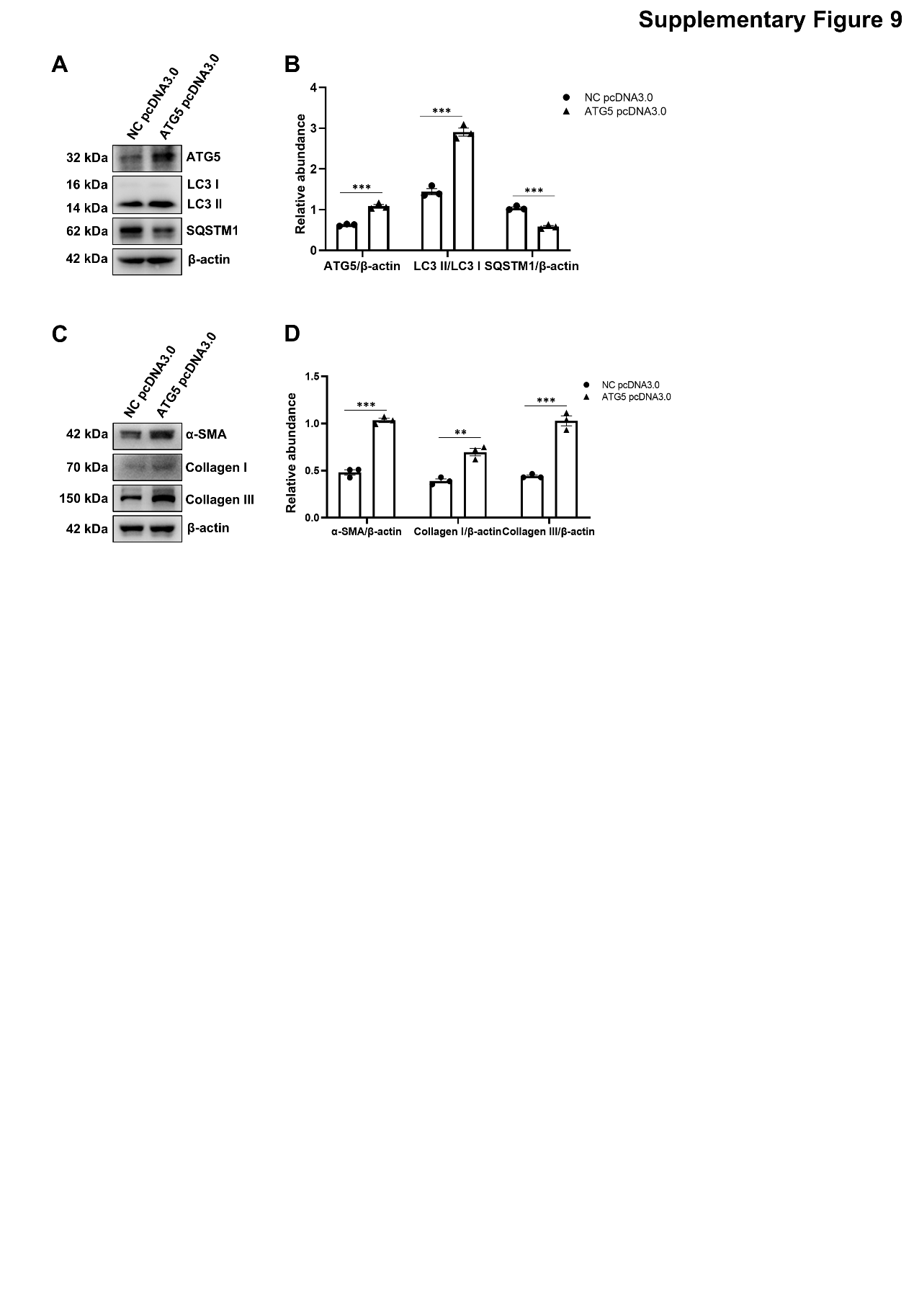


**Supplementary Figure 9. Overexpression of ATG5 promotes fibrosis-related proteins upregulation in HK-2 cells.** **A**) HK-2 cells were transfected with NC pcDNA3.0 and ATG5 pcDNA3.0. Representative western blot images showing the relative protein levels of ATG5, LC3, and SQSTM1. **B)** Quantitative analyses of ATG5, LC3 II/I, and SQSTM1. **C)** Representative western blot images showing the relative protein levels of α-SMA, collagen I, and collagen III in HK-2 cells treated as in (**A**). **D)** Quantitative analyses of α-SMA, collagen I, and collagen III. n=3 per group. Data are expressed as mean ± SEM. ***P*<0.01, ****P*<0.001.


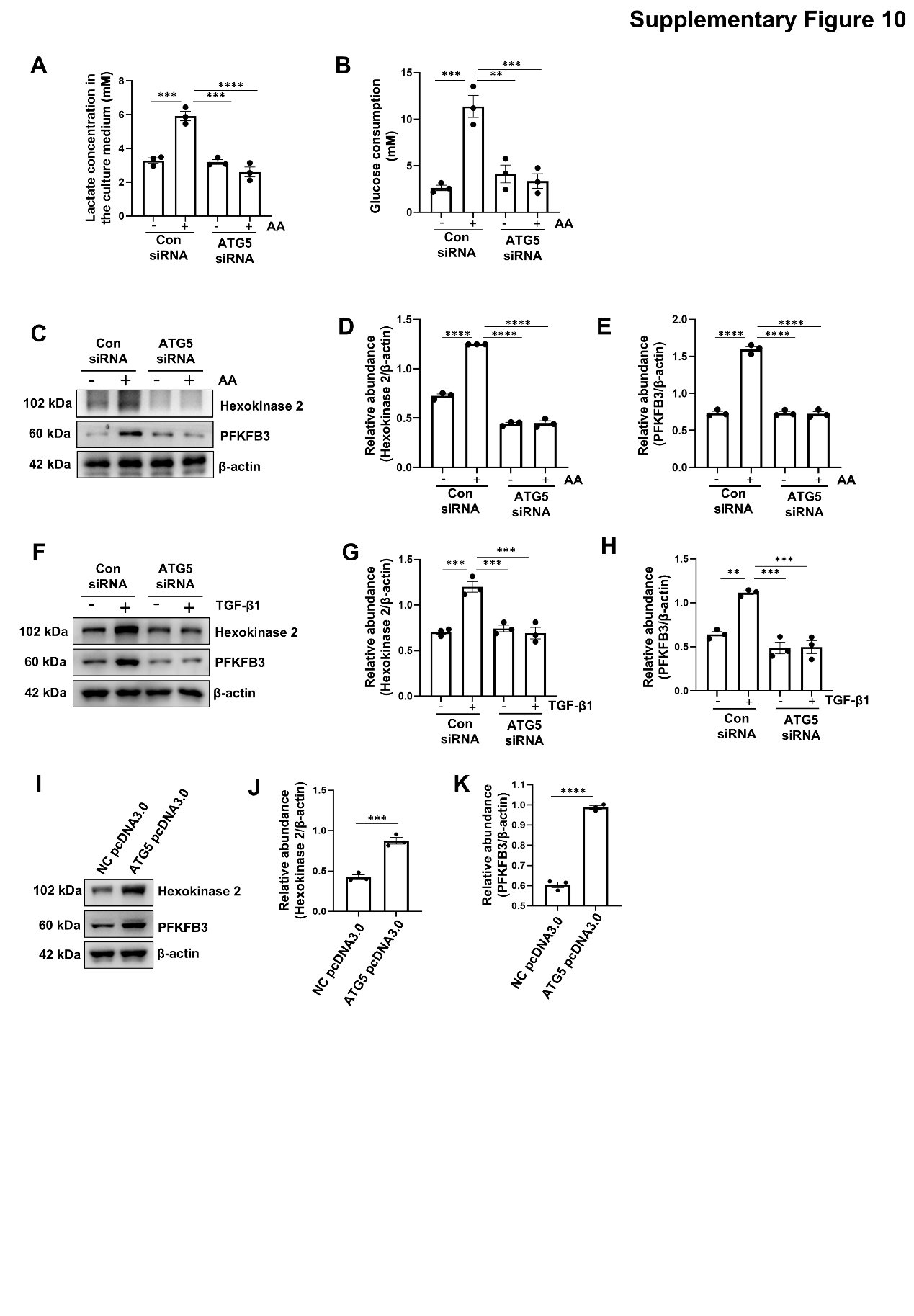


**Supplementary Figure 10. Gene silence of ATG5 attenuates glycolysis in HK-2 cells induced by AA and TGF-β1**. **A)** HK-2 cells were transfected with ATG5 siRNA or control siRNA and then incubated with or without AA (5 μg/mL) for an additional 36 hours. Amount of lactate in culture medium of HK-2 cells. **B)** Glucose consumption by HK-2 cells treated as in (**A**). **C)** Representative western blot images showing the relative protein levels of hexokinase 2 and PFKFB3 in HK-2 cells treated as in (**A**). **D, E)** Quantitative analyses of hexokinase 2 and PFKFB3 standardized to β-actin. **F)** HK-2 cells were transfected with ATG5 siRNA or control siRNA and then incubated with or without TGF-β1 (5 ng/mL) for an additional 36 hours. Representative western blot images showing the relative protein levels of hexokinase 2 and PFKFB3. **G, H)** Quantitative analyses of hexokinase 2 and PFKFB3 standardized to β-actin. **I**) HK-2 cells were transfected with NC pcDNA3.0 and ATG5 pcDNA3.0. Representative western blot images showing the relative protein levels of hexokinase 2 and PFKFB3. **J, K)** Quantitative analyses of hexokinase 2 and PFKFB3 standardized to β-actin. n=3 per group. Data are expressed as mean ± SEM. ***P*<0.01, ****P*<0.001, *****P*<0.0001.


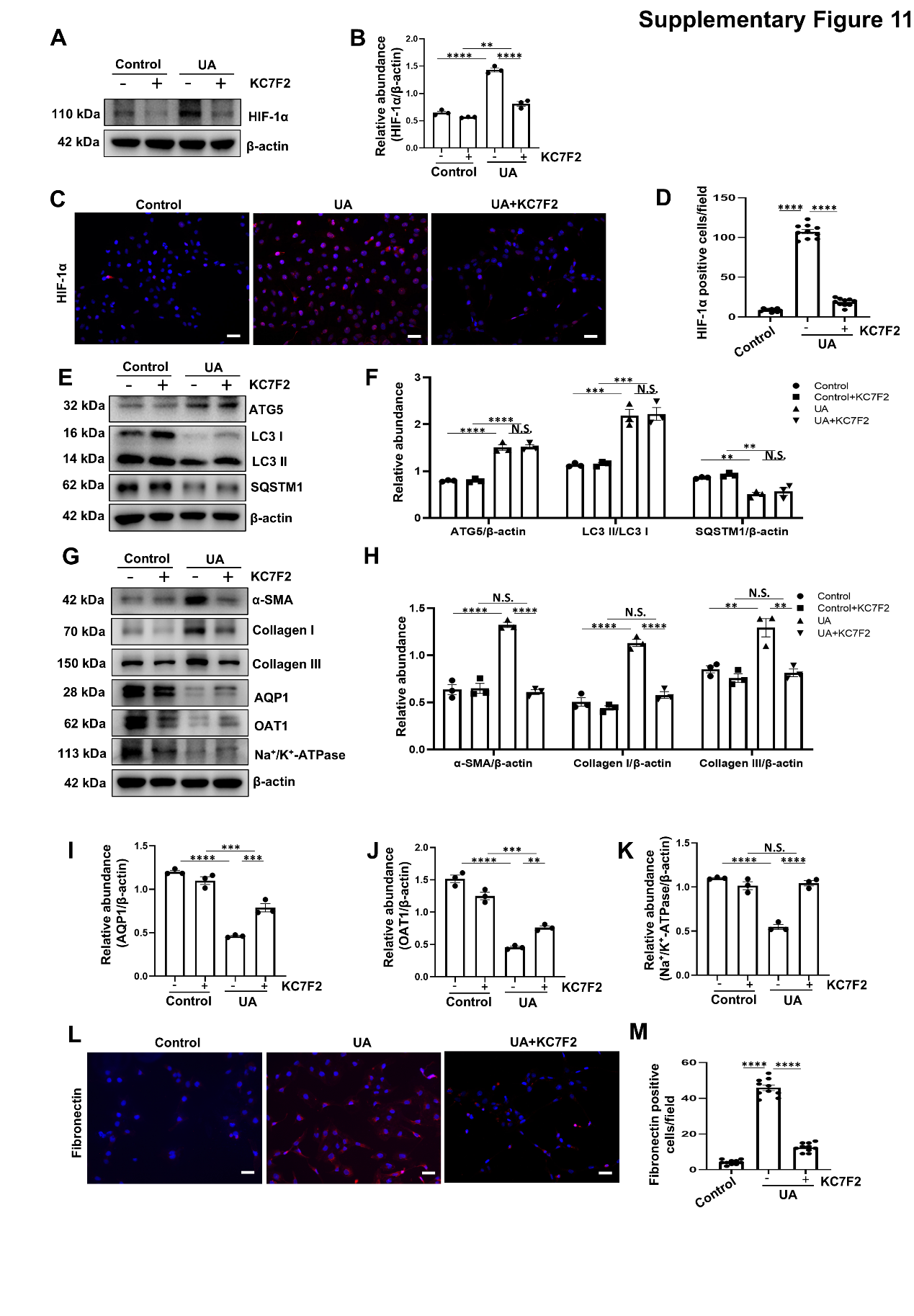


**Supplementary Figure 11. Inhibition of HIF-1α** **suppresses fibrosis-related proteins accumulation in HK-2 cells induced by UA**. **A, B)** HK-2 cells were treated with KC7F2 (5 μM) for 36 h with or without UA (800 μM) exposure. Western blot analyses and quantitative analyses showing the protein level of HIF-1α. **C, D)** Representative photomicrographs and quantifications showing HIF-1α expression in HK-2 cells treated as in (**A**). Scale bar = 50 µm. **E)** Western blot for ATG5, LC3 and SQSTM1 in HK-2 cells treated as in (**A**). **F)** Quantitative analyses of ATG5, LC3 II/I, and SQSTM1. **G)** Western blot images showing the relative protein levels of α-SMA, collagen I, collagen III, AQP1, OAT1, and Na^+^/K^+^-ATPase. **H-K)** Quantitative analyses of α-SMA, collagen I, collagen III, AQP1, OAT1, and Na^+^/K^+^-ATPase standardized to β-actin. **L, M)** Representative photomicrographs and quantification showing fibronectin expression in HK-2 cells treated as in (**A**). Scale bar = 50 µm. n=3 per group. Data are expressed as mean ± SEM. ***P*<0.01, ****P*<0.001, *****P*<0.0001, and N.S. denote statistically not significant.


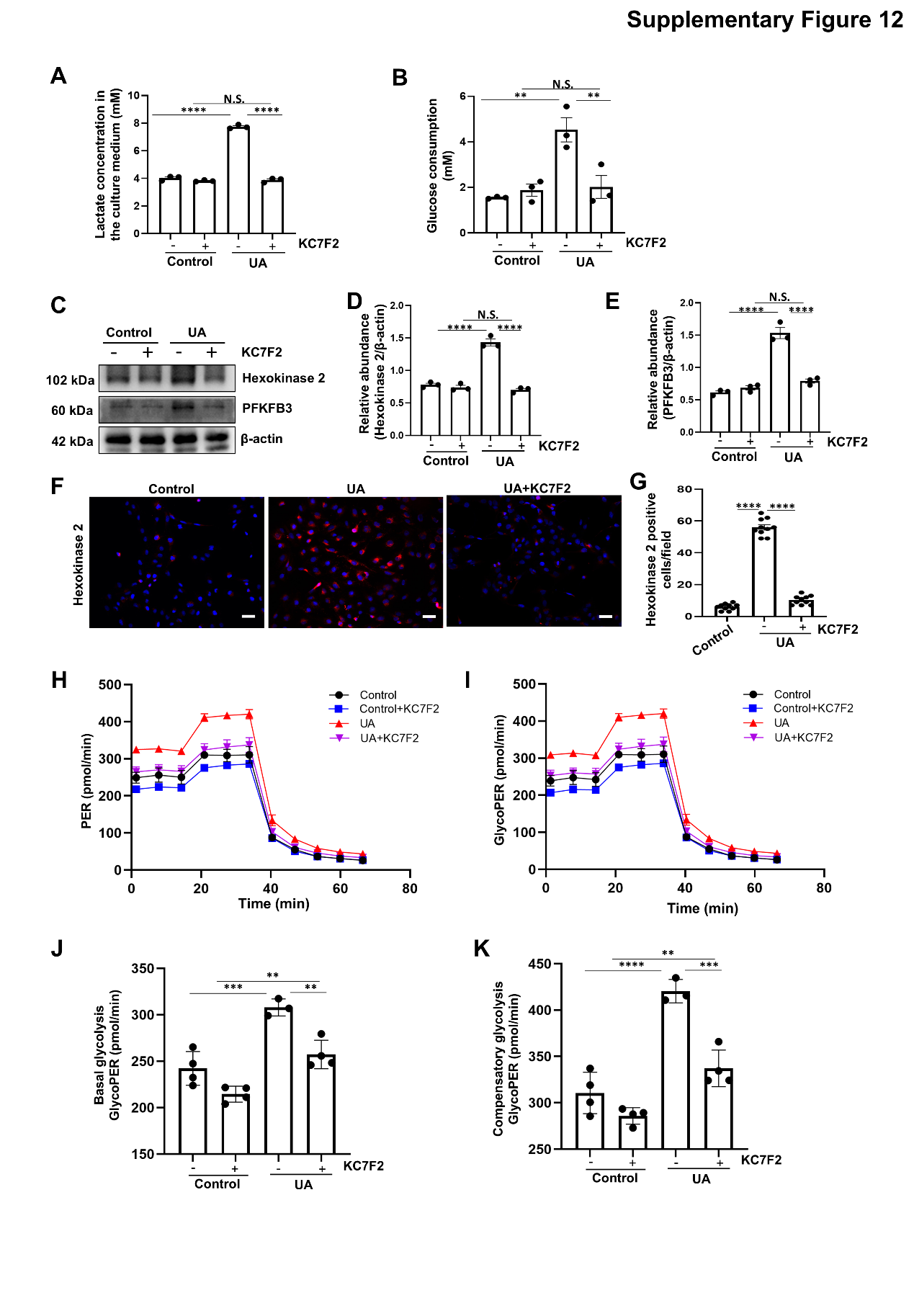


**Supplementary Figure 12. Inhibition of HIF-1α** **suppresses glycolysis in HK-2 cells induced by UA**. **A)** HK-2 cells were treated with KC7F2 (5 μM) for 36 h with or without UA (800 μM) exposure. Amount of lactate in culture medium of HK-2 cells. **B)** Glucose consumption by HK-2 cells treated as in (**A**). **C)** Representative western blot images showing the relative protein levels of hexokinase 2 and PFKFB3 in HK-2 cells treated as in (**A**). **D, E)** Quantitative analyses of hexokinase 2 and PFKFB3 standardized to β-actin. **F, G)** Representative photomicrographs and quantifications showing hexokinase 2 expression in HK-2 cells treated as in (**A**). Scale bar = 50 µm. **H-K)** PER, and glycoPER from basal glycolysis and compensatory glycolysis were measured by Seahorse Bioscience XF96 analyzer in HK-2 cells treated as in (**A**). n=3-4 per group. Data are expressed as mean ± SEM. ***P*<0.01, ****P*<0.001, *****P*<0.0001, and N.S. denote statistically not significant.


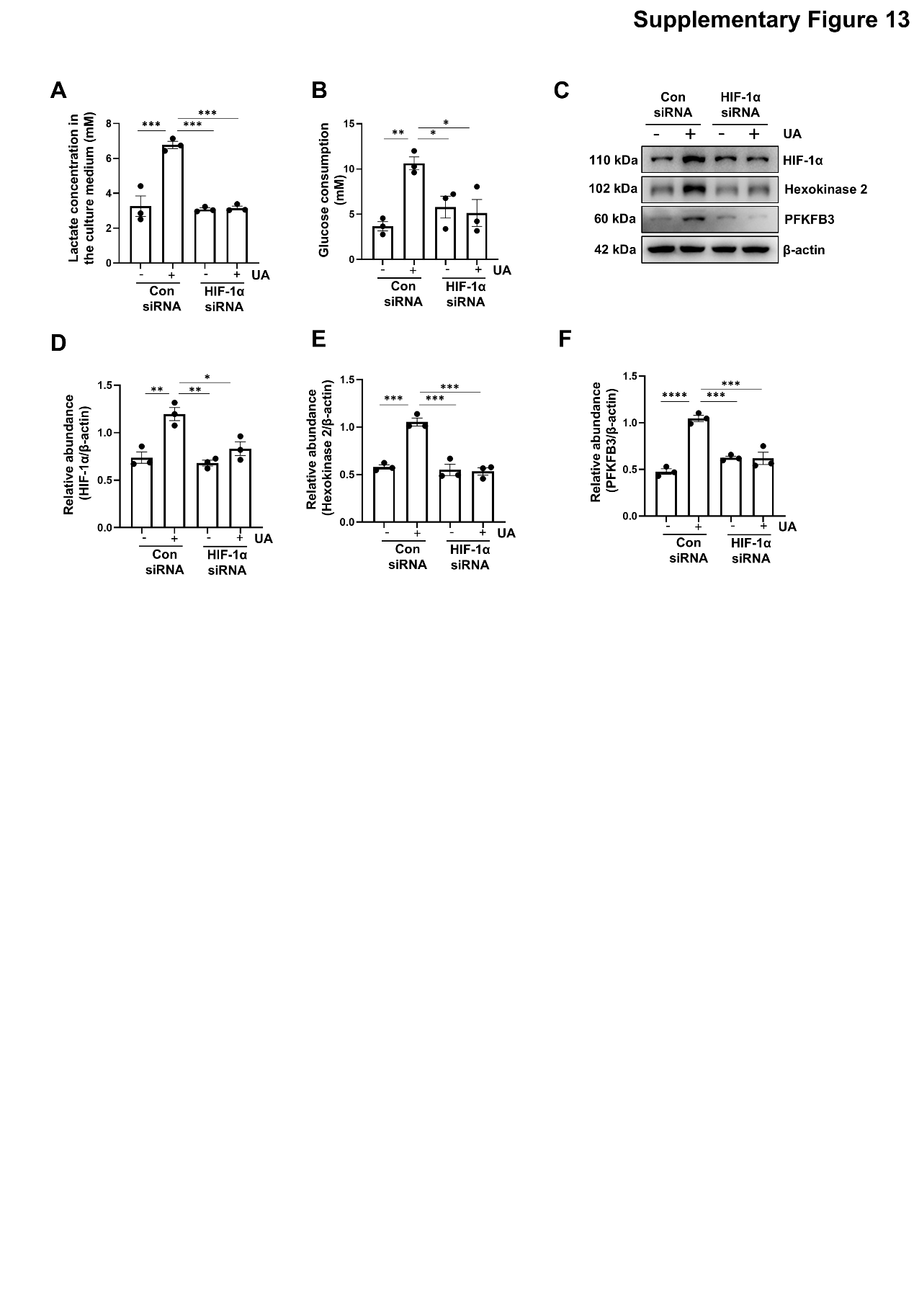


**Supplementary Figure 13. Transfection with HIF-1α** **siRNA** **suppresses glycolysis in HK-2 cells induced by UA**. **A)** HK-2 cells were transfected with HIF-1α siRNA or control siRNA and then incubated with or without UA (800 μM) for an additional 36 hours. Amount of lactate in culture medium of HK-2 cells. **B)** Glucose consumption by HK-2 cells treated as in (**A**). **C)** Representative western blot images showing the relative protein levels of HIF-1α, hexokinase 2 and PFKFB3 in HK-2 cells treated as in (**A**). **D-F)** Quantitative analyses of HIF-1α, hexokinase 2 and PFKFB3 standardized to β-actin. n=3 per group. Data are expressed as mean ± SEM. **P*<0.05, ***P*<0.01, ****P*<0.001, *****P*<0.0001.


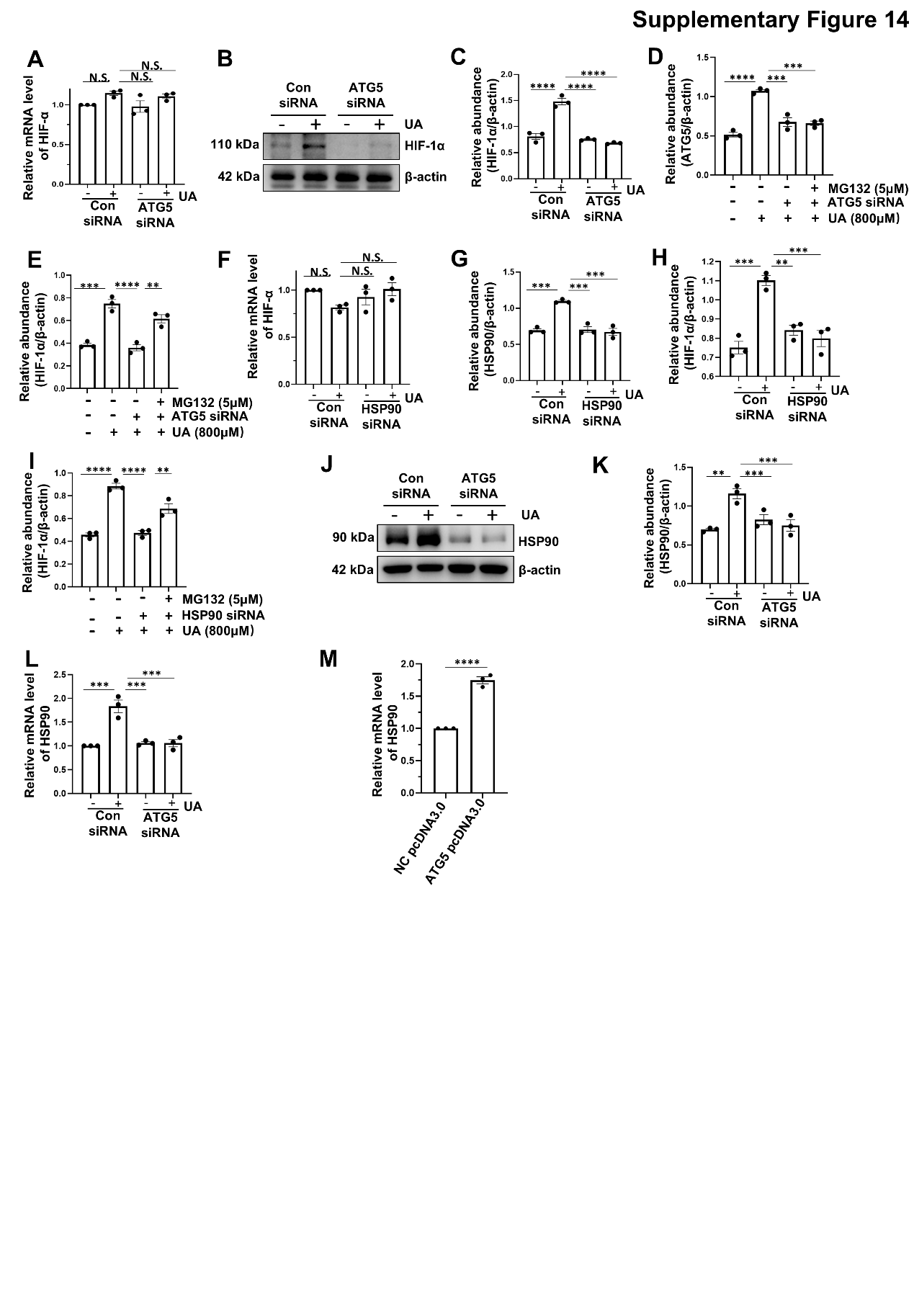


**Supplementary Figure 14. ATG5 enhances HSP90-HIF-1α interaction to promote HIF-1α stabilization. A**) HK-2 cells were transfected with ATG5 siRNA or control siRNA and then incubated with or without UA (800 μM) for an additional 36 hours. Relative mRNA level of HIF-1α in HK-2 cells from each group. **B, C**) Western blot analyses and quantitative analyses showing the expression of HIF-1α in HK-2 cells treated as in (**A**). **D, E)** HK-2 cells were transfected with ATG5 siRNA or control siRNA and then incubated with or without 800 μM UA for 36 hours, followed by treatment with 5 μM MG132 for 4 hours before being harvested. Quantitative analyses of ATG5 and HIF-1α standardized to β-actin. **F**) HK-2 cells were transfected with HSP90 siRNA or control siRNA and then incubated with or without UA (800 μM) for an additional 36 hours. Relative mRNA level of HIF-1α in HK-2 cells from each group. **G, H**) HK-2 cells were transfected with HSP90 siRNA or control siRNA and then incubated with or without UA (800 μM) for an additional 36 hours. Quantitative analyses of HSP90 and HIF-1α standardized to β-actin. **I)** HK-2 cells were transfected with HSP90 siRNA or control siRNA and then incubated with or without 800 μM UA for 36 hours, followed by treatment with 5 μM MG132 for 4 hours before being harvested. Quantitative analyses of HIF-1α standardized to β-actin. **J**) Western blot analyses showing the expression of HSP90 in HK-2 cells treated as in (**A**). **K)** Quantitative analyses of HSP90 standardized to β-actin. **L**) Relative mRNA level of HSP90 in HK-2 cells treated as in (**A**). **M**) HK-2 cells were transfected with NC pcDNA3.0 and ATG5 pcDNA3.0. Relative mRNA level of HSP90 in HK-2 cells. n=3 per group. Data are expressed as mean ± SEM. ***P*<0.01, ****P*<0.001, *****P*<0.0001, and N.S. denote statistically not significant.


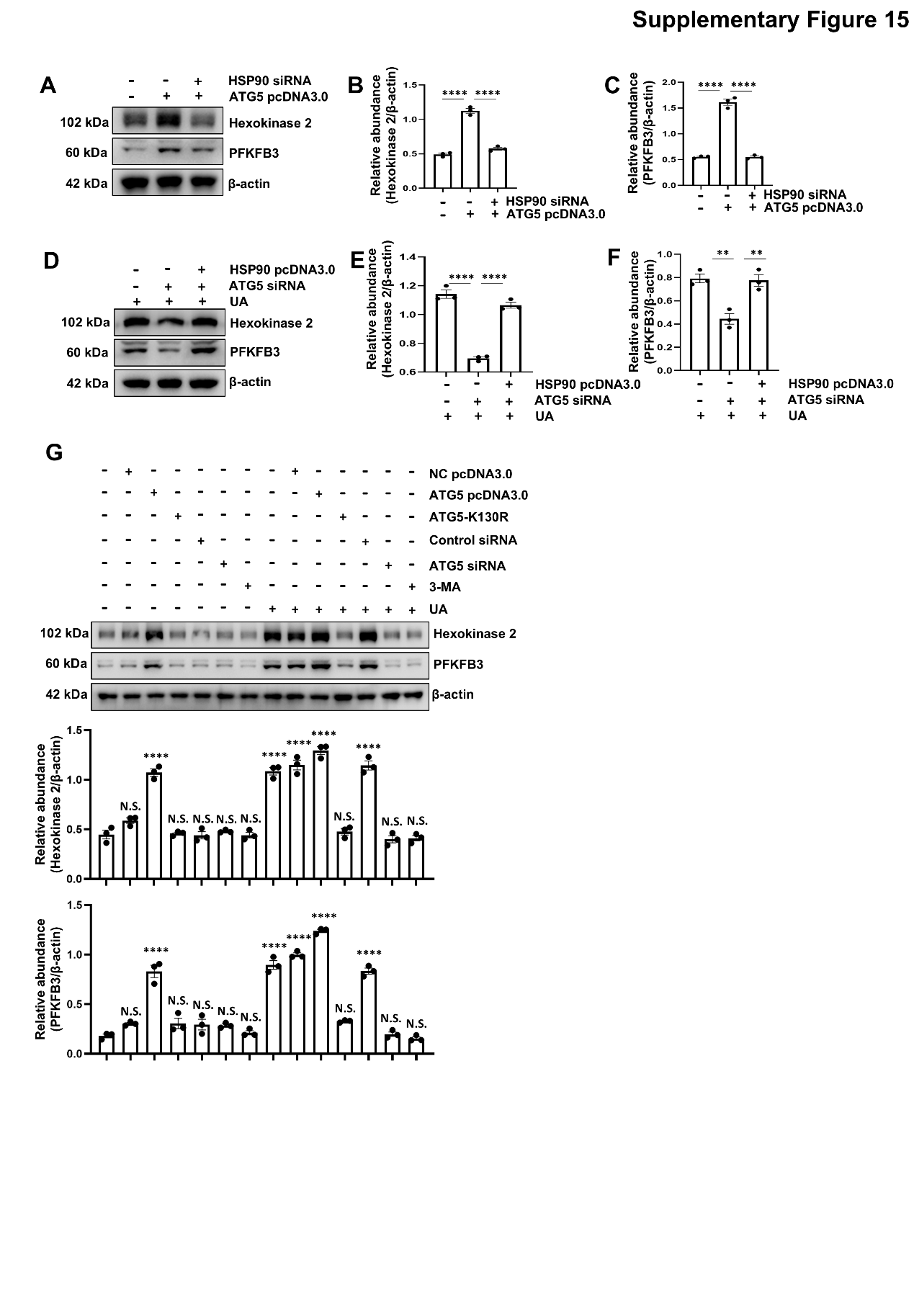


**Supplementary Figure 15. ATG5-mediated glycolysis is autophagy-dependent. A)** HK-2 cells were transfected with HSP90 siRNA or control siRNA for 36 hours, and then overexpressed ATG5 for an additional 36 hours. Western blot analyses showing the expression of hexokinase 2 and PFKFB3. **B, C)** Quantitative analyses of hexokinase 2 and PFKFB3 standardized to β-actin. **D)** HK-2 cells were transfected with ATG5 siRNA or control siRNA for 36 hours, and then overexpressed HSP90 for an additional 36 hours, followed by treatment with 800 μM UA for 36 hours before being harvested. Western blot analyses showing the expression of hexokinase 2 and PFKFB3. **E, F)** Quantitative analyses of hexokinase 2 and PFKFB3 standardized to β-actin. **G**) HK-2 cells were treated as indicated, the first group served as the control, and the subsequent 13 groups were compared to the control. n=3 per group. Data are expressed as mean ± SEM. ***P*<0.01, *****P*<0.0001, and N.S. denote statistically not significant.


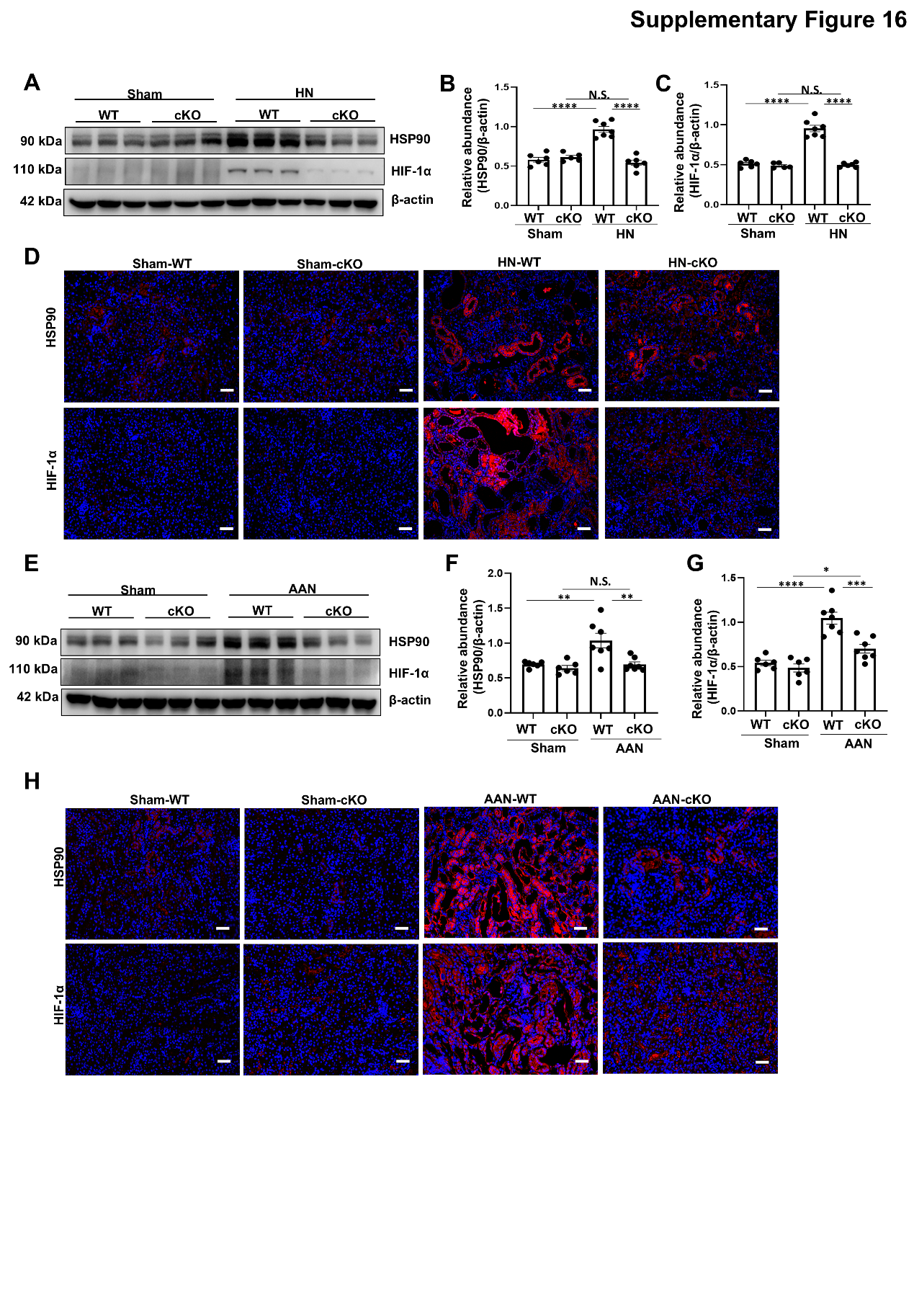


**Supplementary Figure 16. Tubule-specific ATG5 deletion reduced the protein level of HSP90 and HIF-1α in HN and AAN mice model**. **A-C)** Western blot and quantitative analyses for HSP90 and HIF-1α in HN mice model. **D)** Representative photomicrographs of immunofluorescence staining with HSP90 and HIF-1α in HN mice model. Scale bar = 50 µm. **E-G)** Western blot and quantitative analyses for HSP90 and HIF-1α in AAN mice model. **H)** Representative photomicrographs of immunofluorescence staining with HSP90 and HIF-1α in AAN mice model. Scale bar = 50 µm. n=5-7 per group. Data are expressed as mean ± SEM. **P*<0.05, ***P*<0.01, ****P*<0.001, *****P*<0.0001, and N.S. denote statistically not significant.


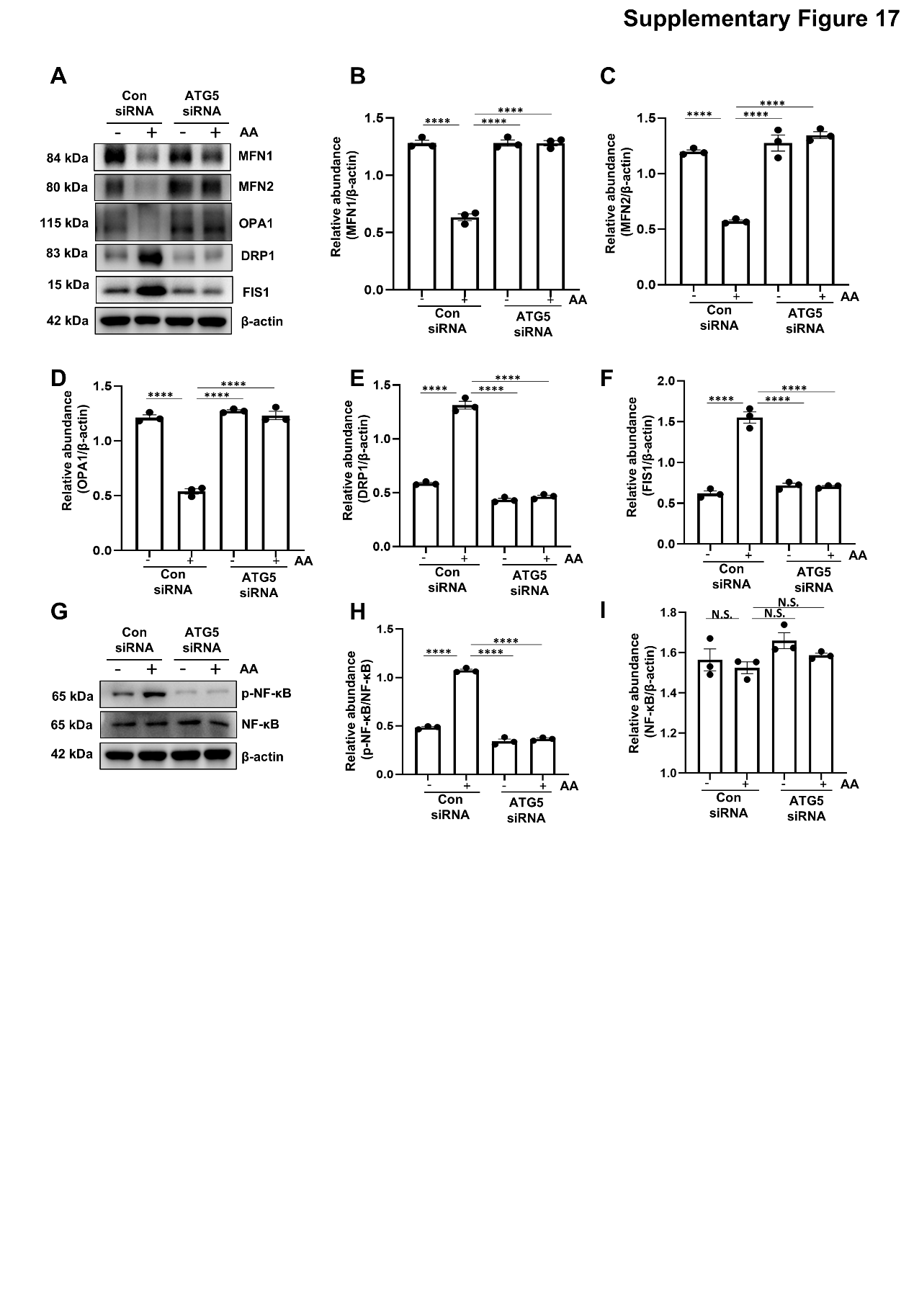


**Supplementary Figure 17. Gene silence of ATG5 inhibits mitochondrial fission and inflammation in HK-2 cells induced by AA**. **A)** HK-2 cells were transfected with ATG5 siRNA or control siRNA and then incubated with or without AA (5 μg/mL) for an additional 36 hours. Representative western blot images showing the relative protein levels of MFN1, MFN2, OPA1, DRP1 and FIS1. **B-F)** Quantitative analyses of MFN1, MFN2, OPA1, DRP1 and FIS1 standardized to β-actin. **G)** Western blot analyses for p-NF-κB and NF-κB in HK-2 cells treated as in (**A**) **H)** Quantitative analyses showing the ratio of p-NF-κB to NF-κB. **I)** Quantitative analyses of NF-κB standardized to β-actin. n=3 per group. Data are expressed as mean ± SEM. *****P*<0.0001, and N.S. denote statistically not significant.


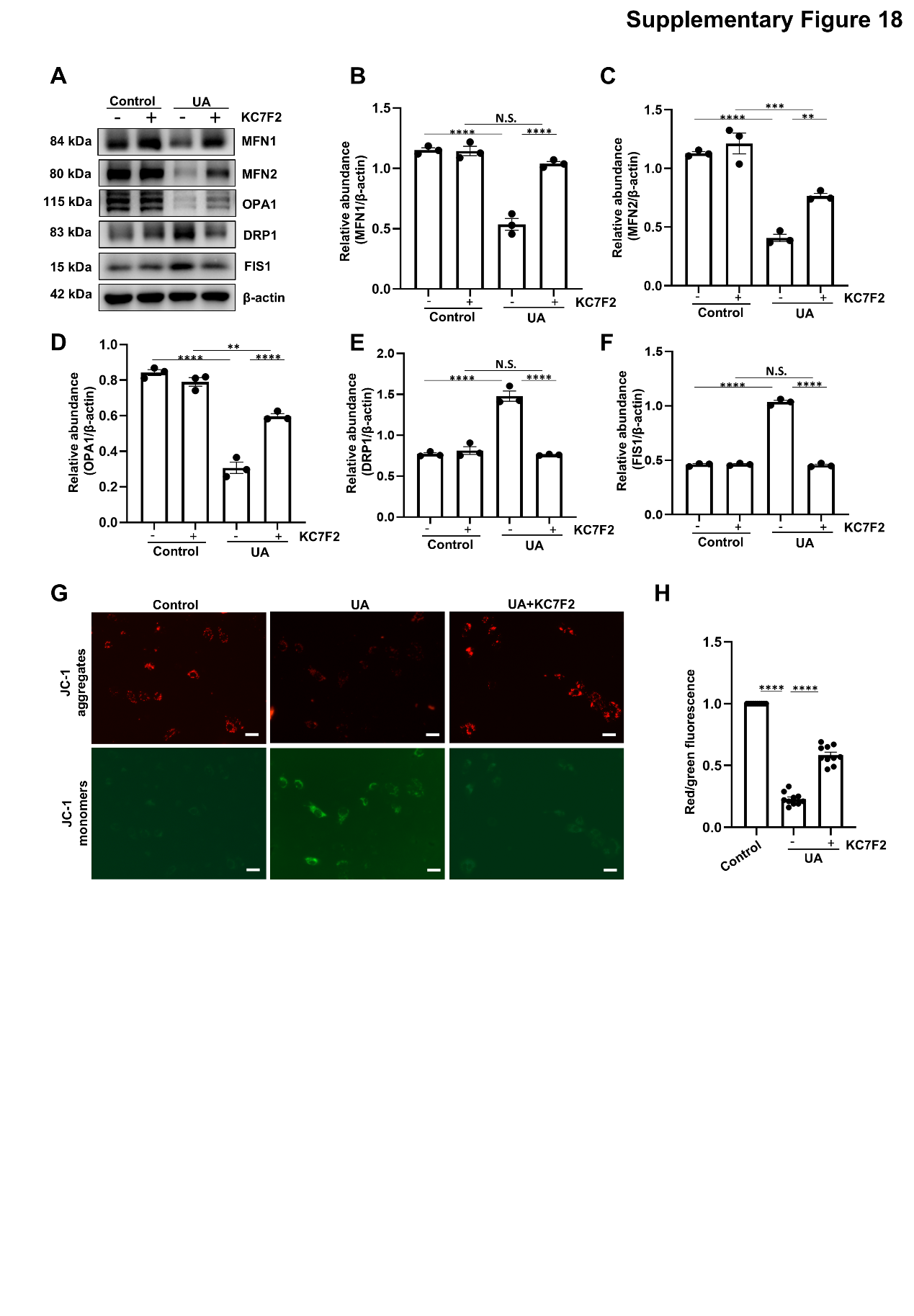


**Supplementary Figure 18. Inhibition of HIF-1α** **suppresses mitochondrial fission in HK-2 cells induced by UA**. **A)** HK-2 cells were treated with KC7F2 (5 μM) for 36 h with or without UA (800 μM) exposure. Representative western blot images showing the relative protein levels of MFN1, MFN2, OPA1, DRP1 and FIS1. **B-F)** Quantitative analyses of MFN1, MFN2, OPA1, DRP1 and FIS1 standardized to β-actin. **G)** JC-1 staining was performed to examine the mitochondrial membrane potential in HK-2 cells treated as in (**A**). Scale bar = 50 µm. **H)** Ratio of red/green fluorescence intensity was calculated. n=3 per group. Data are expressed as mean ± SEM. ***P*<0.01, ****P*<0.001, *****P*<0.0001, and N.S. denote statistically not significant.


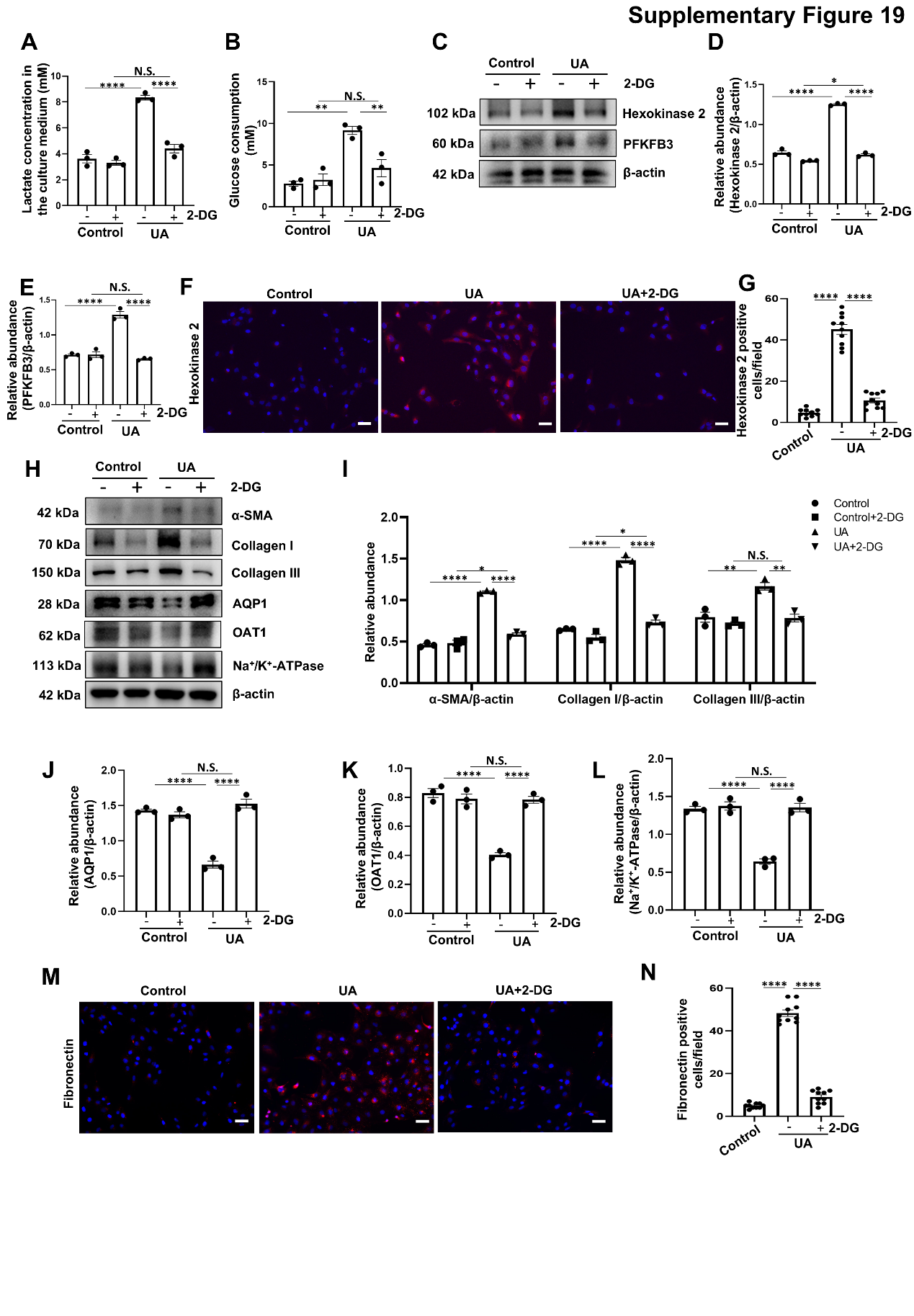


**Supplementary Figure 19. 2-DG inhibits glycolysis and attenuates fibrosis-related proteins accumulation in HK-2 cells induced by UA**. **A)** HK-2 cells were treated with 2-DG (2 mM) for 36 h with or without UA (800 μM) exposure. Amount of lactate in culture medium of HK-2 cells. **B)** Glucose consumption by HK-2 cells treated as in (**A**). **C)** Representative western blot images showing the relative protein levels of hexokinase 2 and PFKFB3 in HK-2 cells treated as in (**A**). **D, E)** Quantitative analyses of hexokinase 2 and PFKFB3 standardized to β-actin. **F, G)** Representative photomicrographs and quantifications showing hexokinase 2 expression in HK-2 cells treated as in (**A**). Scale bar = 50 µm. **H)** Western blot images showing the relative protein levels of α-SMA, collagen I, collagen III, AQP1, OAT1, and Na^+^/K^+^-ATPase. **I-L)** Quantitative analyses of α-SMA, collagen I, collagen III, AQP1, OAT1, and Na^+^/K^+^-ATPase standardized to β-actin. **M, N)** Representative photomicrographs and quantification showing fibronectin expression in HK-2 cells treated as in (**A**). Scale bar = 50 µm. n=3 per group. Data are expressed as mean ± SEM. **P*<0.05, ***P*<0.01, *****P*<0.0001, and N.S. denote statistically not significant.


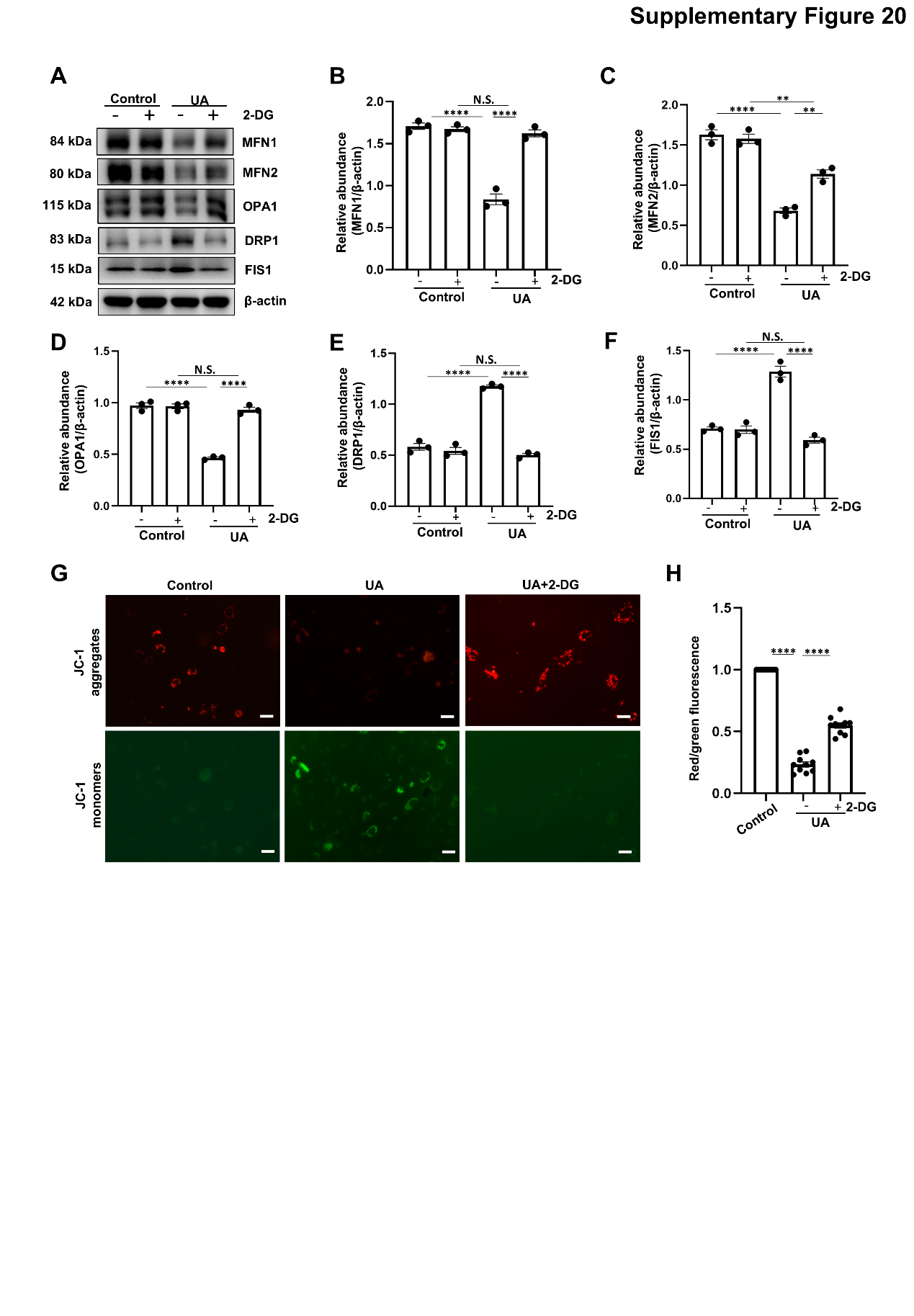


**Supplementary Figure 20. 2-DG inhibits mitochondrial fission in HK-2 cells induced by UA**. **A)** HK-2 cells were treated with 2-DG (2 mM) for 36 h with or without UA (800 μM) exposure. Representative western blot images showing the relative protein levels of MFN1, MFN2, OPA1, DRP1 and FIS1. **B-F)** Quantitative analyses of MFN1, MFN2, OPA1, DRP1 and FIS1 standardized to β-actin. **G)** JC-1 staining was performed to examine the mitochondrial membrane potential in HK-2 cells treated as in (**A**). Scale bar = 50 µm. **H)** Ratio of red/green fluorescence intensity was calculated. n=3 per group. Data are expressed as mean ± SEM. ***P*<0.01, *****P*<0.0001, and N.S. denote statistically not significant.


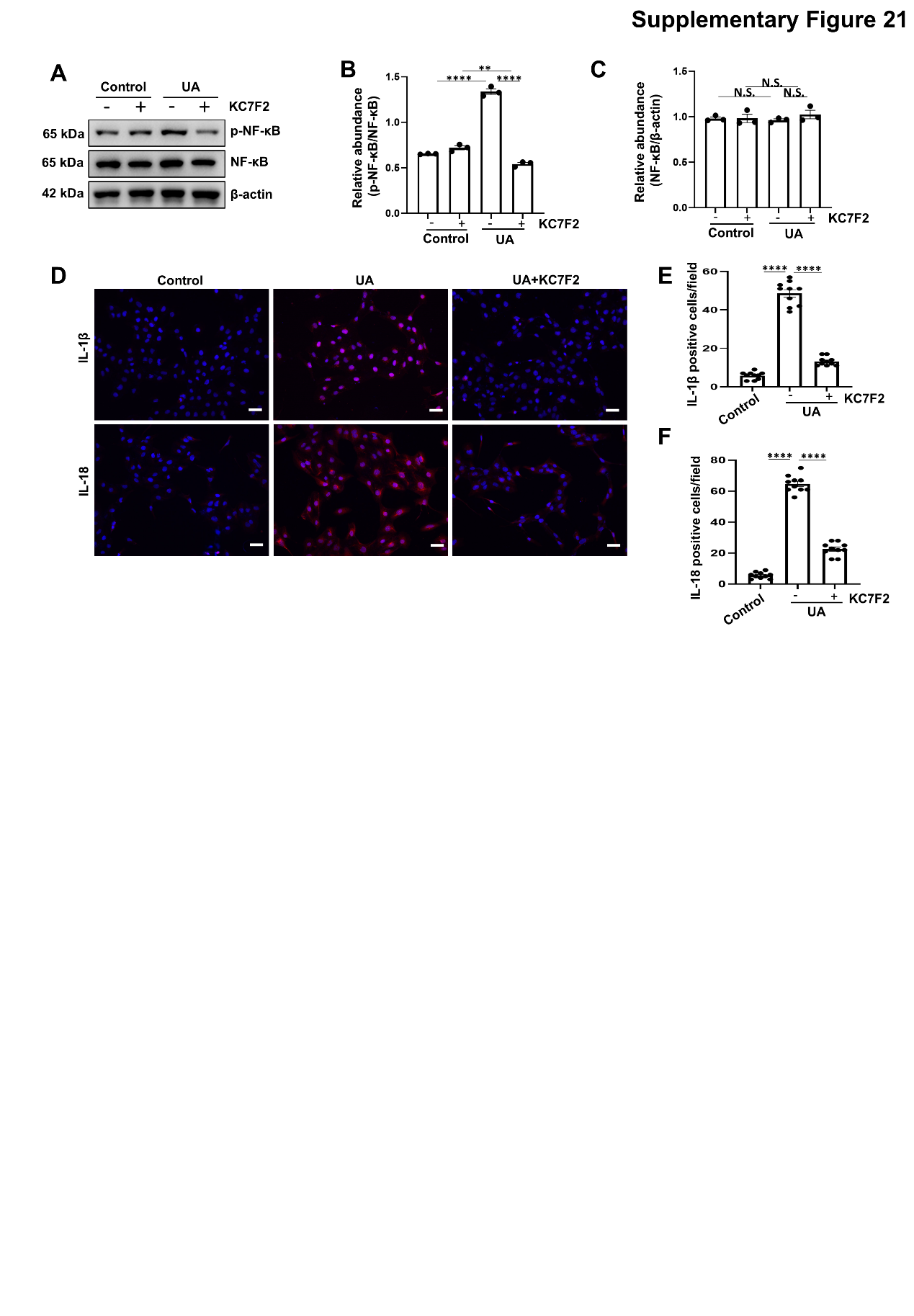


**Supplementary Figure 21. Inhibition of HIF-1α** **suppresses inflammation in HK-2 cells induced by UA**. **A)** Western blot analyses for p-NF-κB and NF-κB in HK-2 cells treated with KC7F2 (5 μM) for 36 h with or without UA (800 μM) exposure. **B)** Quantitative analyses showing the ratio of p-NF-κB to NF-κB. **C)** Quantitative analyses of NF-κB standardized to β-actin. **D-F)** Representative photomicrographs and quantifications showing IL-1β and IL-18 expression in HK-2 cells treated as in (**A**). Scale bar = 50 µm. n=3 per group. Data are expressed as mean ± SEM. ***P*<0.01, *****P*<0.0001, and N.S. denote statistically not significant.


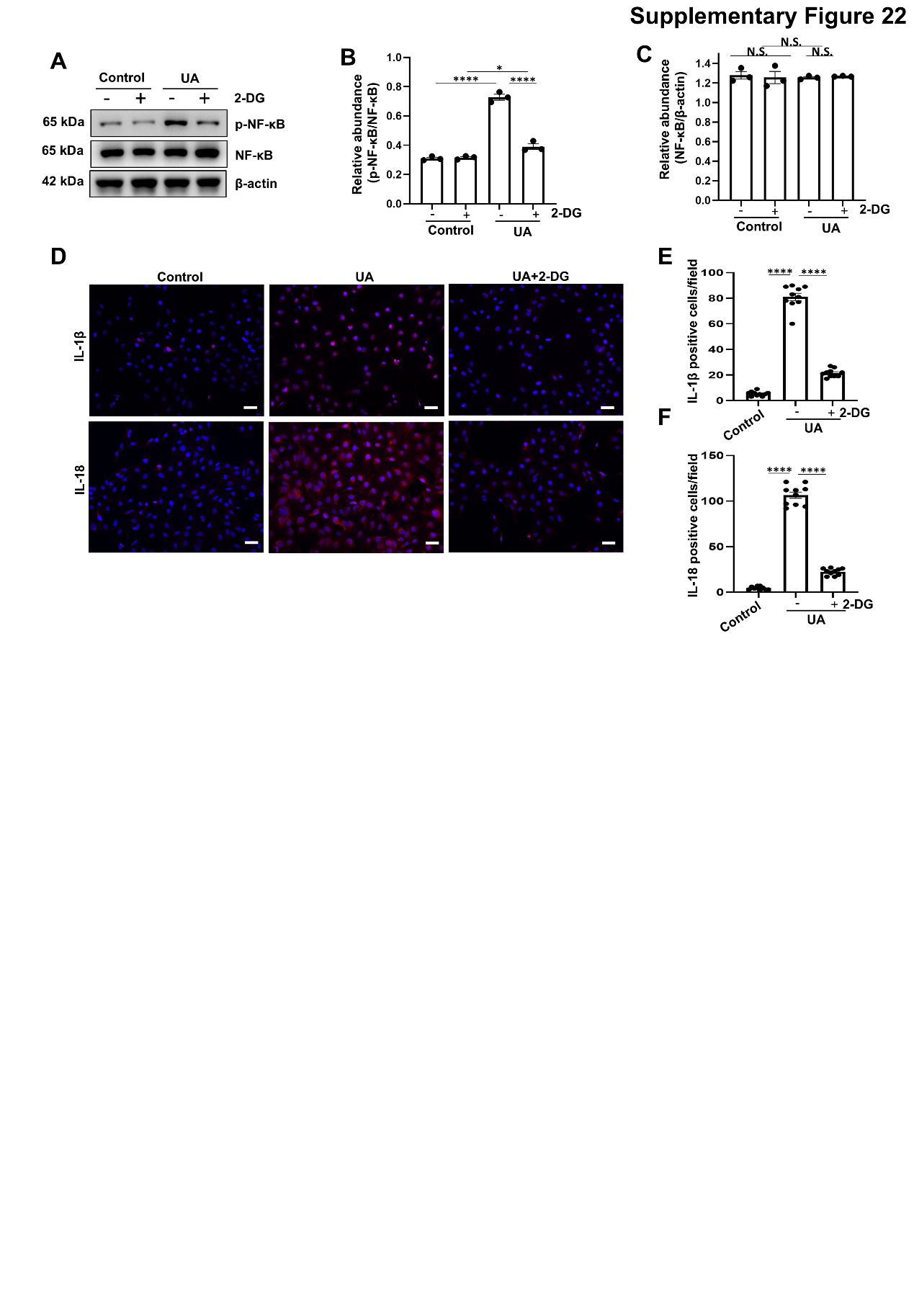


**Supplementary Figure 22. 2-DG inhibits inflammation in HK-2 cells induced by UA**. **A)** Western blot analyses for p-NF-κB and NF-κB in HK-2 cells treated with 2-DG (2 mM) for 36 h with or without UA (800 μM) exposure. **B)** Quantitative analyses showing the ratio of p-NF-κB to NF-κB. **C)** Quantitative analyses of NF-κB standardized to β-actin. **D-F)** Representative photomicrographs and quantifications showing IL-1β and IL-18 expression in HK-2 cells treated as in (**A**). Scale bar = 50 µm. n=3 per group. Data are expressed as mean ± SEM. **P*<0.05, *****P*<0.0001, and N.S. denote statistically not significant.


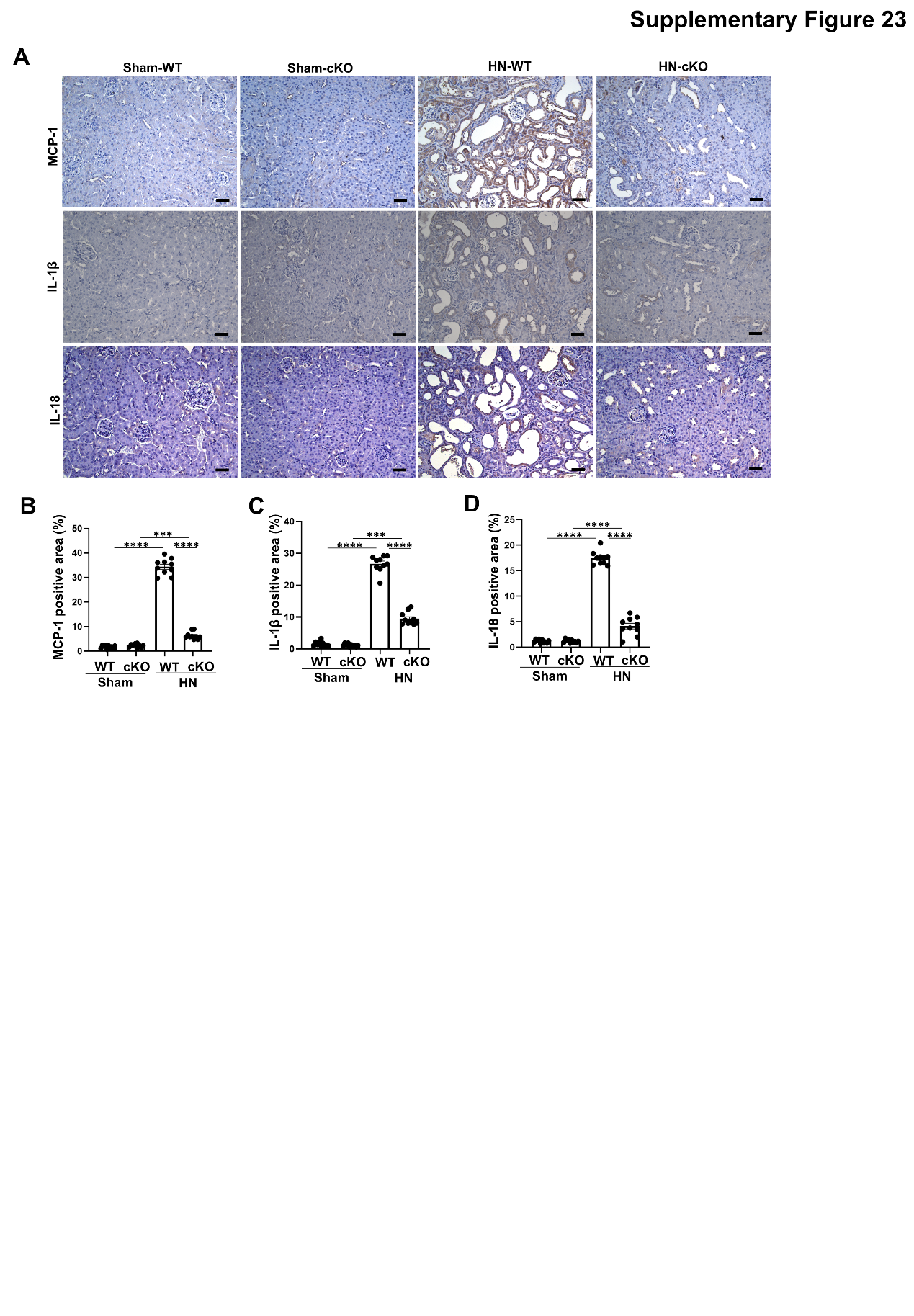


**Supplementary Figure 23. Tubule-specific ATG5 ablation ameliorates inflammation in HN mice model**. **A-D)** Representative photomicrographs and quantifications showing MCP-1, IL-1β and IL-18 expression in kidneys from different groups of mice. Scale bar = 50 µm. Data are expressed as mean ± SEM. ****P*<0.001, *****P*<0.0001.


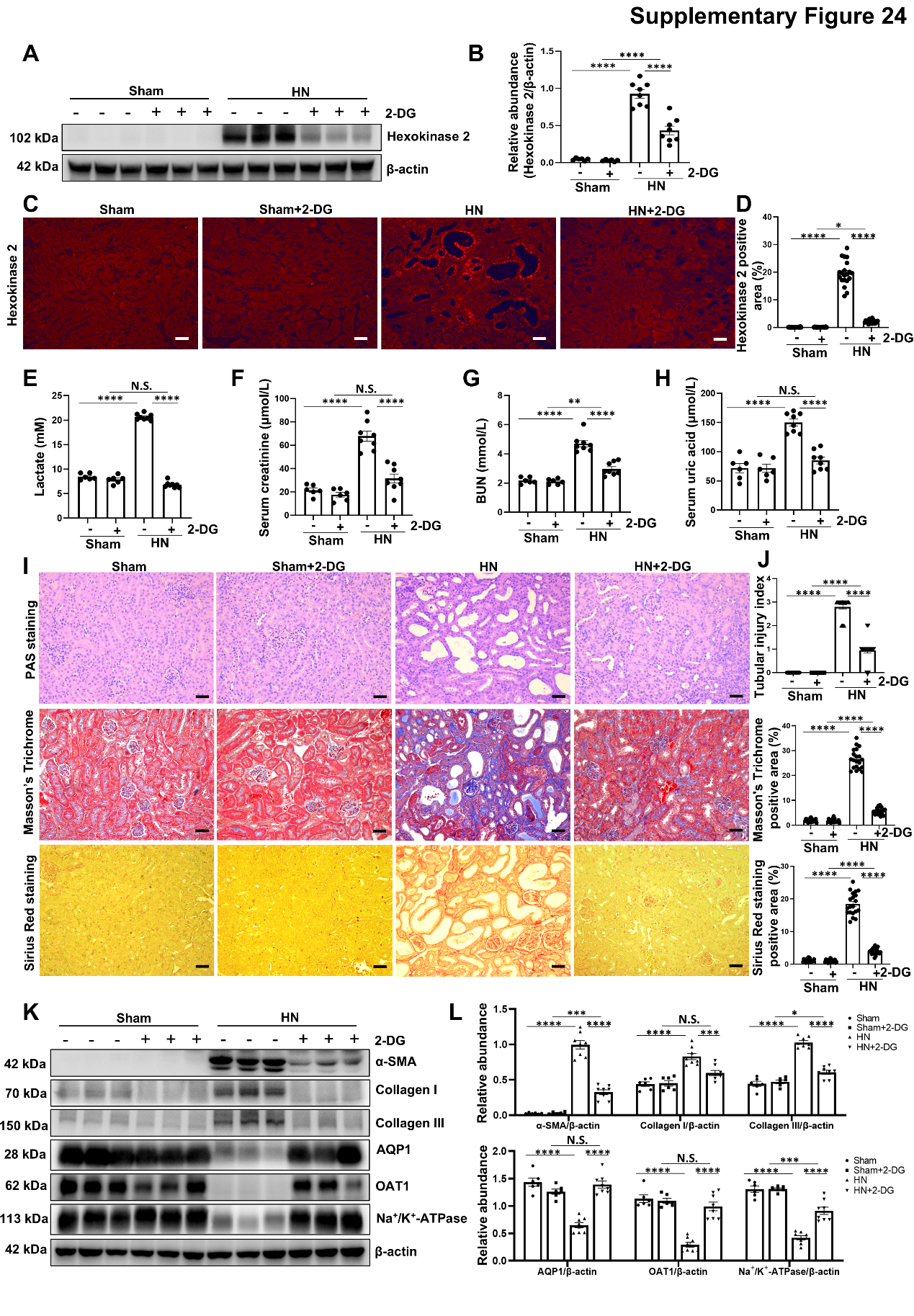


**Supplementary Figure 24. Administration with 2-DG inhibits glycolysis and kidney fibrosis in HN mice model. A)** Western blot for hexokinase 2 in kidneys from different groups of mice. **B)** Quantitative analyses of hexokinase 2 standardized to β-actin in kidneys from different groups of mice. **C, D)** Representative photomicrographs and quantification showing hexokinase 2 expression in kidneys from different groups of mice. Scale bar = 50 µm. **E-H)** Serum lactate, serum creatinine, BUN and serum uric acid from the mice in different groups. **I-J)** Representative images of PAS staining, Masson´s trichrome and Sirius red staining in kidneys from different groups of mice. Tubular injury index, Masson’s trichrome positive area and Sirius red staining positive area among groups as indicated. Scale bar = 50 µm. **K)** Western blot for α-SMA, collagen I, collagen III, AQP1, OAT1, and Na^+^/K^+^-ATPase in kidneys from different groups of mice. **L)** Quantitative analyses of α-SMA, collagen I, collagen III, AQP1, OAT1, and Na^+^/K^+^-ATPase standardized to β-actin in kidneys from different groups of mice. n=6-8 per group. Data are expressed as mean ± SEM. **P*<0.05, ***P*<0.01, ****P*<0.001, *****P*<0.0001, and N.S. denote statistically not significant.


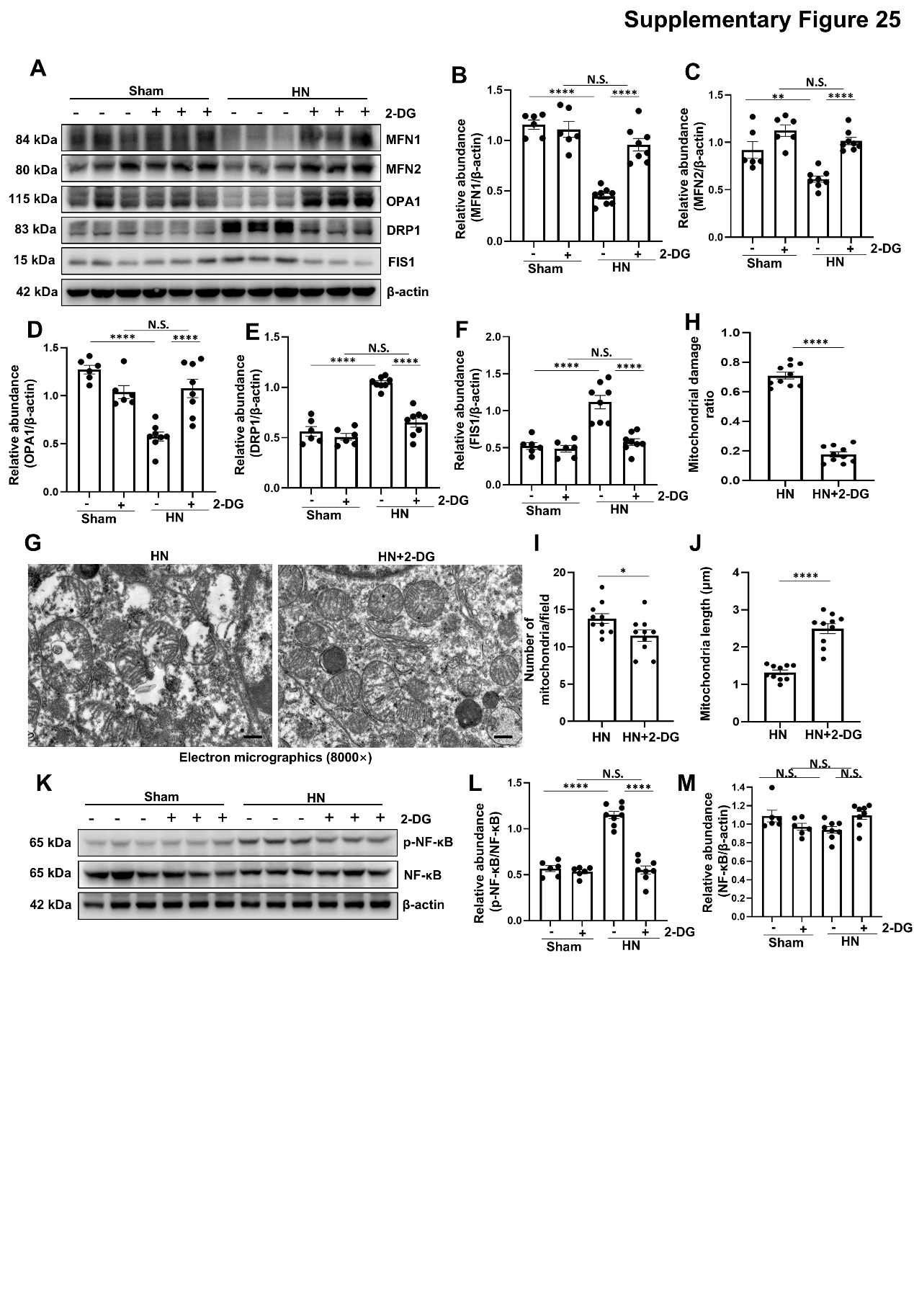


**Supplementary Figure 25. Administration with 2-DG inhibits mitochondrial fission and inflammation in HN mice model**. **A)** Western blot for MFN1, MFN2, OPA1, DRP1 and FIS1 in kidneys from different groups of mice. **B-F)** Quantitative analyses of MFN1, MFN2, OPA1, DRP1 and FIS1 standardized to β-actin in kidneys from different groups of mice. **G)** Representative transmission electron microscopy images showing the ultrastructural feature of mitochondria in kidneys from HN group and HN+2-DG group. Scale bar = 500 nm. **H)** Ratio of damaged mitochondria according to the transmission electron microscopy images. **I)** Quantitative analysis of the number of mitochondria per field. **J)** Quantitative analysis showing mitochondrial length in each group. **K)** Western blot analyses for p-NF-κB and NF-κB in kidneys from different groups of mice. **L)** Quantitative analyses showing the ratio of p-NF-κB to NF-κB. **M)** Quantitative analyses of NF-κB standardized to β-actin. n=6-8 per group. Data are expressed as mean ± SEM. **P*<0.05, ***P*<0.01, *****P*<0.0001, and N.S. denote statistically not significant.


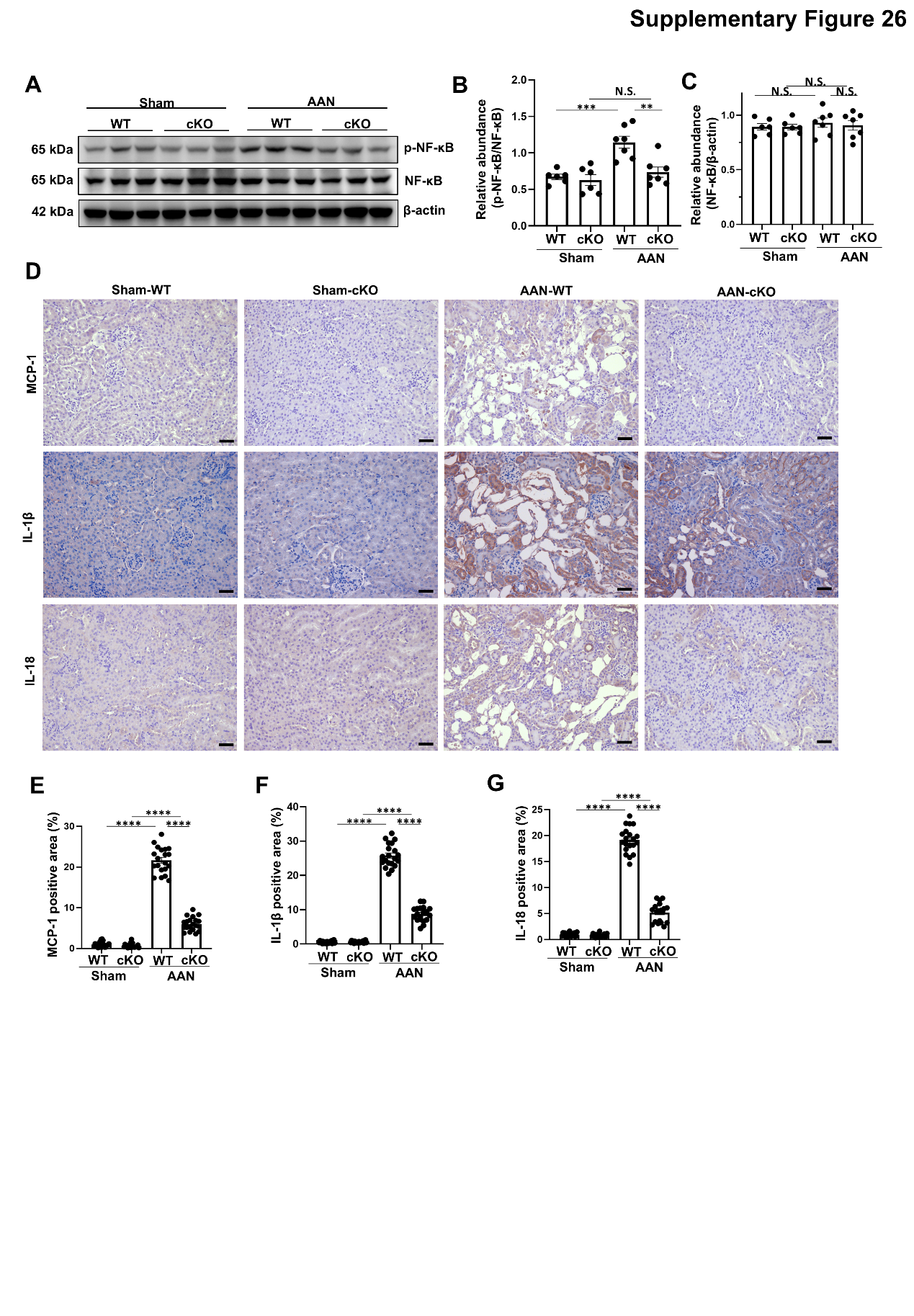


**Supplementary Figure 26. Tubule-specific ATG5 ablation ameliorates inflammation in AAN mice model**. **A)** Western blot analyses for p-NF-κB and NF-κB in kidneys from different groups of mice. **B)** Quantitative analyses showing the ratio of p-NF-κB to NF-κB. **C)** Quantitative analyses of NF-κB standardized to β-actin. **D-G)** Representative photomicrographs and quantifications showing MCP-1, IL-1β and IL-18 expression in kidneys from different groups of mice. Scale bar = 50 µm. n=6-7 per group. Data are expressed as mean ± SEM. ***P*<0.01, ****P*<0.001, *****P*<0.0001, and N.S. denote statistically not significant.


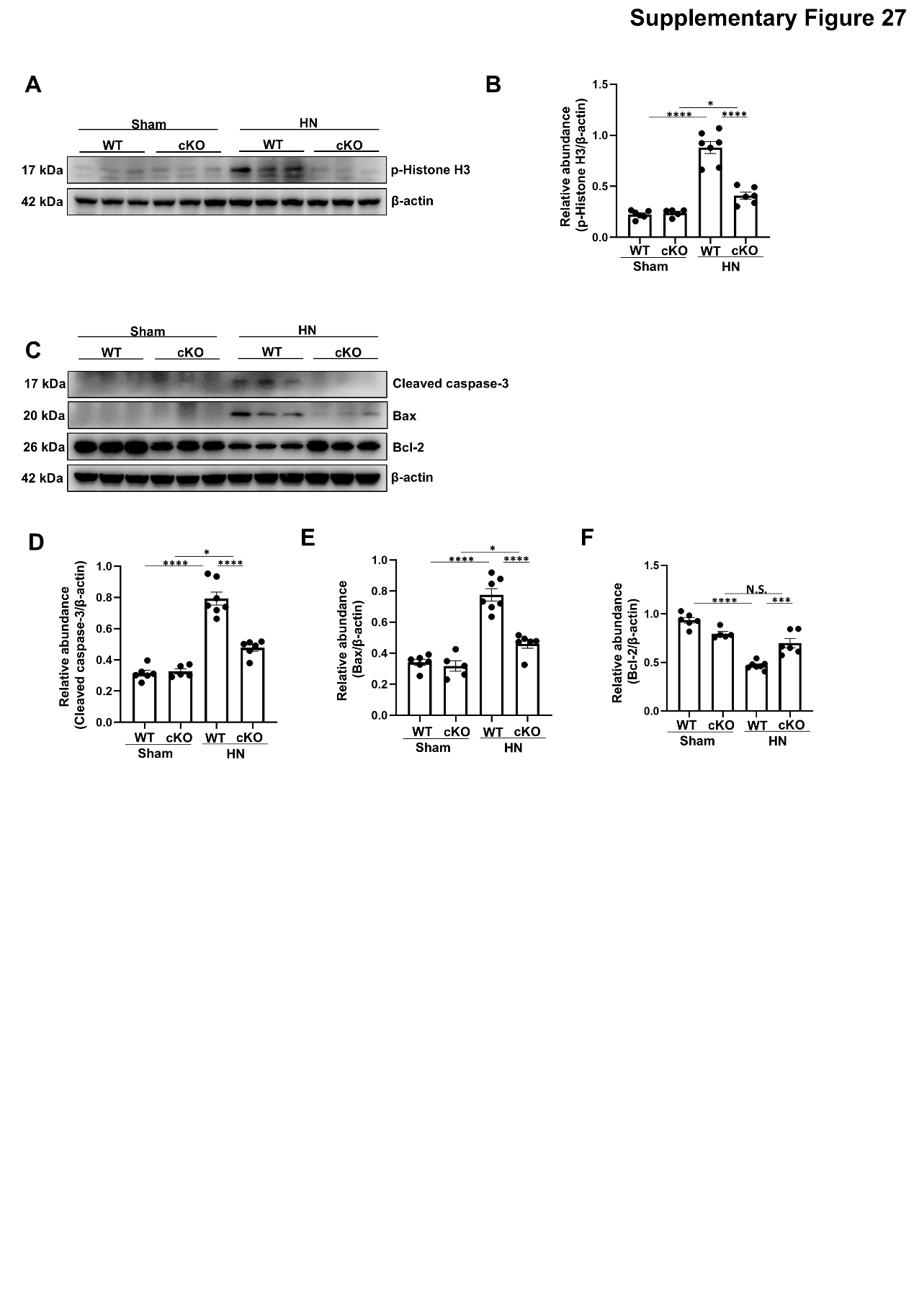


**Supplementary Figure 27. Tubule-specific ATG5 ablation ameliorates cell cycle arrest and apoptosis in HN mice model**. **A)** Western blot analyses for p-Histone H3 in kidneys from different groups of mice. **B)** Quantitative analyses of p-Histone H3 standardized to β-actin. **C)** Western blot analyses for cleaved caspase-3, bax, and bcl-2 in kidneys from different groups of mice. **D-F)** Quantitative analyses of cleaved caspase-3, bax, and bcl-2 standardized to β-actin. n=5-7 per group. Data are expressed as mean ± SEM. **P*<0.05, ****P*<0.001, *****P*<0.0001, and N.S. denote statistically not significant.

**Table S1. Clinical characteristics of control group and IgAN patients**

| Variables | Control (n=11) | IgAN (n=20) | *P* value |
| --- | --- | --- | --- |
| **General data** |  |  |  |
| Sex (male) | 9 (81.8%) | 11 (55.0%) | 0.241 |
| Age (years) | 62.3±10.1 | 44.8±16.5 | 0.003 |
| BMI (kg/m^2^) | 24.6±3.0 | 23.7±3.1 | 0.457 |
| Systolic pressure (mmHg) | 136.6±12.3 | 134.5±20.8 | 0.722 |
| Diastolic pressure (mmHg) | 84.9±9.0 | 85.1±9.8 | 0.968 |
| Smoking (n, %) | 1 (9.1%) | 7 (35.0%) | 0.203 |
| Drinking (n, %) | 1 (9.1%) | 3 (15.0%) | 0.553 |
| Hypertension (n, %) | 2 (18.2%) | 8 (40.0%) | 0.262 |
| Diabetes mellitus (n, %) | 1 (9.1%) | 2 (10.0%) | 0.719 |
| **Laboratory examination** |  |  |  |
| Glucose (mmol/L) | 5.5±1.1 | 5.1±0.7 | 0.167 |
| Glycosylated hemoglobin (%) | 6.2±0.8 | 5.8±0.5 | 0.163 |
| Hemoglobin (g/L) | 139.9±12.6 | 126.9±18.2 | 0.044 |
| Serum albumin (g/L) | 44.3±3.7 | 38.6±6.4 | 0.010 |
| Serum globulin (g/L) | 24.8±3.2 | 23.8±3.0 | 0.412 |
| ALT (U/L) | 20.3±13.6 | 17.4±8.6 | 0.474 |
| AST (U/L) | 19.5±9.8 | 17.4±7.9 | 0.551 |
| Urea (mmol/L) | 5.1±1.0 | 8.0±4.4 | 0.010 |
| Creatinine (μmol/L) | 77.2±14.3 | 147.5±91.2 | 0.003 |
| Uric acid (μmol/L) | 308.7±70.3 | 432.1±135.7 | 0.009 |
| Cystatin C (mg/L) | 1.0±0.2 | 1.5±0.6 | 0.005 |
| eGFR (mL/min/1.73m^2^) | 88.6±9.8 | 61.2±30.3 | 0.007 |
| Triglyceride (mmol/L) | 1.7±0.5 | 1.8±0.8 | 0.887 |
| Cholesterol (mmol/L) | 4.5±0.8 | 4.8±1.3 | 0.584 |
| **Therapy** |  |  |  |
| Lipid-lowering medicine (n, %) | 0 (0.0%) | 7 (35.0%) | 0.033 |
| CCB (n, %) | 1 (9.1%) | 6 (30.0%) | 0.372 |
| Glucocorticoid (n, %) | 0 (0.0%) | 10 (50.0%) | 0.005 |

Abbreviations: BMI, body mass index; ALT, alanine aminotransferase; AST, aspartate aminotransferase; eGFR, estimated glomerular filtration rate; CCB, calcium channel blockers. The comparisons of normally distributed data were conducted using the unpaired two-tailed Student's t-test and the results are expressed as the mean ± SD. Categorical variables were analyzed using the chi-squared (χ²) test, and these data are presented as percentages. *P* value<0.05 were considered to be statistically significant.

**Table S2. Antibodies used in this study**

| Primary antibodies | Source | Provider | Catalog | Application |
| --- | --- | --- | --- | --- |
| α-SMA | Mouse | Sigma-Aldrich | A2547 | WB (1:1000)  IF (1:100) |
| ATG5 | Rabbit | Abcam | ab109490 | WB (1:1000) |
| ATG5 | Rabbit | ABclonal | A0203 | IHC (1:100)  IF (1:100) |
| AQP1 | Rabbit | Servicebio | GB11310-1 | WB (1:1000) |
| β-actin | Mouse | Sangon Biotech | D191047 | WB (1:5000) |
| Bax | Mouse | BD Pharmingen | 556467 | WB (1:1000) |
| Bcl-2 | Rabbit | Bioworld | BS1511P | WB (1:1000) |
| Cleaved caspase-3 | Rabbit | Cell Signaling Technology | #9664 | WB (1:1000) |
| Collagen I | Rabbit | Santa Cruz Biotechnology | sc-393573 | WB (1:1000) |
| Collagen III | Rabbit | ABclonal | A0817 | WB (1:1000) |
| Collagen III | Rabbit | Servicebio | GB11023 | WB (1:1000) |
| DRP1 | Rabbit | Cell Signaling Technology | #5391 | WB (1:1000) |
| Fibronectin | Rabbit | Abcam | ab2413 | IF (1:100) |
| FIS1 | Rabbit | ABclonal | A5821 | WB (1:1000) |
| Hexokinase 2 | Rabbit | Abcam | ab209847 | WB (1:1000)  IHC (1:500)  IF (1:100) |
| HIF-1α | Rabbit | Abcam | ab228649 | WB (1:1000)  IP (2 µg)  IF (1:200) |
| HSP90 | Rabbit | Cell Signaling Technology | #4877 | WB (1:1000)  IF (1:200) |
| HSP90 | Rabbit | Santa Cruz Biotechnology | sc-13119 | IP (2 µg) |
| IgG | Rabbit | Cell Signaling Technology | #2729S | IP (1 µg) |
| IL-1β | Rabbit | Servicebio | GB11113 | IF (1:800)  IHC (1:800) |
| IL-18 | Rabbit | ABclonal | A16737 | IF (1:800)  IHC (1:800) |
| LC3 | Rabbit | Novus Biologicals | NB100-2220 | WB (1:1000) |
| MFN1 | Rabbit | ABclonal | A21293 | WB (1:1000) |
| MFN2 | Rabbit | ABclonal | A19678 | WB (1:1000) |
| MCP-1 | Rabbit | Servicebio | GB11199 | IHC (1:200) |
| NF-κB | Mouse | Santa Cruz Biotechnology | sc-8008 | WB (1:1000) |
| Na^+^/K^+^-ATPase | Rabbit | Servicebio | GB11400-1 | WB (1:1000) |
| OAT1 | Rabbit | Abbiotec | 250798 | WB (1:1000) |
| OPA1 | Rabbit | Proteintech | 27733-1-AP | WB (1:1000) |
| p-Histone H3 | Mouse | Millipore | #05-098 | WB (1:1000) |
| p-NF-κB | Mouse | Santa Cruz Biotechnology | sc-166748 | WB (1:1000) |
| PFKFB3 | Rabbit | Cell Signaling Technology | #13123 | WB (1:1000) |
| SQSTM1 | Rabbit | ABclonal | A19700 | WB (1:1000) |
| Ubiquitin | Rabbit | Cell Signaling Technology | #3933 | WB (1:1000) |

**Table S3. siRNA sequences used in this study are listed in the 5'-3' direction**

| siRNA | sequence (5’-3’) |
| --- | --- |
| ATG5 siRNA | 5ʹ-CCTTTGGCCTAAGAAGAAA-3ʹ |
| HIF-1α siRNA | 5ʹ-GCCGAGGAAGAACUAUGAATT-3ʹ |
| HSP90 siRNA | 5ʹ-AACCAUUGCCAAGUCUGGUACUAAA-3ʹ |
| Control siRNA | 5ʹ-UUCUCCGAACGUGUCACGUTT-3ʹ |

**Table S4. Primer sequences used in real-time PCR are listed in the 5'-3' direction**

| Name | 5’-Forward | 3’-Reverse |
| --- | --- | --- |
| HIF-1α | CAAGGCAGCAGAAACCTAC | GAGCCACCAGTGTCCAA |
| HSP90 | CCCAGAGTGCTGAATACCCG | TAACAGGTGCCCTGCTTCTC |
